# Heterostilbene Carbamates with Selective and Remarkable Butyrylcholinesterase Inhibition: Computational Study and Physico-Chemical Properties

**DOI:** 10.3390/biom15060825

**Published:** 2025-06-05

**Authors:** Anamarija Raspudić, Ilijana Odak, Milena Mlakić, Antonija Jelčić, Karla Bulava, Karla Karadža, Valentina Milašinović, Ivana Šagud, Paula Pongrac, Dora Štefok, Danijela Barić, Irena Škorić

**Affiliations:** 1 Department of Chemistry, Faculty of Science and Education, University of Mostar, Matice Hrvatske bb, 88 000 Mostar, Bosnia and Herzegovina; anamarija.raspudic@fpmoz.sum.ba (A.R.); ilijana.odak@fpmoz.sum.ba (I.O.); 2 Department of Organic Chemistry, Faculty of Chemical Engineering and Technology, University of Zagreb, Trg Marka Marulića 19, HR-10 000 Zagreb, Croatia; mdragojev@fkit.unizg.hr (M.M.); ajelcic@fkit.unizg.hr (A.J.); kbulava@fkit.hr (K.B.); kkaradza@fkit.hr (K.K.); 3 Laboratory for Chemical and Biological Crystallography, Division of Physical Chemistry, Ruđer Bošković Institute, Bijenička Cesta 54, HR-10 000 Zagreb, Croatia; valentina.milasinovic@irb.hr; 4 Croatian Agency for Medicinal Products and Medical Devices, Ksaverska Cesta 4, HR-10 000 Zagreb, Croatia; ivana.sagud@halmed.hr; 5 Faculty of Biotechnology and Drug Development, University of Rijeka, Radmile Matejčić 2, HR-51 000 Rijeka, Croatia; paula.pongrac@selvita.com (P.P.); dora.stefok@selvita.com (D.Š.); 6 Group for Computational Life Sciences, Division of Physical Chemistry, Ruđer Bošković Institute, Bijenička Cesta 54, HR-10 000 Zagreb, Croatia

**Keywords:** anti-inflammatory activity, butyrylcholinesterase inhibition, carbamates, docking, molecular dynamics, synthesis

## Abstract

This manuscript reports the synthesis and characterization of 19 novel heterostilbene carbamates, designed as selective butyrylcholinesterase (BChE) inhibitors with potential applications in the treatment of neurodegenerative disorders, particularly Alzheimer’s disease. The compounds were synthesized from resveratrol analogs, and their structures were confirmed by NMR spectroscopy, high-resolution mass spectrometry (HRMS), and single-crystal X-ray diffraction for selected derivatives (compounds **1** and **4**). In vitro assays demonstrated high selectivity toward BChE over acetylcholinesterase (AChE), with compound **16** exhibiting exceptional inhibitory activity (IC_50_ = 26.5 nM). Furthermore, compound **16** showed moderate anti-inflammatory effects by inhibiting LPS-stimulated TNF-α production in peripheral blood mononuclear cells. In silico ADME(T) profiling revealed favorable pharmacokinetic properties and low mutagenic potential for the majority of compounds. Molecular docking and molecular dynamics simulations confirmed stable binding interactions within the BChE active site. These results highlight heterostilbene carbamates as promising lead structures for developing novel therapeutic agents targeting neurodegenerative diseases.

## 1. Introduction

Carbamates are a group of compounds that have garnered increasing interest as potential therapeutic agents for treating neurodegenerative diseases. These compounds primarily function by inhibiting acetylcholinesterase (AChE), but they can also target butyrylcholinesterase (BChE), another cholinesterase enzyme involved in the hydrolysis of acetylcholine. While AChE is the primary enzyme responsible for acetylcholine breakdown in the central nervous system under normal conditions, BChE becomes upregulated in several neurodegenerative conditions, including Alzheimer’s disease (AD), where it is often found in higher concentrations in the brain [1]. Notably, BChE is thought to play a more significant role in the pathogenesis of AD than previously appreciated, and this has led to increased interest in selective BChE inhibitors as potential treatments. Selective inhibition of BChE using carbamates is being actively explored for its therapeutic benefits, particularly in AD. In contrast to traditional carbamates, which inhibit both AChE and BChE, selective BChE inhibitors aim to specifically target BChE while leaving AChE activity relatively intact. This selective inhibition may offer several advantages. For instance, while both AChE and BChE contribute to cholinergic dysfunction in AD, BChE is particularly important in the later stages of the disease, where its levels increase and correlate with the severity of cognitive decline [2,3]. By selectively inhibiting BChE, carbamates can help restore a more balanced cholinergic tone, potentially improving cognitive function with fewer side effects than dual inhibitors of AChE and BChE. Moreover, BChE is implicated in the aggregation of amyloid-beta peptides, a hallmark of AD pathology, and the selective inhibition of BChE may reduce amyloid-beta toxicity, providing an additional therapeutic benefit [4].

One notable example of a carbamate selective for BChE is Phenserine, which has been studied for its ability to inhibit BChE without significantly affecting AChE activity at lower doses [5]. Phenserine has shown promise in preclinical studies and clinical trials by improving cognitive function in patients with AD while reducing the risk of side effects such as nausea and diarrhea, which are common with non-selective cholinesterase inhibitors [6]. Additionally, phenserine’s selective inhibition of BChE has been linked to a reduction in amyloid-beta aggregation, an effect that has significant therapeutic implications for slowing the progression of AD. This dual action—restoring cholinergic function and reducing amyloid-beta toxicity—highlights the potential advantages of selective BChE inhibitors over more traditional, non-selective carbamates.

Other selective BChE inhibitors have also been synthesized and tested for their effects on both cholinergic transmission and amyloid-beta pathology. For example, rivastigmine, although initially developed as a dual AChE and BChE inhibitor, has been shown to have a higher affinity for BChE in certain doses, suggesting that at optimal concentrations, it may primarily target BChE in the treatment of AD [7,8]. Similarly, carbamates like donepezil have been shown to exert some degree of selective BChE inhibition under specific conditions, making them viable candidates for future exploration in diseases with pronounced BChE activity, such as AD and Parkinson’s disease [8]. These findings suggest that carbamates with selective BChE inhibitory properties may not only help alleviate symptoms of cognitive decline but may also have disease-modifying effects by interacting with the neurodegenerative processes associated with amyloid plaque formation. The role of BChE in amyloid-beta aggregation has driven much of the research into selective BChE inhibitors. In AD, amyloid plaques accumulate and disrupt normal neuronal function. Research results suggest that BChE can interact with amyloid-beta peptides, promoting their aggregation and exacerbating neurodegeneration [4]. By inhibiting BChE specifically, carbamates may reduce amyloid-beta aggregation, thus providing a dual benefit: enhancing cholinergic transmission and potentially mitigating the pathological effects of amyloid plaques.

Selective BChE inhibitors may also offer therapeutic benefits in other neurodegenerative diseases. For example, Parkinson’s disease (PD), which involves both dopaminergic and cholinergic dysfunction, may benefit from the modulation of BChE activity. In Parkinson’s disease, BChE contributes to the imbalance between the cholinergic and dopaminergic systems. The selective inhibition of BChE could potentially restore this balance, alleviating cognitive and motor symptoms [9]. Given the high prevalence of cognitive decline in PD and other conditions like Lewy body dementia, selective BChE inhibition is a promising strategy for improving both cognitive and motor function. In conclusion, selective BChE inhibition by carbamates represents a promising strategy in the treatment of neurodegenerative disorders, particularly AD, where BChE activity is upregulated and linked to disease progression. By specifically targeting BChE, these compounds may enhance cholinergic transmission, reduce amyloid-beta aggregation, and provide neuroprotective effects, all while minimizing the side effects commonly seen with non-selective cholinesterase inhibitors. Research into the design and testing of selective BChE inhibitors continues to expand the understanding of their therapeutic potential, and they may ultimately serve as a critical component of future treatments aimed at slowing the progression of neurodegenerative diseases.

**Figure 1:**
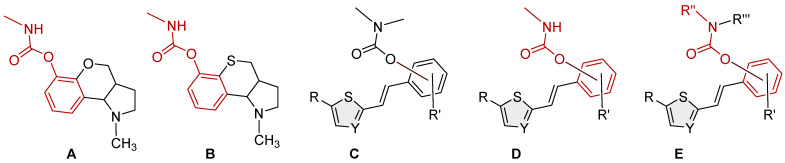
Structures of some carbamates as cholinesterase inhibitors (**A**,**B**) [16], resveratrol-based bioactive carbamates (**C**), and structures investigated in the current research (**D**,**E**).

The structures of various other carbamates as cholinesterase inhibitors are well known in the literature [10,11,12,13,14,15,16,17,18] (Figure 1, structures A and B [16]). Considering our experience in the design of new cholinesterase inhibitors, especially resveratrol analogs, the basic stilbene skeleton was used as a structural unit for carbamates in a previous study (Figure 1, structure C) [19]. Inhibitory activity was tested toward AChE and BChE enzymes. In the tested group of compounds, two leading inhibitors achieved excellent selective inhibitory activity for BChE, with IC_50_ values of 0.12 ± 0.09 μM and 0.38 ± 0.01 μM. Both were much more active than galantamine, the standard inhibitor against BChE. Molecular docking of the most promising inhibitor candidates revealed that stabilizing interactions between the active site residues of BChE and the ligands involve π-stacking and alkyl-π interactions depending on the orientation of the carbamate group.

Based on in silico, experimental, and computational results on biological activity, the previously identified heterostilbene carbamates represented potential selective resveratrol-like BChE inhibitors as new therapeutics for neurological disorders. These compounds provided an excellent initial basis for the design and synthesis of new heterostilbene carbamates (Figure 1, structures D and E) to create a larger chemical library for high-throughput screening and drug development.

## 2. Materials and Methods

### 2.1. General Remarks

NMR spectra were acquired using Bruker AV300 and AV600 spectrometers (Bruker BioSpin GmbH, Rheinstetten, Germany) equipped with a 5 mm probe head at the Ruđer Bošković Institute. Standard ^1^H and proton-decoupled ^13^C{^1^H} NMR spectra were measured at 300.000 and 600.130 MHz for ^1^H and at 75.432 and 150.903 MHz for ^13^C. Chemical shifts (*δ*, in ppm) were calibrated using the signal of tetramethylsilane (TMS). All spectra were recorded in deuterated chloroform (CDCl_3_) at 25 °C. Reactions were monitored by thin-layer chromatography (TLC) on silica gel-coated plates (0.2 mm, 60/Kieselguhr F_254_), developed in 10 mL of the appropriate solvent system. When the reactions were finished, liquid–liquid extraction between water and dichloromethane (DCM), with 2.4 M hydrochloric acid added, was performed. After separating the aqueous and organic phases, the organic layer was dried over anhydrous magnesium sulfate (MgSO_4_). The purification of products was carried out by column chromatography using glass columns of varying diameters, packed with technical-grade silica gel (60 Å) to appropriate heights. The following abbreviations are used in this section: TEA—triethylamine; DMAP—4-dimethylaminopyridine; DCE—1,2-dichloroethane; PE—petroleum ether; E—diethyl ether; HCl—hydrochloric acid; NMR—nuclear magnetic resonance. ^1^H NMR signal multiplicities are defined as follows: s—singlet; d—doublet; t—triplet; m—multiplet; dd—doublet of doublets; dt—doublet of triplets; dq—doublet of quartets. High-resolution mass spectrometry (HRMS) was conducted using a MALDI-TOF/TOF instrument equipped with an Nd:YAG laser (355 nm) operating at a repetition rate of 200 Hz.

### 2.2. Synthesis of Heterostilbene Carbamates ***1**–**19***

Initially synthesized *trans*-resveratrol analogs [20] were used as preliminary compounds in synthesizing heterostilbene carbamates **1**–**19**. Accordingly, 50–100 mg of the appropriate resveratrol analog was dissolved in 1.2 mL of DCE in a round-bottomed flask. Then, 1.8 eq of TEA was added to the reaction flask with 0.09 eq of DMAP. The reaction mixture was stirred at room temperature for 15 min, after which it was purged with argon for up to 1 min. Following this, 1.2 eq of the corresponding carbamoyl chloride was added dropwise, and the mixture was stirred at 60 °C under inert conditions for 4 h. Upon completion, the reaction mixture was extracted with 10 mL DCM and 10 mL distilled water, followed by adding 1 mL of 2.4 M HCl in the second extraction step. The organic layer was dried over anhydrous magnesium sulfate (MgSO_4_), then filtered and concentrated to dryness using a rotary evaporator. The resulting carbamates (compounds **1**–**19**) were purified by column chromatography using various ratios of petroleum ether and diethyl ether as the eluent system. NMR analysis confirmed that compounds **1**–**19** were predominantly obtained as *trans*-isomers, with minor amounts of *cis*-isomers detected in some cases. An exception was thiazole carbamate 8, which showed a higher proportion of the corresponding *cis*-isomer. 
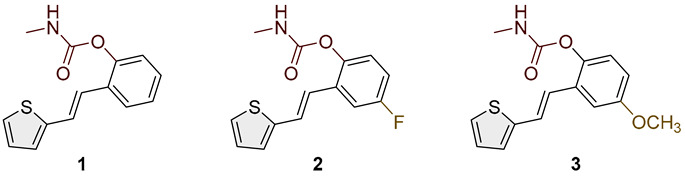

(*E*)-2-(2-(thiophen-2-yl)vinyl)phenyl methylcarbamate (**1**): 37 mg (isolated 31%), white powder; m.p. 117–119 °C; *R*_f_ (50% PE/E) = 0.50; ^1^H NMR (CDCl_3_, 300 MHz) *δ*/ppm: 7.61 (d, *J* = 7.4 Hz, 1H), 7.26 (m, 3H), 7.20 (d, *J* = 5.3 Hz, 2H), 7.13 (d, *J* = 17.5 Hz, 1H), 7.12–7.10 (m, 1H), 7.05–7.00 (m, 1H), 5.10 (br s, 1H), 2.92 (d, *J* = 4.8 Hz, 3H); ^13^C NMR (CDCl_3_, 75 MHz) *δ*/ppm: 155.0, 154.0, 148.3, 142.9, 128.3, 127.6, 126.4, 126.1, 125.8, 124.6, 123.6, 123.0, 121.8, 27.9; HRMS (ESI) (*m*/*z*) for C_14_H_13_NO_2_S: [M + H]^+^_calcd_ = 259.0667, and [M + H]^+^_measured_ = 259.0666.
(E)-4-fluoro-2-(2-(thiophen-2-yl)vinyl)phenyl methylcarbamate (**2**): 39 mg (isolated 33%), white powder; m.p. 110–111 °C; *R*_f_ (50% PE/E) = 0.50; ^1^H NMR (CDCl_3_, 600 MHz) *δ*/ppm: 7.28 (d, *J* = 9.7, 1H), 7.22 (d, *J* = 4.5 Hz, 1H), 7.18 (d, *J* = 18.4 Hz, 1H), 7.09 –7.02 (m, 2H), 7.01 (t, *J* = 2.7 Hz, 1H), 7.00–6.9 (m, 2H), 5.10 (br s, 1H), 2.92 (d, *J* = 4.2, 3H); ^13^C NMR (CDCl_3_, 150 MHz) *δ*/ppm: 160.2 (d, *J_CF_* = 243.3 Hz), 155.1, 144.3, 142.0, 130.6 (d, *J_CF_* = 7.7 Hz), 127.7, 125.1, 124.6, 124.0 (d, *J_CF_* = 8.7 Hz), 120.7, 114.9, 111.9, 27.6; HRMS (ESI) (*m*/*z*) for C_14_H_12_FNO_2_S: [M + H]^+^_calcd_ = 277.0573, and [M + H]^+^_measured_ = 277.0572.
(E)-4-methoxy-2-(2-(thiophen-2-yl)vinyl)phenyl methylcarbamate (**3**): 27 mg (isolated 23%), white powder; m.p. 123–124 °C; *R*_f_ (50% PE/E) = 0.25; ^1^H NMR (CDCl_3_, 600 MHz) *δ*/ppm: 7.33–7.21 (m, 2H), 7.20 (d, *J* = 3.1 Hz, 1H), 7.10 (d, *J* = 3.1 Hz, 1H), 7.03 (d, *J* = 8.9 Hz, 1H), 7.00 (t, *J* = 3.9 Hz, 1H), 6.91 (d, *J* = 16.3 Hz, 1H), 6.82 (dd, *J* = 2.7, 9.6 Hz, 1H), 5.00 (br s, 1H), 3.81 (s, 3H), 2.92 (d, *J* = 4.8 Hz, 3H); ^13^C NMR (CDCl_3_, 150 MHz) *δ*/ppm: 157.1, 155.4, 142.7, 142.2, 129.9, 130.7, 127.6, 126.5, 124.7, 123.8, 121.8, 114.1, 110.4, 55.7, 29.7; HRMS (ESI) (*m*/*z*) for C_15_H_15_NO_3_S: [M + H]^+^_calcd_ = 289.0772, and [M + H]^+^_measured_ = 289.0773.
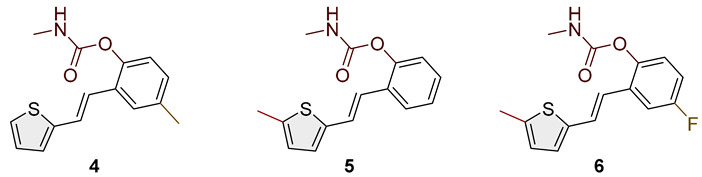
(*E*)-4-methyl-2-(2-(thiophen-2-yl)vinyl)phenyl methylcarbamate (**4**): 22 mg (isolated 18%), white powder; m.p. 109–111 °C; *R*_f_ (50% PE/E) = 0.50; ^1^H NMR (CDCl_3_, 600 MHz) *δ*/ppm: 7.41 (s, 1H), 7.22–7.17 (m, 2H), 7.09–7.04 (m, 2H), 7.03–6.96 (m, 3H), 5.04 (br s, 1H), 2.92 (d, *J* = 4.8 Hz, 3H), 2.35 (s, 3H); ^13^C NMR (CDCl_3_, 150 MHz) *δ*/ppm: 155.2, 146.2, 143.0, 135.3, 129.4, 129.1, 127.6, 126.4, 126.3, 124.5, 123.3, 122.7, 121.9, 27.9, 21.0; HRMS (ESI) (*m*/*z*) for C_15_H_15_NO_2_S: [M + H]^+^_calcd_ = 273.0824, and [M + H]^+^_measured_ = 273.0826.
(E)-2-(2-(5-methylthiophen-2-yl)vinyl)phenyl methylcarbamate (**5**): 18 mg (isolated 43%), white powder; m.p. 114–116 °C; *R*_f_ (50% PE/E) = 0.50; ^1^H NMR (CDCl_3_, 600 MHz) *δ*/ppm: 7.66–7.55 (m, 1H), 7.24–7.16 (m, 2H), 7.15–7. 09 (m, 2H), 6.89 (t, *J* = 16.7 Hz, 1H), 6.85 (d, *J* = 3.8 Hz, 1H), 6.60 (d, *J* = 3.5 Hz, 1H), 5.07 (br s, 1H), 2.93 (d, *J* = 4.6 Hz, 3H), 2.48 (s, 3H); ^13^C NMR (CDCl_3_, 150 MHz) *δ*/ppm: 155.0, 148.2, 140.9, 139.5, 130.0, 128.0, 126.8, 125.9, 125.8, 125.7, 124.0, 123.0, 120.5, 29.7, 15.6; HRMS (ESI) (*m*/*z*) for C_15_H_15_NO_2_S: [M + H]^+^_calcd_ = 273.0824, and [M + H]^+^_measured_ = 273.0825.
(E)-4-fluoro-2-(2-(5-methylthiophen-2-yl)vinyl)phenyl methylcarbamate (**6**): 19 mg (isolated 42%), white powder; m.p. 105–107 °C; *R*_f_ (50% PE/E) = 0.50; ^1^H NMR (CDCl_3_, 600 MHz) *δ*/ppm: 7.29–7.23 (m, 2H), 7.09 (d, *J* = 16.4 Hz, 2H), 6.87 (d, *J* = 3.4 Hz, 1H), 6.80 (d, *J* = 16.4 Hz, 1H), 6.66–6.64 (m, 1H), 5.06 (br s), 2.93 (d, *J* = 4.9 Hz, 3H), 2.48 (s, 3H); ^13^C NMR (CDCl_3_, 150 MHz) *δ*/ppm: 160.3 (*J_CF_* = 243.1 Hz), 155.0, 144.0, 140.3, 140.2, 131.9 (d, *J_CF_* = 8.3 Hz), 127.5, 125.9, 125.0, 124.4 (d, *J_CF_* = 8.3 Hz), 119.4, 114.7 (d, *J_CF_* = 24.1 Hz), 111.8 (d, *J_CF_* = 24.1 Hz), 29.5, 15.7; HRMS (ESI) (*m*/*z*) for C_15_H_14_FNO_2_S: [M + H]^+^_calcd_ = 291.0729, and [M + H]^+^_measured_ = 291.0731.
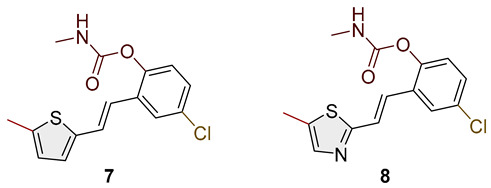
(*E*)-4-chloro-2-(2-(5-methylthiophen-2-yl)vinyl)phenyl methylcarbamate (**7**): 12 mg (isolated 25%), white powder; m.p. 122–123 °C; *R*_f_ (50% PE/E) = 0.50; ^1^H NMR (CDCl_3_, 600 MHz) *δ*/ppm: 7.55 (d, *J* = 2.5 Hz, 1H), 7.13–7.09 (m, 2H), 7.06 (d, *J* = 8.9 Hz, 1H), 6.87 (d, *J* = 3.4, 1H), 6.80–6.76 (m, 1H), 6.66–6.64 (m, 1H), 5.07 (br s), 2.93 (d, *J* = 4.9 Hz, 3H), 2.47 (s, 3H); ^13^C NMR (CDCl_3_, 150 MHz) *δ*/ppm: 154.7, 147.7, 146.5, 140.3, 131.8, 131.2, 127.7, 127.5, 125.9, 125.5, 125.2, 125.1, 124.3, 29.7, 15.7; HRMS (ESI) (*m*/*z*) for C_15_H_14_ClNO_2_S: [M + H]^+^_calcd_ = 307.0434, and [M + H]^+^_measured_ = 307.0433.
Mixture of (*E*) and (*Z*)-4-chloro-2-(2-(5-methylthiazol-2-yl)vinyl)phenyl methylcarbamate (**8**); for (*E*)-isomer, the data are the following: 8 mg (isolated 17%), *R*_f_ (50% PE/E) = 0.30; ^1^H NMR (CDCl_3_, 600 MHz) *δ*/ppm: 7.52 (s, 1H), 7.26 (s, 1H), 7.12 (d, *J* = 16.6 Hz, 1H), 7.05 (d, *J* = 8.1 Hz, 1H), 7.01 (d, *J* = 8.8 Hz, 1H), 6.80 (d, *J* = 16.3 Hz, 1H), 2.58 (d, *J* = 5.0 Hz, 3H), 2.57 (s, 3H); MS (ESI) (*m*/*z*) for C_17_H_19_NO_3_S: [M + H]^+^ 308 (100).
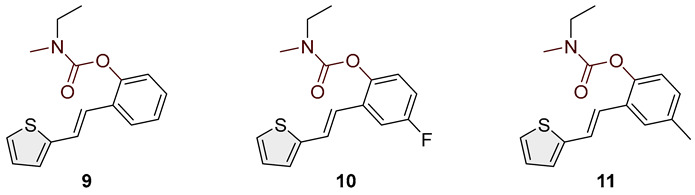
(*E*)-2-(2-(thiophen-2-yl)vinyl)phenyl ethyl(methyl)carbamate (**9**): 40 mg (isolated 75%), colorless oil; *R*_f_ (50% PE/E) = 0.80; ^1^H NMR (CDCl_3_, 600 MHz) *δ*/ppm: 7.63–7.58 (m, 1H), 7.38–7.31 (m, 1H), 7.27–7.23 (m, 1H), 7.22–7.17 (m, 3H), 7.13 (t, *J* = 7.15 Hz, 1H), 7.05 (d, *J* = 3.3 Hz, 1H), 7.00–6.98 (m, 1H), 3.50 (dq, *J* = 7.3, 80.6 Hz, 2H), 3.10 (d, *J* = 87.6 Hz, 3H), 1.28–1.22 (m, 3H); ^13^C NMR (CDCl_3_, 150 MHz) *δ*/ppm: 154.3, 148.8, 143.1, 130.4, 129.7, 128.8, 128.3, 127.6, 126.3, 125.6, 124.5, 123.4, 122.8, 44.1, 21.0, 13.5; HRMS (ESI) (*m*/*z*) for C_16_H_17_NO_2_S: [M + H]^+^_calcd_ = 287.0980, and [M + H]^+^_measured_ = 287.0985.
(E)-4-fluoro-2-(2-(thiophen-2-yl)vinyl)phenyl ethyl(methyl)carbamate (**10**): 28 mg (isolated 53%), colorless oil; *R*_f_ (50% PE/E) = 0.50; ^1^H NMR (CDCl_3_, 300 MHz) *δ*/ppm: 7.33–7.27 (m, 1H), 7.22 (d, *J* = 3.89 Hz, 1H), 7.17–7.04 (m, 3H), 7.03–6.86 (m, 3H), 3.49 (dq, *J* = 7.3, 39.1 Hz, 2H), 3.09 (d, *J* = 43.3 Hz, 3H), 1.25–1.20. (m, 3H); ^13^C NMR (CDCl_3_, 75 MHz) *δ*/ppm: 160.1 (d, *J_CF_* = 242.6 Hz), 158.5, 154.1, 144.7, 142.5, 127.7, 127.1, 126.5, 126.3, 125.0 (d, *J_CF_* = 8.7 Hz), 120.8 (d, *J_CF_* = 8.7 Hz), 115.1 (d, *J_CF_* = 24.1 Hz), 111.8 (d, *J_CF_* = 24.1 Hz), 44.1, 34.5, 14.2.
(E)-4-methyl-2-(2-(thiophen-2-yl)vinyl)phenyl ethyl(methyl)carbamate (**11**): 85 mg (isolated 91%), colorless oil; *R*_f_ (50% PE/E) = 0.80; ^1^H NMR (CDCl_3_, 600 MHz) *δ*/ppm: 7.41 (s, 1H), 7.21–7.15 (m, 2H), 7.10–6.94 (m, 5H), 3.49 (dq, *J* = 7.0, 79.1 Hz, 2H), 3.08 (d, *J* = 86.4 Hz, 3H), 2.34 (s, 3H), 1.33–1.28 (m, 3H); ^13^C NMR (CDCl_3_, 150 MHz) *δ*/ppm: too small a quantity for recording; HRMS (ESI) (*m*/*z*) for C_17_H_19_NO_2_S: [M + H]^+^_calcd_ = 301.1136, and [M + H]^+^_measured_ = 301.1141.
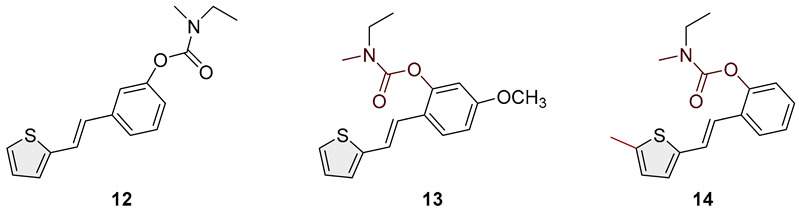
(*E*)-3-(2-(thiophen-2-yl)vinyl)phenyl ethyl(methyl)carbamate (**12**): 63 mg (isolated 87%), colorless oil; *R*_f_ (50% PE/E) = 0.80; ^1^H NMR (CDCl_3_, 600 MHz) *δ*/ppm: 7.31 (t, *J* = 7.7 Hz, 1H), 7.28–7.22 (m, 3H), 7.20 (d, *J* = 4.5 Hz, 1H), 7.06 (d, *J* = 1.8 Hz, 1H), 7.03–6.97 (m, 2H), 6.90 (d, *J* = 15.8 Hz, 1H), 3.46 (dq, *J* = 7.2, 39.8 Hz, 2H), 3.04 (d, *J* = 45.6 Hz, 3H), 1.26 (t, *J* = 7.2 Hz, 3H); ^13^C NMR (CDCl_3_, 75 MHz) *δ*/ppm: 154.5, 151.9, 142.6, 138.4, 129.4, 128.3, 127.6, 126.3, 124.5, 123.3, 122.5, 120.9, 119.2, 44.1, 34.3, 13.2; HRMS (ESI) (*m*/*z*) for C_16_H_17_NO_2_S: [M + H]^+^_calcd_ = 287.0980, and [M + H]^+^_measured_ = 287.0982.
(E)-5-methoxy-2-(2-(thiophen-2-yl)vinyl)phenyl ethyl(methyl)carbamate (**13**): 18 mg (isolated 53%), colorless oil; *R*_f_ (50% PE/E) = 0.50; ^1^H NMR (CDCl_3_, 300 MHz) *δ*/ppm: 7.52 (d, *J* = 8.9 Hz, 1H), 7.16–7.03 (m, 2H), 7.02–6.84 (m, 3H), 6.80–6.66 (m, 2H), 3.81 (s, 3H), 3.50 (dq, *J* = 7.2, 37.7 Hz, 2H), 3.09 (d, *J* = 42.4 Hz, 3H), 1.26–1.20 (m, 3H); ^13^C NMR (CDCl_3_, 75 MHz) *δ*/ppm: 160.1, 159.8, 149.6, 143.4, 127.6, 126.4, 125.7, 123.8, 122.4, 121.7, 121.3, 112.4, 108.3, 55.5, 44.2, 29.7, 13.5; MS (ESI) (*m*/*z*) for C_17_H_19_NO_3_S: [M + H]^+^ 318 (100).
(E)-2-(2-(5-methylthiophen-2-yl)vinyl)phenyl ethyl(methyl)carbamate (**14**): 25 mg (isolated 23%), colorless oil; *R*_f_ (50% PE/E) = 0.80; ^1^H NMR (CDCl_3_, 600 MHz) *δ*/ppm: 7.58–7.50 (m, 1H), 7.16–7.11 (m, 1H), 7.05 (dt, *J* = 8.5; 2.1 Hz, 1H), 7.02–6.93 (m, 2H), 6.75 (d, *J* = 15.0 Hz, 1H), 6.39 (dd, *J* = 15.0; 1.8 Hz, 1H), 3.49 (dq, *J* = 7.9, 72.5 Hz, 2H), 3.05–2.84 (m, 3H), 2.60–2.32 (m, 3H), 1.24–1.18 (m, 3H); ^13^C NMR (CDCl_3_, 150 MHz) *δ*/ppm: too small a quantity for recording; MS (ESI) (*m*/*z*) for C_17_H_19_NO_2_S: [M + H]^+^ 302 (100).
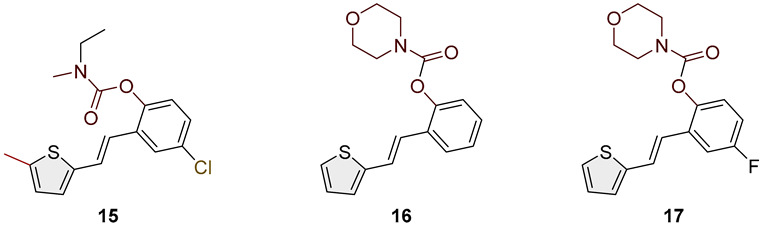
(*E*)-4-chloro-2-(2-(5-methylthiophen-2-yl)vinyl)phenyl ethyl(methyl)carbamate (**15**): 8 mg (isolated 13%), colorless oil; *R*_f_ (50% PE/E) = 0.80; ^1^H NMR (CDCl_3_, 300 MHz) *δ*/ppm: ^1^H NMR (CDCl_3_, 600 MHz) δ/ppm: 7.20–7.14 (m, 2H), 7.07–6.96 (m, 5H), 3.50 (dq, *J* = 7.0, 79.1 Hz, 2H), 3.09 (d, *J* = 86.4 Hz, 3H), 2.35 (s, 3H), 1.31–1.27 (m, 3H); ^13^C NMR (CDCl_3_, 75 MHz) *δ*/ppm: too small a quantity for recording; HRMS (ESI) (*m*/*z*) for C_17_H_18_ClNO_2_S: [M + H]^+^_calcd_ = 335.0747, and [M + H]^+^_measured_ = 335.0744.
(E)-2-(2-(thiophen-2-yl)vinyl)phenyl morpholine-4-carboxylate (**16**): 75 mg (isolated 90%), colorless oil; *R*_f_ (50% PE/E) = 0.50; ^1^H NMR (CDCl_3_, 300 MHz) *δ*/ppm: 7.62 (dd, *J* = 1.7, 7.5 Hz, 1H) 7.21–7.09 (m, 4H), 7.03–6.84 (m, 4H), 3.88–3. 44 (m, 8H); ^13^C NMR (CDCl_3_, 75 MHz) *δ*/ppm: 153.4, 148.5, 142.9, 139.6, 130.4, 128.4, 127.7, 126.7, 125.9, 124.6, 123.1, 121.6, 66.8, 66.6 (one quaternary C is missing); HRMS (ESI) (*m*/*z*) for C_17_H_17_NO_3_S: [M + H]^+^_calcd_ = 315.0929, and [M + H]^+^_measured_ = 315.0932.
(E)-4-fluoro-2-(2-(thiophen-2-yl)vinyl)phenyl morpholine-4-carboxylate (**17**): 40 mg (isolated 71%), colorless oil; *R*_f_ (50% PE/E) = 0.50; ^1^H NMR (CDCl3, 300 MHz) *δ*/ppm: 7.33–7.20 (m, 3H), 7.16–6.92 (m, 4H), 6.88 (d, *J* = 15.5 Hz, 1H), 3.88–3.43 (m, 8H); ^13^C NMR (CDCl_3_, 75 MHz) *δ*/ppm: 160.3 (d, *J* = 244.3 Hz), 153.3, 144.3, 142.3, 131.4 (d, *J* = 8.5 Hz), 127.8, 127.3, 126.5 (d, *J* = 8.5 Hz), 125.1, 124.8, 120.5, 115.0 (d, *J* = 23.9 Hz), 111.9 (d, *J* = 23.9 Hz), 66.7, 66.6; HRMS (ESI) (*m*/*z*) for C_17_H_16_FNO_3_S: [M + H]^+^_calcd_ = 333.0835, and [M + H]^+^_measured_ = 333.0838.
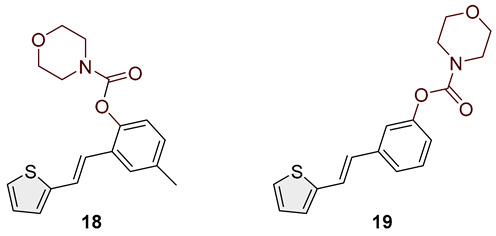
(*E*)-4-methyl-2-(2-(thiophen-2-yl)vinyl)phenyl morpholine-4-carboxylate (**18**): 78 mg (isolated 88%), colorless oil; *R*_f_ (50% PE/E) = 0.50; ^1^H NMR (CDCl_3_, 300 MHz) *δ*/ppm: 7.41 (s, 1H), 7.26–7.14 (m, 2H), 7.11–7.00 (m, 4H), 6.93 (d, *J* = 16.3 Hz, 1H); ^13^C NMR (CDCl_3_, 75 MHz) *δ*/ppm: 153.6, 146.4, 143.0, 135.4, 129.2, 127.7, 126.5, 126.4, 124.5, 123.4, 122.7, 121.7, 66.7, 66.5, 21.0 (one quaternary C is missing); HRMS (ESI) (*m*/*z*) for C_18_H_19_NO_3_S: [M + H]^+^_calcd_ = 329.1086, and [M + H]^+^_measured_ = 329.1091.
(E)-3-(2-(thiophen-2-yl)vinyl)phenyl morpholine-4-carboxylate (**19**): 87 mg (isolated 93%), colorless oil; *R*_f_ (50% PE/E) = 0.50; ^1^H NMR (CDCl_3_, 300 MHz) *δ*/ppm: 7.38–7.28 (m, 2H), 7.25–7.18 (m, 3H), 7.14–6.95 (m, 3H), 6.89 (d, *J* = 16.0 Hz, 1H), 3.80–3.50 (m, 8H) ^13^C NMR (CDCl_3_, 75 MHz) *δ*/ppm: 153.6, 151.6, 142.6, 138.6, 129.5, 127.6, 127.4, 126.4, 124.6, 120.7, 119.1, 66.6, 66.5 (two quaternary C are missing); HRMS (ESI) (*m*/*z*) for C_22_H_23_NO_3_S: [M + H]^+^_calcd_ = 315.0929, and [M + H]^+^_measured_ = 315.0934.

### 2.3. X-Ray Crystallography

Single-crystal measurements were performed on an XtaLAB Synergy diffractometer with a HyPix600 detector, using micro-focus sealed X-ray tube with CuKα (1.54184 Å) radiation at 100 K for compound **1** and room temperature [293(2) K] for compound **4**. The CrysAlisPRO package1.171.43.136a [21] was used for data collection and reduction. The crystal structures were solved and refined within the OLEX2 program [22] using SHELXT and SHELXL [23]. The model was refined using full matrix least squares refinement; all non-hydrogen atoms were refined anisotropically. Hydrogen atoms were modeled as riding entities using the AFIX command. Molecular geometry calculations were performed by PLATON [24], and molecular graphics were prepared using CCDC-Mercury [25]. Experimental details on crystal data, data collection, and structure refinement are summarized in Table 1.

CCDC-2443931 and CCDC-2441980 contain the supplementary crystallographic data for this paper. These data can be obtained free of charge via https://www.ccdc.cam.ac.uk/structures/ (accessed on 21 April 2025), by emailing data_request@ccdc.cam.ac.uk, or by contacting The Cambridge Crystallographic Data Centre, 12 Union Road, Cambridge CB2 1EZ, UK; fax: +44-1223-336033.

### 2.4. In Vitro ChE Activity Assay

The inhibitory activity of test compounds against acetylcholinesterase (AChE) and butyrylcholinesterase (BChE) was evaluated using a modified Ellman’s spectrophotometric assay [26]. AChE (EC 3.1.1.7, *Electrophorus electricus*, Type V-S), BChE (EC 3.1.1.8, equine serum), and Trisma base (2-amino-2-(hydroxymethyl)-1,3-propanediol) were obtained from Sigma-Aldrich (St. Louis, MO, USA). Substrates acetylthiocholine iodide (ATChI) and *S*-butyrylthiocholine iodide (BTChI) were also bought from Sigma-Aldrich, while Ellman’s reagent 5,5-dithiobis-(2-nitrobenzoic acid) (DTNB) was sourced from Zwijndrecht (Antwerpen, Belgium). Tested compounds were solubilized in ethanol, and 10 µL of each solution was mixed with 180 µL of Tris buffer (50 mM, pH 8.0) and 10 µL of enzyme solution (final concentration 0.03 U/mL). After adding 10 µL of DTNB (final concentration 0.3 mM), the enzymatic reaction was started by adding 10 µL of ATChI/BTChI (final concentration of 0.5 mM). To account for non-enzymatic hydrolysis, blank samples were prepared by replacing the enzyme with an equal amount (10 µL) of buffer solution. Absorbance of the reaction mixture was measured at 405 nm for 5 min at room temperature using a 96-well microplate reader (BioTek 800TSUV Absorbance Reader, Agilent, Santa Clara, CA, USA). Enzymatic activity in the absence of inhibitors was considered 100%, and all measurements were performed in triplicate. The percentage of inhibition was calculated using the following formula: Inhibition (%) = [(*A*_C_ − *A*_T_)/*A*_C_] × 100, where A_C_ is the enzyme activity without the test sample, and *A*_T_ is the enzyme activity in the presence of the test compound, calculated as mean values ± standard deviation. IC_50_ values were obtained through nonlinear regression analysis of log-transformed inhibitor concentrations versus enzyme activity.

### 2.5. Anti-Inflammatory Activity

The effect of compounds on lipopolysaccharide (LPS)-stimulated tumor necrosis factor alpha (TNFα) production was evaluated as described previously [27]. In short, peripheral blood mononuclear cells (PBMCs) were isolated from buffy coats obtained from healthy adult volunteers and resuspended in RPMI1640 medium (Capricorn Scientific, Ebsdorfergrund, Germany) supplemented with 10% heat-inactivated FBS (Biowest, Bradenton, FL, USA), 1% GlutaMAX (Gibco, Waltham, MA, USA), and 1% antibiotic–antimycotic (Gibco). Then, 2 × 10^5^ PBMCs were seeded per well of a 96-well plate. Test compounds were dissolved in dimethyl sulfoxide (DMSO, Sigma, Kawasaki, Japan), and threefold serial dilutions in DMSO were prepared and added to cells, with a starting concentration of 100 µM. After 1 h pre-incubation with test compounds, cells were stimulated with 1 ng/mL LPS from *E. coli* 0111:B4 (Sigma). Cells were incubated for 24 h at 37 °C, 5% CO_2_, followed by the collection of supernatants for the measurement of TNF-α and cell viability assessment. To evaluate cell viability, CellTiter-Glo reagent (Promega, Madison, WI, USA) was used according to the manufacturer’s instructions, and signals obtained in compound-treated wells were compared with signals in LPS-stimulated vehicle-treated samples.

TNFα concentration in supernatants was measured by ELISA using antibodies and recombinant human TNFα protein (standard) from R&D Systems. Lumitrac 600 384-well plates (Greiner Bio-One, Singapore) were coated with 1 µg/mL of TNFα capture antibody diluted in phosphate-buffered saline (PBS; Gibco) overnight at 4 °C. The following day, plates were blocked with 5% sucrose (Kemika, Singapore) in assay diluent (1% bovine serum albumin (BSA; Sigma) in PBS) for 4 h at RT. After the blocking step, samples and standards were added to the plates and incubated overnight at 4 °C. Afterwards, 250 ng/mL of TNFα detection antibody was added to wells, followed by a 2 h incubation at RT. Finally, after the plates were incubated with streptavidin-HRP (Invitrogen, Waltham, MA, USA), chemiluminescence ELISA Substrate (Roche, Basel, Switzerland) was added to the wells, and luminescence was measured using an EnVision 2105 multilabel reader (Revvity, Waltham, MA, USA). Measured luminescence was used to calculate concentrations of TNFα in the supernatants by interpolation from the standard curve. Percentages of inhibition were calculated from obtained cytokine concentrations, and IC_50_ values were determined using GraphPad Prism v9 software with a nonlinear regression curve fit (four parameters with variable slope).

### 2.6. Computational Details

The preparation of ligands for the docking study included the optimization of their geometries at the M06-2X/6-31 + G(d,p) level of theory using the Gaussian16 program [28]. Molecular docking was conducted using the Autodock 4.2 program package [29], with crystal structures of BChE taken from the Protein Data Bank (PDB code 7AIY) [30]. Molecular docking was performed using the Lamarckian Genetic Algorithm, with 25 genetic algorithm dockings generating 25 binding poses for ligands, while the residues of the enzyme were kept rigid. Complexes obtained by docking were used as starting structures for molecular dynamics simulations. A truncated octahedron of the OPC water box was used for solvation of the enzyme–ligand complexes. Neutralization with Cl^−^ ions using the Amber16 program package was performed [31], with the ff14SB force field [32] for the protein part of the enzyme and the General Amber Force Field (GAFF) [33] for ligands. Partial charges for ligands were derived using the RESP procedure. Equilibrations of all four systems consisted of energy minimizations and short MD simulations of 20 ns, with systematic decreases in the harmonic restraints to zero and relaxation of the volume and temperature, with target values of the temperature and pressure set to 300 K and 1 atm, respectively. Productive MD simulation with no constraints was performed for 40 nanoseconds under NPT conditions, i.e., 300 K and 1 atm.

### 2.7. ADME-Tox Predictions

pkCSM is a free-to-use machine learning platform that predicts small-molecule pharmacokinetic properties using graph-based signatures. It includes 28 models covering key ADMET (absorption, distribution, metabolism, excretion, and toxicity) properties. These include factors like permeability, solubility, absorption, distribution in the body, interactions with metabolic enzymes, excretion, and various toxicity measures. Swiss AMDE allows for the free computation of physico-chemical descriptors as well as the prediction of the ADME parameters, pharmacokinetic properties, drug-like nature, and medicinal chemistry friendliness of one or multiple small molecules to support drug discovery. AdmetSAR 3.0 is free-access chemical risk assessment tool [34]. ADMETLAb 3.0 provides easy access to comprehensive, accurate, and efficient prediction of ADMET profiles for chemicals. The prediction is based on a high-quality database of 0.37M entries spanning 77 endpoints and a directed message passing neural network (DMPNN) framework. For the purpose of this paper, all molecules were tested by multiple tools, and predictions of key features for the lead molecules are presented.

## 3. Results and Discussion

### 3.1. Synthesis and Characterization of New Heterostilbene Carbamates ***1**–**19***

**Scheme 1:**
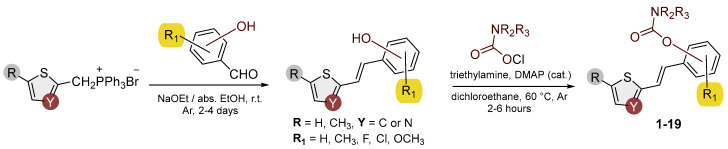
Synthetic pathway to new heterostilbene carbamates **1**–**19**.

As the starting material for the synthesis of carbamates **1***–***19**, analogs of resveratrol were used, synthesized by the Wittig reaction (Scheme 1) [20]. The Wittig reaction has been used for many years in the synthesis of conjugated systems [35,36,37,38]. New heterostilbene carbamates **1***–***19** were prepared at room temperature in dichloroethane in an inert atmosphere, with 1.8 eq of triethylamine and a catalytic amount of DMAP. After that, the reaction mixture was stirred under argon, and 1.2 eq of the corresponding carbamoyl chlorides was added. At the end, the reaction mixture was worked up (see Section 2).

**Figure 2:**
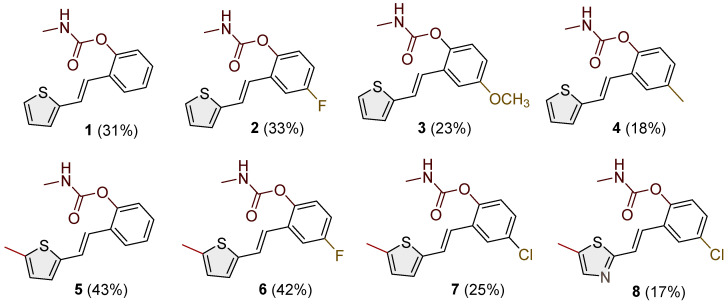
Structures and isolated yields of new methyl carbamates **1**–**8**.

**Figure 3:**
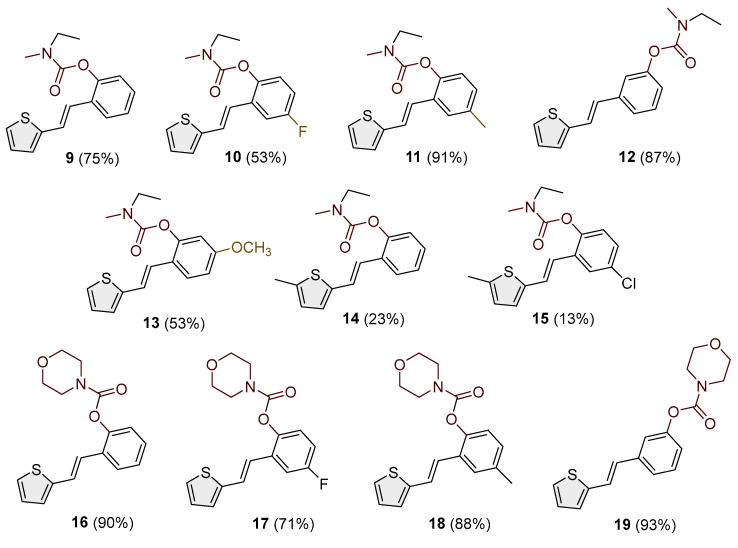
Structures and isolated yields of new ethyl-methyl carbamates **9**–**15** and morpholine carbamates **16**–**19**.

Heterostilbene carbamates **1***–***19** were purified by column chromatography using petroleum ether and diethyl ether in different ratios as the eluent and isolated with a broad range of yields, primarily depending on the carbamoyl chlorides used and on the nature and position of the substituents (13–93%, Figure 2 and Figure 3). When comparing the isolated yields of methyl carbamates **1**–**8** (Figure 2) and ethyl-methyl carbamates **9**–**19** (Figure 3), it is obvious that the isolated yields for some derivatives in the second group are very high in some cases (compounds **11**, **16**, and **19**). It can be noticed that within certain groups of carbamates (derivatives **1**–**8** or **9**–**19**), the nature of the heterocyclic nuclei, the substituent on it, and the carbamate group’s position affect each carbamate’s isolated yield.

For carbamates **9**–**19**, the highest yields were achieved with heterostilbene carbamates **11**, **12**, **16**, **18**, and **19** (Figure 3, 87–93%), which, besides the carbamoyl group, either lack additional substituents on the aromatic nucleus or contain only a methyl group, with the thiophene ring remaining unsubstituted in all cases. It should also be emphasized that all four derivatives with a morpholine carbamate group (**16**–**19**) gave high yields. The presence of a methyl substituent on the thiophene ring is not favorable for synthesis regarding the isolated yields of the carbamate products (compounds **14** and **15**).

The newly synthesized heterostilbene carbamates **1**–**19** were spectroscopically characterized, and their structures and purity were confirmed via NMR (^1^H and ^13^C) and HRMS analyses (Figures S16–S68). As in the previous investigation, new heterostilbene carbamates **1**–**19** were targeted as selective BChE inhibitors. It is worth it to mention BChE-assisted substrate (carbamate) hydrolysis again [20]. It is analogous to the mechanism of the AChE-assisted hydrolysis of ACh and includes the formation of the Michaelis complex, acylation of the enzyme, and its spontaneous deacylation with water.

### 3.2. Crystal Structures of Carbamates ***1*** and ***4***

**Figure 4:**
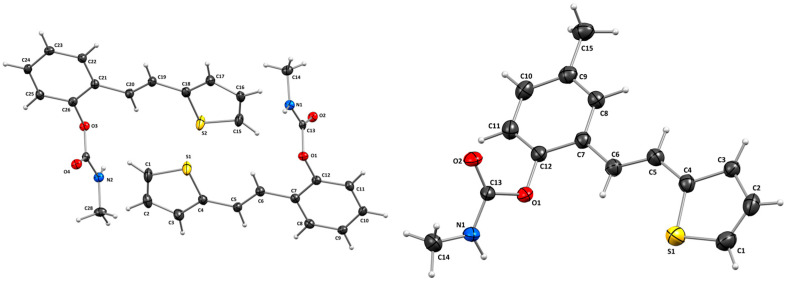
Molecular structures of compounds **1** (**left**) and **4** (**right**). Displacement ellipsoids are drawn for the probability of 30%, and hydrogen atoms are shown as spheres of arbitrary radii.

**Figure 5:**
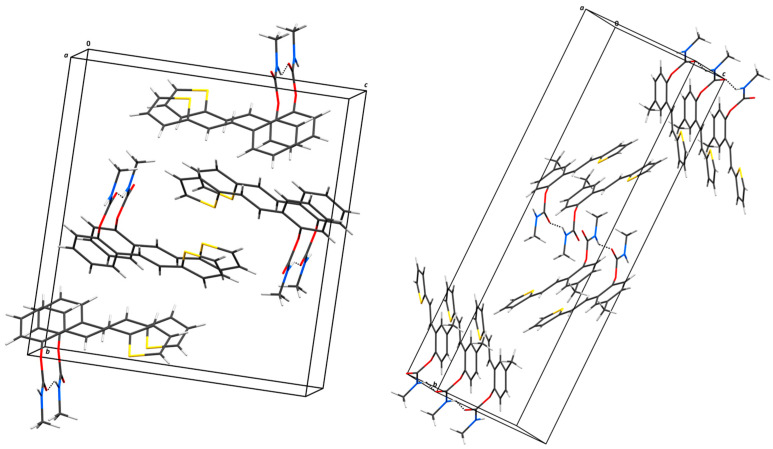
Hydrogen bonding and stacking interaction motifs in the crystal structures of **1** (**left**) and **4** (**right**). Hydrogen-bonded chains extend along the [100]-crystallographic direction. N–H···O hydrogen bonds are shown as dashed lines.

The structures of **1** and **4** were confirmed by single-crystal X-ray diffraction analysis (Figure 4). In both cases, the crystal packing is primarily governed by intermolecular N–H···O hydrogen bonds and π–π stacking interactions (Figure 5), which organize the molecules into one-dimensional chains extending along the [100]-crystallographic axis. These chains are further stabilized by weak van der Waals forces acting between adjacent layers. In the crystal structure of carbamate **1**, two symmetry-independent intermolecular hydrogen bonds, N1–H1A···O2 and N2–H2A···O4, link adjacent molecules (Table 2). The presence of multiple aromatic and heteroaromatic rings facilitates π–π stacking interactions, with interplanar distances ranging from 2.87 Å to 3.75 Å (Table 3), contributing significantly to the layered packing arrangement. In carbamate **4**, the key intermolecular interaction is the N1–H1A···O2 hydrogen bond, with geometric parameters of *d*(N–H) = 0.86 Å, *d*(H···O) = 2.14 Å, and *d*(N···O) = 2.892(3) Å, with an N–H···O angle of 145.4° and a symmetry operation of –1 + *x*, *y*, *z*. In addition to hydrogen bonding and π–π stacking, C–H···π interactions are observed in compound **4**, providing further stabilization. A representative interaction, C15–H15A···C7–C12, exhibits a C···Cg distance of 3.63 Å and occurs via the symmetry operation 1 + *x*, *y*, *z* (Table 4).

### 3.3. Cholinesterase Inhibition of Heterostilbene Carbamates ***1***–***19***

Previously tested carbamate analogs of resveratrol [20] (Figure 1, structure C) showed an interesting structural basis for developing completely selective and powerful inhibitors of BChE. Therefore, using Ellman’s modified method, a new portion of variously substituted carbamate derivatives was assayed for their inhibitory effect on AChE and BChE [26]. Results expressed as IC_50_ values of carbamate derivatives and standard galantamine are presented in Table 5 and Figure 6 and Figures S1–S15.

**Figure 6:**
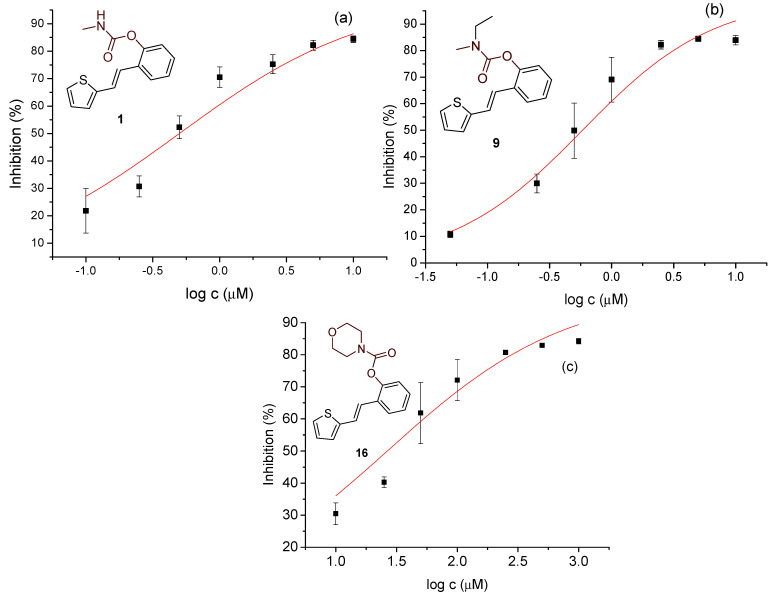
Dose–response curve for the inhibition of BChE by **1** (**a**), **9** (**b**), and **16** (**c**).

Studied compounds can be divided into three groups according to the type of carbamate part: secondary carbamates **1**–**8**, tertiary carbamates **9**–**14**, and tertiary morpholine carbamates **16**–**19**. Inside each group, variations were observed in terms of substituents present on the thiophene or benzene ring, the position of the carbamate group, and even the type of heterocycle. Only one derivative tested had a thiazole ring (derivative **8**), while all others were thiophenes. Derivative **8** was the only one to show some inhibition of AChE, albeit with a weak IC_50_ value of about 275.5 μM. Thiophene derivatives can be considered fully selective BChE inhibitors. Within the first group, the most successful inhibitor is structure **1**, with an IC_50_ value of 0.5 μM (Table 5 and Figure 6), which is in complete agreement with previous results regarding structural features, namely that resveratrol analogs benefit from the absence of a substituent on the carbamate side of the stilbene. Slightly reduced but still excellent inhibition is shown by the derivative with a methyl substituent on the thiophene, **5** (IC_50_ 0.913 μM), and that with a methyl in the *meta* position on the carbamate side, **4** (IC_50_ 1.102 μM) (Figures S3 and S4). As can be seen from Table 5, inhibitory activity decreases with the introduction of the remaining substituents, all with lone electron pairs -F, -Cl, or -OCH_3_. A similar finding was observed in the group of secondary carbamates. Again, the leading inhibitor is the one without a substituent on the carbamate side of the stilbene, derivative **9,** with an IC_50_ value of 0.583 μM, and there is almost no difference between **9** and the secondary analog **1** (Table 5 and Figure 6). Introducing a methyl group to the thiophene in derivative **14** significantly reduces the inhibition to IC_50_ 85.130 μM. In contrast, the introduction of methyl on the benzene part in structure **11** preserves excellent inhibitory activity with an IC_50_ of 1.541 μM.

Changing the position of the carbamate group from *ortho* to *meta* in derivative **12** reduces the activity threefold. However, the inhibition achieved is still excellent, with an IC_50_ value of 1.503 μM. The presence of the methoxy group in the *para* position maintains the activity in an excellent range of concentrations, and derivative **13** has an IC_50_ of 1.883 μM. The most successful inhibitors are morpholine carbamates **16**–**19**, among which structure **16** stands out as the most potent inhibitor in this group and the entire series of tested compounds. An excellent inhibition value of 26.5 nM is achieved by the derivative without additional substituents on the benzene or thiophene (Table 5 and Figure 6). The same structural feature favors inhibition, as in the previous two groups. Introducing fluorine and methyl reduces the activity, but derivatives **17** and **18** are still in an excellent range of concentrations (Table 5, Figures S13 and S14). When the carbamate group is in the *meta* position of the stilbene part in derivative **19**, the activity is reduced one hundred times; however, it still ranks among the highly active inhibitors. This group of compounds, with morpholine on the carbamate nitrogen, proved to be the most effective in inhibiting BChE, highlighting its potential for further investigation through derivative studies.

### 3.4. Anti-Inflammatory Activity of Heterostilbene Carbamates ***1**–**19***

**Figure 7:**
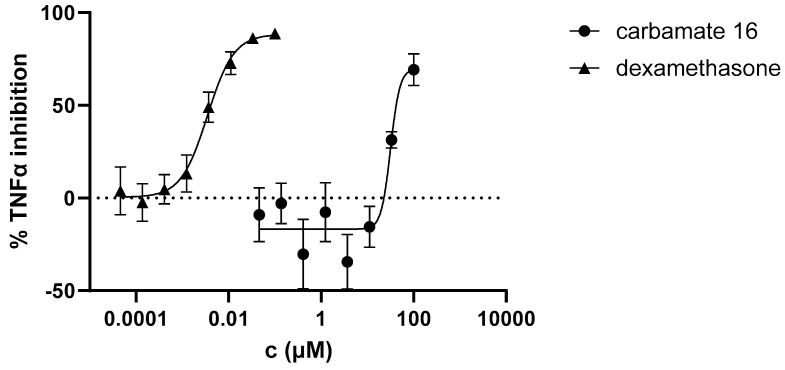
Inhibition of LPS-stimulated TNFα production in PBMCs from two donors.

The potential anti-inflammatory activity of compounds was evaluated in vitro in an assay where PBMCs were stimulated with LPS to induce TNFα production. Of all the tested compounds, only carbamate **16** inhibited LPS-stimulated TNFα production (Figure 7). The compound was active at the two highest concentrations tested, with an average IC_50_ of 31.6 µM in PBMCs from two donors. Dexamethasone, a corticosteroid widely used to treat inflammatory conditions, was used in this assay as a reference compound. As expected, it was highly effective in inhibiting LPS-stimulated TNFα production with an IC_50_ value of 3.5 nM, which aligns with results obtained previously in this assay [27].

### 3.5. ADME(T) Properties and Genotoxicity—ICH (M7) Q(SAR)

The first step in any investigation of compounds with biological activity is the study of toxicity. This will be performed before the ADMET (see below) to see which compounds should be tested further and which have structural alerts for genotoxicity. (Q)SAR models are used to predict biological activity based on structural components [39]. (Q)SAR models are especially vital during the early stages of searching for potentially active drug substances. The elimination of all compounds that have mutagenic potential saves money and time. The most commonly used tool is the Lhasa software package (Nexus v.2.5.2, Derek Nexus v.6.2.1 and Sarah Nexus v.3.2.1) because it uses two complementary models, and their predictions are then reviewed one more time by an expert. The results for compounds **1**–**19** are shown in Table 6.

From the compounds that showed biological activity (**1**, **9**, **16**, and **18**), only compound **1** was not a probable candidate for further testing, as it showed a potential for mutagenicity. If this compound was of particular interest, further in vitro AMES and even in vivo qualification studies would have to be performed to show that it can be used as an active drug. Studies related to bioavailability, that is, absorption, distribution, metabolism, excretion, and toxicity (ADME(T)), are a cornerstone of any drug development stage. They are here to give insights into the compound’s drug-like pharmacokinetic properties and whether there are any safety concerns in people [40]. These properties can be investigated in silico, in vitro, and in vivo. In the early phases of drug discovery, in silico tools provide a practical advantage when target compounds are either insufficiently pure or available only in minimal quantities. This study used free in silico tools [41,42] to screen the candidate compounds. Results are presented in Table 7.

The data presented in Table 2 show that water solubility is very low. The in silico data indicating CNS potential are the ones under distribution in Table 7.

**Figure 8:**
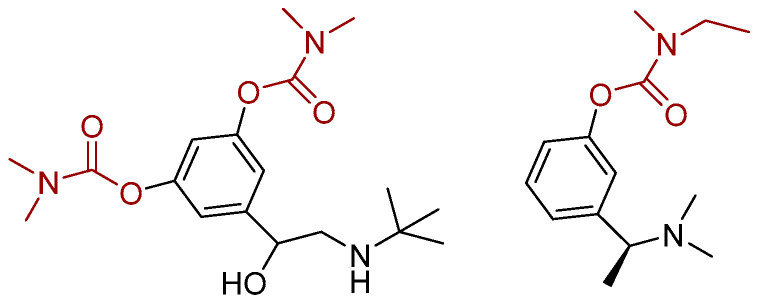
Structure of bambuterol (**left**) and rivastigmine (**right**).

The BBB (as log BB = the drug concentration in the brain divided by the concentration in the blood) for compounds **9** and **16** is approximately 0.3. This result is promising, as compounds with log BB > 0.3 readily cross the BBB. The second indicator is the log PS (permeability surface area); a log PS > −2 signifies molecules that can penetrate the CNS. This value is above −0.98 for compounds tested, so they are considered good candidates. If we consider that the unbound drug is the effective form, then a higher Fu can indicate better bioavailability, and the drug can better distribute in the central nervous system. Since these candidates are analogs containing an active carbamate moiety, the abovementioned indicators were compared with those in drugs containing a carbamate group. Bambuterol and rivastigmine (Figure 8) were selected due to structural resemblance and similar therapeutic indications as tested compounds.

As these are well-known drugs, the in silico prediction was performed only for VDss, BBB, and PS distribution parameters. The parameter VDss is similar to those for compounds **6**, **9**, and **18**. Log BB is bigger for the known drugs, but as drugs with values above 0.3 readily cross the BBB barrier, the lead compounds **6** and **9** meet the criteria. Based on the range of in silico ADME(T) parameters and genotoxicity assessments, all three compounds appear to be promising leads for further drug development.

### 3.6. Computational Study

The measurements of inhibitory activity toward cholinesterases (Table 5) show that compound **16**, one of the carbamates with a morpholine substituent, stands out as the most promising BChE inhibitor. Among the methyl and ethyl carbamates, methyl carbamate **1** exhibited the highest inhibitory activity. We performed molecular docking studies for these two compounds to gain insight into their enzyme–ligand structures and to characterize stabilizing interactions between the ligands and residues within the enzyme’s active site.

**Figure 9:**
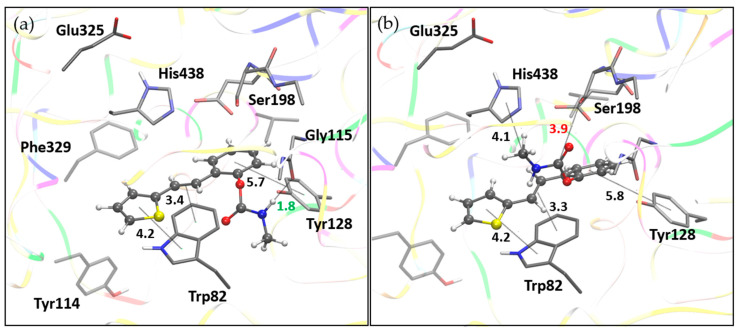
Molecule **1** docked into the active site of BChE. (**a**) Structure of the most stable complex, BChE-**1**(I), with the hydrogen bond length indicated in green. (**b**) Structure BChE-1(II) with the ligand pose from the most populated cluster; distance to serine hydroxyl oxygen marked in red. Docked molecules are presented using a ball-and-stick model, with distances given in angstroms.

Docking of molecule **1** resulted in two relevant complexes, as presented in Figure 9. The first one, labeled BChE-**1**(I), contains the ligand in a conformation that results in the most stable complex structure. The second structure is obtained with the representative ligand pose belonging to the most populated cluster (Table S1). In these two complexes, the ligand orientations are similar, with the thiophene and the double CC bond engaging in parallel π–π stacking interactions with Trp82 in the anionic subdomain of the BChE active site, while the phenyl ring of the ligand interacts with Tyr128. However, in the BChE-**1**(I) complex, a hydrogen bond is observed between the amide group of the carbamate and the nitrogen atom of one of the glycine residues in the oxyanion hole (Gly115). In the BChE-**1**(II) structure, this interaction disappears due to the rotation of the carbamate group. This conformational change allows the carbamate to approach the esteratic subdomain, where an alkyl–π interaction occurs between the ligand’s methyl group and His438, and the carbonyl carbon of the ligand comes within 3.9 Å of the oxygen atom of Ser198. However, this orientation of the carbamate group is not favorable, as the sp^2^ oxygen of the ligand’s carbonyl is positioned too close to the serine hydroxyl group.

**Figure 10:**
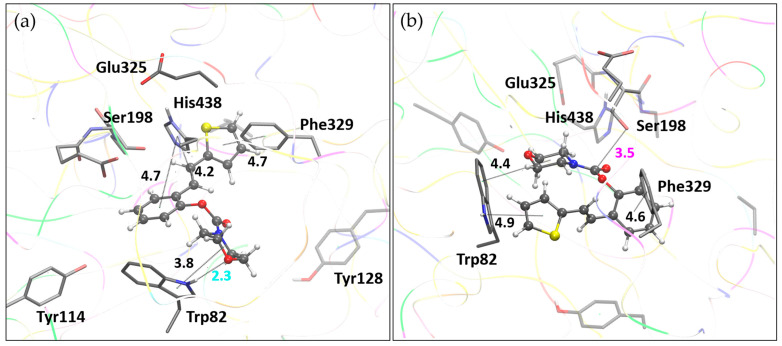
Molecule **16** docked into the active site of BChE. (**a**) Structure of the most stable complex, BChE-**16**(I), with the hydrogen bond length marked in cyan. (**b**) Structure BChE-1(II) with the ligand pose from the most populated cluster; distance to serine hydroxyl oxygen marked in magenta. Docked molecules are presented using a ball-and-stick model. Distances given in angstroms.

Analogously to methyl carbamate **1**, molecular docking of molecule **16** resulted in two pertinent enzyme–ligand complexes, both shown in Figure 10. BChE-**16**(I) represents the most stable complex, while BChE-**16**(II) corresponds to the complex containing the representative ligand pose from the most populated docking cluster.

In complex BChE-**16**(I), the hydrophobic region of the morpholine fragment engages in alkyl–π interactions with Trp82. At the same time, the oxygen of the morpholine ring forms a hydrogen bond with the polar hydrogen of the pyrrole moiety of Trp82. The thiophene ring of the ligand participates in T-shaped π–π stacking with Phe329, while the phenyl ring and double CC bond engage in π–π stacking with His438. The ligand orientation in complex BChE-**16**(II) differs, with the thiophene and morpholine interacting with Trp82, while the phenyl ring of the ligand interacts with Phe329. Most notably, the orientation of the carbamate group in this structure allows for favorable placement of the carbonyl carbon in proximity to the catalytic serine oxygen.

It is noteworthy that the binding free energy of compound **16**, estimated by molecular docking, is −5.44 kcal·mol⁻^1^, which is 0.24 kcal·mol⁻^1^ lower than that of the reference compound galantamine, a known cholinesterase inhibitor (Table S1). This result is encouraging, but the biological relevance of this difference should be validated through further experimental studies, such as cellular activity assays, to determine whether it translates into meaningful therapeutic efficacy.

**Figure 11:**
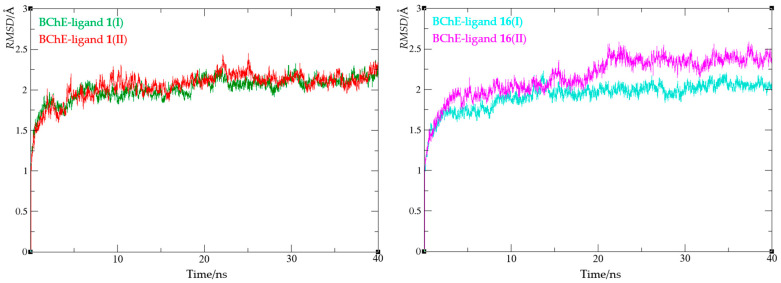
Root mean square deviation values from molecular dynamics simulations of enzyme–ligand complexes of BChE with compound **1** (**left**) and compound **16** (**right**).

To evaluate the structural stability of the enzyme–ligand complexes presented above, we performed molecular dynamics (MD) simulations, with a duration of the production simulation of 40 ns. The analysis of MD trajectories included calculating the root mean square deviation values (RMSD) for all atoms, except for hydrogens. RMSD values reflect structural changes during the simulation; all four complexes are presented in Figure 11. For both complexes with ligand **1**, the convergence was achieved after 10–15 ns, with average RMSD values of 2.02 (ranging from 0.78 to 2.37) and 2.05 (from 0.80 to 2.45 Å) for BChE-**1**(I) and BChE-**1**(II), respectively (Table S2). Complexes with morpholine carbamate **16**, I and II, behaved similarly, with average RMSD values of 1.93 and 2.17 Å, respectively. For complex BChE-**16**(I), the convergence was achieved after 10 ns; however, for complex BChE-**16**(II), the stabilization of RMSD occurred in the second half of the simulation.

**Figure 12:**
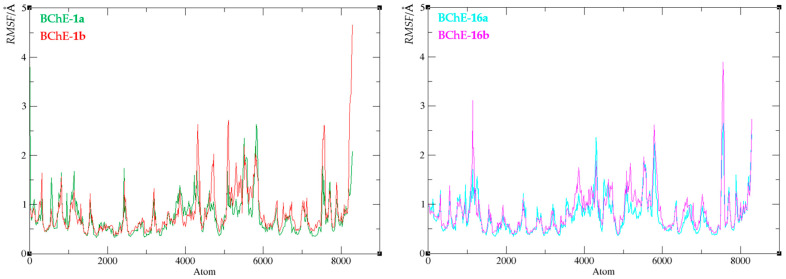
Root mean square fluctuation values of the enzyme–ligand complexes of BChE with compound **1** (**left**) and compound **16** (**right**).

Further analysis of the molecular dynamics trajectories involved calculating root mean square fluctuation (RMSF) values. RMSF quantifies the average positional deviations of the α-carbon atoms from their mean positions throughout the simulation, effectively reflecting the extent of α-carbon mobility over time. Thus, they offer insight into the dynamic behavior of the protein backbone and help identify its most mobile protein regions. The results, depicted in Figure 12 and Table S2, show that the average RMSF for all four complexes is between 0.8 and 0.9 Å, indicating low overall backbone mobility. The highest RMSF value is observed in complex BChE-**1**(II), reaching 4.65 Å and corresponding to residue Val529. The second highest value refers to Asn485 in complex BChE-**16**(I), with an RMSF of 3.89 Å. Both these residues are located far away from the active site (>25 Å), and thus their mobility does not affect the structural stability.

**Figure 13:**
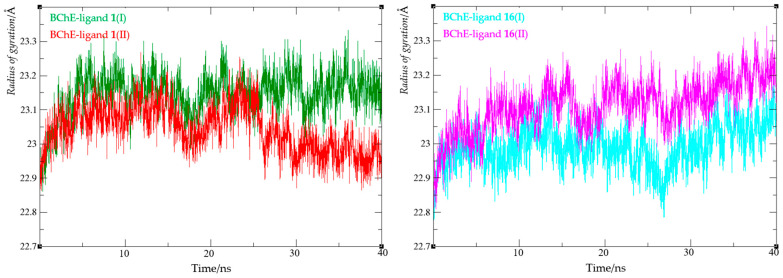
Values of the radius of gyration from molecular dynamics simulation of enzyme-ligand complexes of BChE with compounds **1** (**left**) and **16** (**right**).

Finally, the compactness of the protein upon ligand binding was evaluated by calculating the radius of gyration (Rg). The Rg value reflects the root mean square distance of all atoms from the complex’s center of mass and indicates protein flexibility in the presence of a ligand. A narrow Rg range suggests that ligand binding does not substantially disrupt the enzyme’s structural compactness. The radius of gyration values for all four complexes are summarized in Figure 13, with detailed data provided in Table S2.

For both complexes with ligand **1**, Rg values ranged from 22.86 to 23.27 Å, while for complexes with ligand **16**, they ranged from 22.81 to 23.34 Å. These findings indicate that changes in the radius of gyration were minimal across all complexes, supporting the conclusion that the enzyme retained its compact structure upon ligand binding.

## 4. Conclusions

In this study, a new series of heterostilbene carbamates was successfully synthesized and characterized as selective butyrylcholinesterase (BChE) inhibitors. Through the systematic modification of the stilbene scaffold and careful selection of substituents, 19 novel compounds were obtained, displaying defined structures confirmed by NMR, HRMS, and X-ray crystallography. The biological evaluation demonstrated that most compounds selectively inhibited BChE over acetylcholinesterase (AChE), with several derivatives, particularly compound **16**, showing outstanding potency (IC_50_ = 26.5 nM).

The selective inhibition profile aligns with the therapeutic strategy to preserve cholinergic function while minimizing side effects associated with dual cholinesterase inhibition. In addition to BChE inhibition, compound **16** exhibited moderate anti-inflammatory activity by reducing lipopolysaccharide (LPS)-induced TNF-α production. Although the potency was several logs lower in comparison to the standard anti-inflammatory compound dexamethasone, this finding supports its potential as a starting point for a multifunctional therapeutic candidate for neurodegenerative diseases. Computational studies, including molecular docking and molecular dynamics simulations, confirmed favorable binding modes and stable interactions between the carbamate derivatives and the active site of BChE.

Furthermore, in silico ADME(T) profiling indicated good predicted absorption, distribution, and low mutagenic potential for the leading compounds, strengthening their suitability for future pharmacological development. Overall, these findings highlight heterostilbene carbamates as promising scaffolds for the design of new therapeutic agents targeting cholinergic dysfunction and neuroinflammation in Alzheimer’s disease and other neurodegenerative conditions. Future work will focus on the optimization of lead compounds through further structural modifications and the exploration of additional biological activities relevant to neuroprotection.

## Figures and Tables

**Table 1:** Crystallographic data and structure refinement details for carbamates **1** and **4**.

Compound	1	4
Empirical formula	C_14_H_13_NO_2_S	C_15_H_15_NO_2_S
Formula wt./g mol^−1^	259.31	273.34
Crystal dimensions/mm	0.3 × 0.03 × 0.02	0.1 × 0.05 × 0.02
Space group	*P*2_1_	*P*2_1_/*c*
*a*/Å	5.07910(10)	5.12806(17)
*b*/Å	16.6438(2)	30.1746(7)
*c*/Å	15.4486(2)	9.2923(4)
*α*/°	90	90
*β*/°	98.0250(10)	105.137(4)
*γ*/°	90	90
Z	4	4
*V*/Å^3^	1293.17(3)	1387.97(8)
*D*_calc_/g cm^−3^	1.332	1.308
*μ*/mm^−1^	2.171	2.049
*Θ* range/°	3.93–80.03	5.12–75.85
*T*(K)	104(6)	293(2)
Radiation wavelength	1.54184 (Cu*K_α_*)	1.54184 (Cu*K_α_*)
Diffractometer type	XtaLAB Synergy, Dualflex, HyPix	XtaLAB Synergy, Dualflex, HyPix
Range of *h*, *k*, *l*	−6 > *h* > 6; −21 > *k* > 21; −19 > *l* > 19	−6 > *h* > 6; −26 > *k* > 37; −11 > *l* > 11
Reflections collected	17951	25937
Independent reflections	5485	2990
Observed reflections (*I* ≥ 2*σ*)	5146	2337
*R_int_*	0.0776	0.0899
*R (F)*	0.0508	0.0743
*R_w_ (F* ^2^ *)*	0.1369	0.2130
No. of parameters, restraints	335, 1	181, 0
Goodness of fit	1.039	1.054
Δ*ρ*_max_, Δ*ρ*_min_ (eÅ^−3^)	0.48; −0.64	0.66; −0.51

**Table 2:** Geometric parameters of hydrogen bonds determined from geometric analysis.

	*D*–H/Å	H···*A*/Å	*D*···*A*/Å	*D*–H···*A*/º	Symm. op. on *A*
**Compound 1**					
N1–H1A∙∙∙O2	0.84	2.11	2.824(4)	143	−1 + *x*, *y*, *z*
N2–H2A∙∙∙O4	0.93	2.10	2.821(4)	134	1 + *x*, *y*, *z*
C6–H6∙∙∙S1	0.95	2.73	3.156(5)	108	*x*, *y*, *z*
C6–H6∙∙∙O1	0.95	2.46	2.828(5)	103	*x*, *y*, *z*
C10–H10∙∙∙O4	0.95	2.57	3.477(5)	160	1 + *x*, *y*, 1 + *z*
C16–H16∙∙∙O2	0.95	2.60	3.397(5)	142	*x*, *y*, *z*
C20–H20∙∙∙S2	0.95	2.73	3.158(3)	108	*x*, *y*, *z*
C20–H20∙∙∙O3	0.95	2.46	2.821(4)	103	*x*, *y*, *z*
**Compound 4**					
N1–H1A∙∙∙O2	0.86	2.14	2.892(3)	145.4	−1 + *x*, *y*, *z*
C6–H6∙∙∙S1	0.93	2.83	3.210(4)	106	*x*, *y*, *z*
C6–H6∙∙∙O1	0.93	2.45	2.800(4)	103	*x*, *y*, *z*

**Table 3:** Geometric parameters of π interactions.

*π*···*π*	Cg ^a^···Cg/ Å	*α* ^b^/º	*β* ^c/^º	Cg···Plane(Cg2)/Å	Offset/ Å	Symm.
**Compound 1**						
S1→C4∙∙∙S1→C4	5.079(3)	0.0(3)	50.0	3.264(2)	3.891	−1 + *x*, *y*, *z*
S1→C4∙∙∙S2→C18	4.246(3)	6.1(2)	33.7	3.751(2)	2.355	*x*, *y*, *z*
C7→C12∙∙∙C7→C12	5.079(2)	0.0(17)	50.4	3.2388(15)	3.912	−1 + *x*, *y*, *z*
C7→C12∙∙∙C21→C26	5.521(2)	0.78(17)	58.4	2.8750(15)	4.703	1 + *x*, *y*, *z*
S2→C18∙∙∙S1→C4	4.246(3)	6.1(2)	27.9	3.5336(19)	1.989	*x*, *y*, *z*
S2→C18∙∙∙S2→C18	5.079(3)	0.0(2)	52.6	3.0846(19)	4.035	−1 + *x*, *y*, *z*
C21→C26∙∙∙C7→C12	5.521(2)	0.78(17)	58.6	2.8916(15)	4.713	−1 + *x*, *y*, −1 + *z*
C21→C26∙∙∙C21→C26	5.079(2)	0.03(17)	49.6	3.2914(15)	3.868	−1 + *x*, *y*, *z*
**Compound 4**						
S1→C4∙∙∙S1→C4	5.128(2)	0.00(19)	54.6	2.9734(16)	4.178	−1 + *x*, *y*, *z*
S1→C4∙∙∙C7→C12	5.269(2)	8.71(18)	41.0	3.4207(16)	3.458	−1 + *x*, *y*, −1 + *z*
C7→C12∙∙∙C7→C12	5.128(2)	0.00(16)	46.1	3.5526(14)	3.698	−1 + *x*, *y*, *z*

^a^ Cg = center of gravity of the ring; ^b^ *α* = angle between planes of two interacting rings; ^c^ *β* = angle between Cg···Cg line and normal to the plane of the first interacting ring.

**Table 4:** Geometric parameters of the C–H···*π* interactions in **4**.

C–H···*π*	H···Cg/ Å	*γ* ^a/^º	C–H···Cg/Å	C···Cg/Å	Symm. Operation on Cg
C15–H15A∙∙∙C7→C12	2.68	11.24	171	3.627(4)	1 + *x*, *y*, *z*

^a^ *γ* = angle defined by a line connecting the center of gravity of the aromatic ring with the H atom and normal to the aromatic ring.

**Table 5:** Inhibition of BChE and calculated IC_50_ values for compounds **1**–**14** and **16**–**19**.

Compound	AChE		BChE	
	% Inhibition *	IC_50_/μM	% Inhibition *	IC_50_/μM
**1**	36.82 ± 0.20 (250)	–	84.37 ± 1.18 (10)	0.500
**2**	30.07 ± 2.38 (100)	–	84.66 ± 3.20 (100)	3.740
**3**	19.01 ± 1.10 (500)	–	82.15 ± 2.00 (500)	149.7
**4**	15.43 ± 0.30 (50)	–	85.38 ± 2.29 (10)	0.913
**5**	34.60 ± 1.39 (100)	–	74.79 ± 2.36 (5)	1.102
**6**	19.80 ± 0,65 (50)	–	74.00 ± 3.20 (50)	14.053
**7**	50.05 ± 1.10 (100)	–	69.80 ± 1.15 (100)	30.403
**8**	87.21 ± 0.47 (500)	275.5	87.21 ± 0.47 (500)	59.108
**9**	28.22 ± 1.06 (100)	–	83.91 ± 1.77 (10)	0.583
**10**	23.33 ± 1.67 (68)	–	83.48 ± 1.49 (27)	3.081
**11**	20.71 ± 0.30 (50)	–	82.75 ± 1.57 (10)	1.541
**12**	17.85 ± 0.51 (100)	–	83.19 ± 0.84 (50)	1.067
**13**	29.87 ± 0.31 (50)	–	84.41 ± 2.29 (25)	1.883
**14**	20.92 ± 0.46 (250)	–	70.00 ± 0.80 (250)	90.001
**16**	23.44 ± 1.57 (100)	–	84.25 ± 0.82 (1)	0.0265
**17**	16.13 ± 1.24 (100)	–	82.28 ± 2.44 (25)	0.645
**18**	22.20 ± 1.17 (25)	–	87.48 ± 1.31 (25)	0.430
**19**	41.35 ± 0.92 (50)	–	79.67 ± 2.51 (50)	2.045
**Galantamine**	90.5 ± 1.3 (60)	0.15	90.10 ± 3.40 (4.5)	7.90

* Numbers given in parentheses represent maximal concentrations tested in μM.

**Table 6:** The mutagenic potential of carbamates **1–19** through Lhasa M7 evaluation (green square—negative, red square—positive, white square—no data available); gray highlight—negative, light gray highlight—positive, white—strongly negative.

Structure	ICH M7 Class	Cohort of Concern	Derek Prediction	Sarah Prediction	Experimental Data	Overall in Silico
**1**	Class 3	No	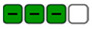	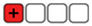	Carc: Unspecified Ames: Unspecified	Positive
**2**	Class 3	No	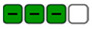	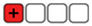	Carc: Unspecified Ames: Unspecified	Positive
**3**	Class 5	No	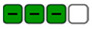	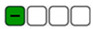	Carc: Unspecified Ames: Unspecified	Negative
**4**	Class 5	No	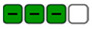	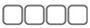	Carc: Unspecified Ames: Unspecified	Negative
**5**	Class 3	No	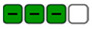	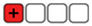	Carc: Unspecified Ames: Unspecified	Positive
**6**	Class 3	No	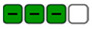	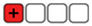	Carc: Unspecified Ames: Unspecified	Positive
**7**	Class 3	No	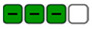	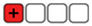	Carc: Unspecified Ames: Unspecified	Positive
**8**	Class 3	No	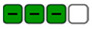	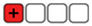	Carc: Unspecified Ames: Unspecified	Positive
**9**	Class 5	No	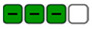	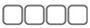	Carc: Unspecified Ames: Unspecified	Negative
**10**	Class 5	No	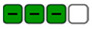	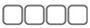	Carc: Unspecified Ames: Unspecified	Negative
**11**	Class 3	No	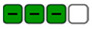	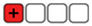	Carc: Unspecified Ames: Unspecified	Positive
**12**	Class 5	No	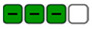	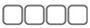	Carc: Unspecified Ames: Unspecified	Negative
**13**	Class 5	No	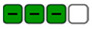	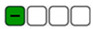	Carc: Unspecified Ames: Unspecified	Negative
**14**	Class 5	No	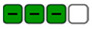	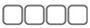	Carc: Unspecified Ames: Unspecified	Negative
**15**	Class 5	No	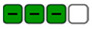	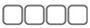	Carc: Unspecified Ames: Unspecified	Negative
**16**	Class 5	No	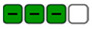	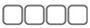	Carc: Unspecified Ames: Unspecified	Negative
**17**	Class 5	No	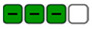	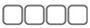	Carc: Unspecified Ames: Unspecified	Negative
**18**	Class 5	No	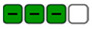	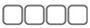	Carc: Unspecified Ames: Unspecified	Negative
**19**	Class 3	No	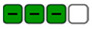	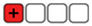	Carc: Unspecified Ames: Unspecified	Positive

**Table 7:** In silico analysis of additional ADME(T) indicators—absorption, distribution, metabolism, excretion, and toxicity—in the human body for carbamates **9**, **16**, and **18**.

Property	Model Name	9	16	18	Bambuterol	Rivastigmine	Unit
**Absorption**	Water solubility	−4.358	−4.272	−4.547			log mol/L
	Caco2	1.907	2.117	1.962			log Papp in 10^−6^ cm/s
	Intestinal absorption	90.33	91.775	92.085			% Absorbed
	Skin permeability	−2.693	−2.743	−2.757			log Kp
	P−glycoprotein substrate	No	No	Yes			
	P−glycoprotein I inhibitor	No	Yes	Yes			
	P−glycoprotein II inhibitor	No	No	No			
**Distribution**	VDss (human)	0.4	0.298	0.324	0.401	0.451	log L/kg
	Fraction unbound	0.022	0.033	0.025	−	−	Fu
	BBB permeability	0.343	0.315	0.261	0.707	0.968	log BB
	CNS permeability	−0.979	−1.069	−1.063	−1.95	−0.801	log PS
**Metabolism**	CYP2D6 substrate	No	No	No			
	CYP3A4 substrate	Yes	Yes	Yes			
	CYP1A2 inhibitor	Yes	Yes	Yes			
	CYP2C19 inhibitor	Yes	Yes	Yes			
	CYP2C9 inhibitor	Yes	No	Yes			
	CYP2D6 inhibitor	No	No	No			
	CYP3A4 inhibitor	No	No	No			
**Excretion**	Total clearance	0.138	0.373	0.262			log ml/min/kg
	Renal OCT2 substrate	Yes	No	No			Yes/no
**Toxicity**	AMES toxicity	No	No	No			Yes/no
	Max. tolerated dose	0.292	−0.157	−0.249			log mg/kg/day
	hERG I inhibitor	No	No	No			
	hERG II inhibitor	Yes	Yes	Yes			
	Oral rat acute toxicity (LD50)	2.648	2.618	2.656			mol/kg
	Oral rat chronic toxicity (LOAEL)	1.896	1.281	1.34			log mg/kg_bw/day
	Hepatotoxicity	Yes	No	No			
	Skin sensitization	No	No	No			
	T. pyriformis toxicity	1.812	1.465	1.542			log ug/L
	Minnow toxicity	0.214	−0.487	−0.62			log mM

## Data Availability

The data are available from the authors on request.

## Supplementary Materials

The following supporting information can be downloaded at: https://www.mdpi.com/article/10.3390/biom15060825/s1, Dose–response curves for the inhibition of BChE by **2**–**8**, **10**–**14,** and **17**–**19**. Mass spectra and HRMS analyses of carbamates **1**–**19**. ^1^H and ^13^C NMR spectra of carbamates **1**–**19**. Cartesian coordinates of docked ligands. Free energies of binding, the number of conformational clusters, and the distribution of conformations obtained by molecular docking. Analysis of MD trajectories (RMSD, RMS fluctuations, and radius of gyration) for enzyme-ligand complexes.
