# Supplementary material for: Heterostilbene Carbamates with Selective and Remarkable Butyrylcholinesterase Inhibition: Computational Study and Physico-Chemical Properties
Source: Biomolecules. 2025 Jun 5;15(6):825. doi: 10.3390/biom15060825 (PMC12191008; doi:10.3390/biom15060825)
Supplement: Supplementary file 1 [file biomolecules-15-00825-s001.zip › biomolecules-3647193-supplementary.pdf]

Electronic Supporting Information

Article

Heterostilbene Carbamates with Selective and Remarkably Butyrylcholinesterase Inhibition: Computational Study and Physico-Chemical Properties

Anamarija Raspudić [1], Ilijana Odak [1], Milena Mlakić [2], Antonija Jelčić [2], Karla Bulava [2], Karla Karadža [2], Valentina Milašinović [3], Ivana Šagud [4], Martina Bosnar [5], Paula Pongrac [5], Dora Štefok [5], Danijela Barić [6,*] and Irena Škorić [1,*]

[1] Department of Chemistry, Faculty of Science and Education, University of Mostar, Matice hrvatske bb, 88 000 Mostar, Bosnia and Herzegovina; ilijana.odak@fpmoz.sum.ba (I.O.); anamarija.raspudic@fpmoz.sum.ba (A.R.)
[2] Department of Organic Chemistry, Faculty of Chemical Engineering and Technology, University of Zagreb, Trg Marka Marulića 19, HR-10 000 Zagreb, Croatia; mdragojev@fkit.unizg.hr (M.M.); ajelcic@fkit.unizg.hr (A.J.); kbulava@fkit.hr (K.B.); kkaradza@fkit.hr
[3] Division of Physical Chemistry, Ruđer Bošković Institute, Bijenička cesta 54, HR-10 000 Zagreb, Croatia; Valentina.Milasinovic@irb.hr (V.M.)
[4] Croatian Agency for Medicinal Products and Medical Devices, Ksaverska Cesta 4, HR-10 000 Zagreb, Croatia; Ivana.Sagud@halmed.hr (I.Š.)
[5] Pharmacology in vitro, Selvita Ltd., Prilaz baruna Filipovića 29, HR-10 000, Zagreb, Croatia; Martina.Bosnar@selvita.com (M.B.); Paula.Pongrac@selvita.com (P.P.); Dora.Stefok@selvita.com (D.S.)
[6] Group for Computational Life Sciences, Division of Physical Chemistry, Ruđer Bošković Institute, Bijenička cesta 54, HR-10 000 Zagreb, Croatia;

* Correspondence: iskoric@fkit.unizg.hr (I.S.); dbaric@irb.hr (D.B.)

Table of contents:

1. Dose-response curves for the inhibition of BChE by **2 – 8**, **10 – 14** and **17 – 19**
2. Mass spectra and HRMS analyses of carbamates **1 – 19**
3. ^{1}H and ^{13}C NMR spectra of carbamates **1 – 19**
4. Figure S69, Validation of molecular docking protocol
5. Cartesian coordinates of ligands 1, 16, and galantamine docked into the active site of BChE
6. Figure S70, Galantamine docked into the active site of BChE
7. Free energies of binding, the number of conformational clusters, and distribution of conformations obtained by molecular docking
8. Analysis of MD trajectories (RMSD, RMS fluctuations, and radius of gyration) for enzyme-ligand complexes

1. Dose-response curves for the inhibition of BChE by carbamates 2 – 8, 10 – 14 and 17 – 19

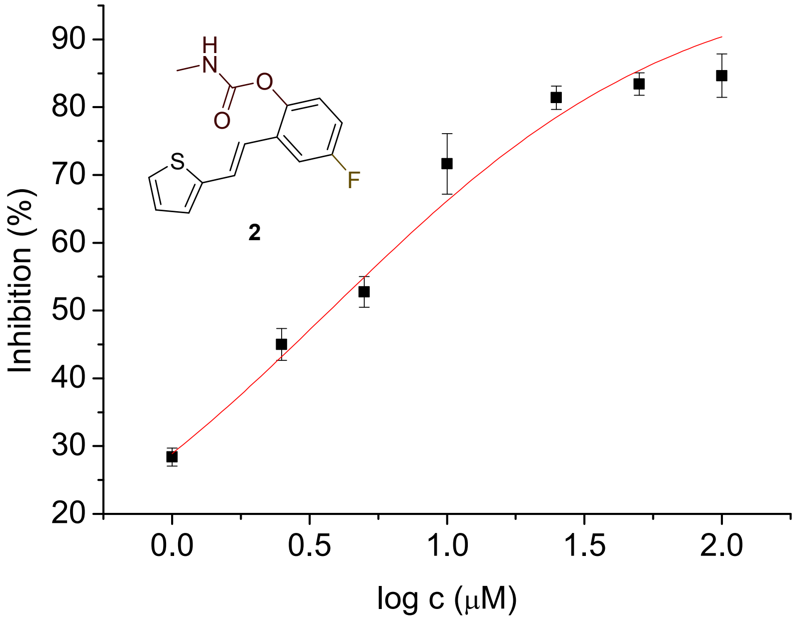

Figure S1. Dose-response curve for the inhibition of BChE by **2**.

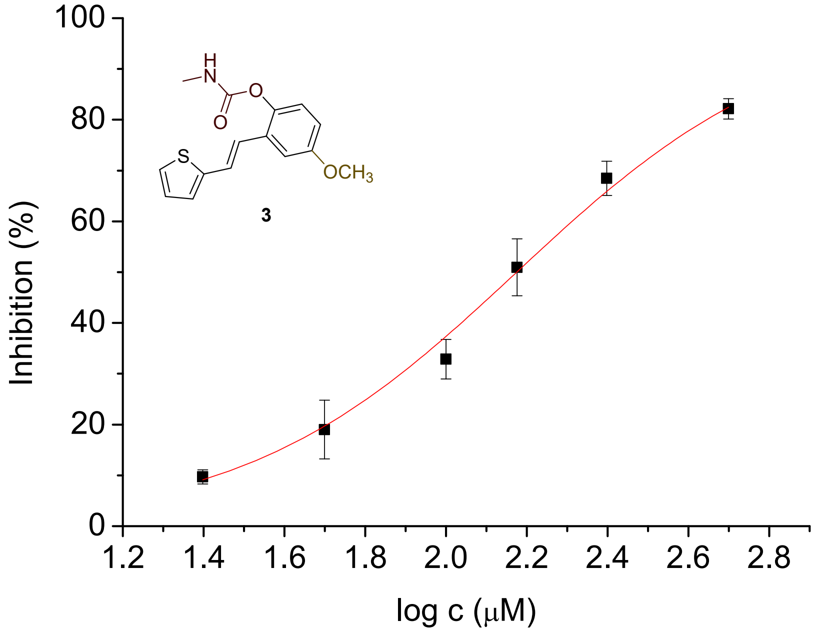

Figure S2. Dose-response curve for the inhibition of BChE by **3**.

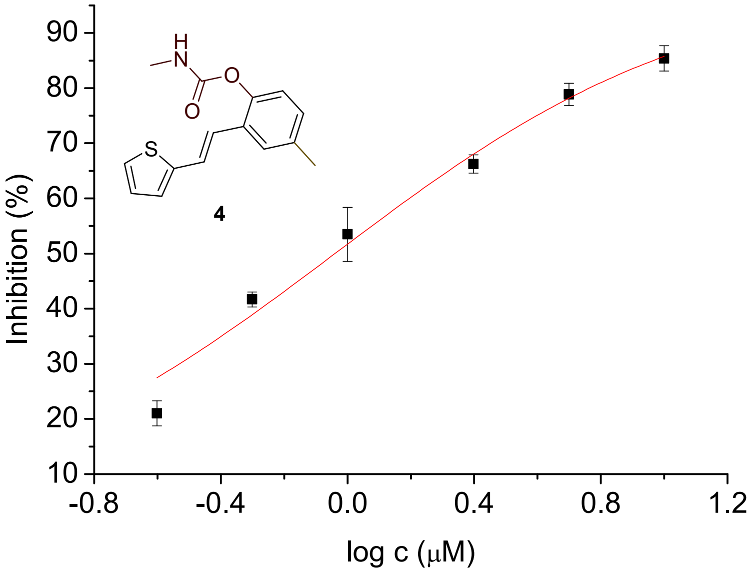

Figure S3. Dose-response curve for the inhibition of BChE by **4**.

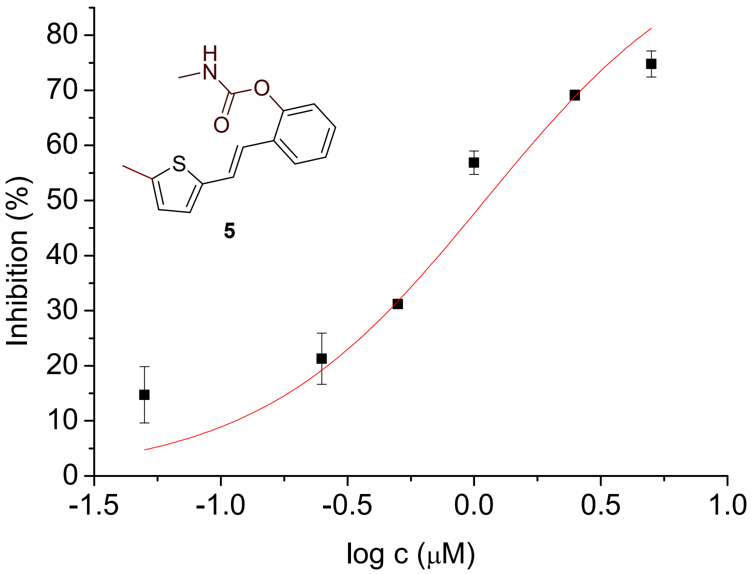

Figure S4. Dose-response curve for the inhibition of BChE by **5**.

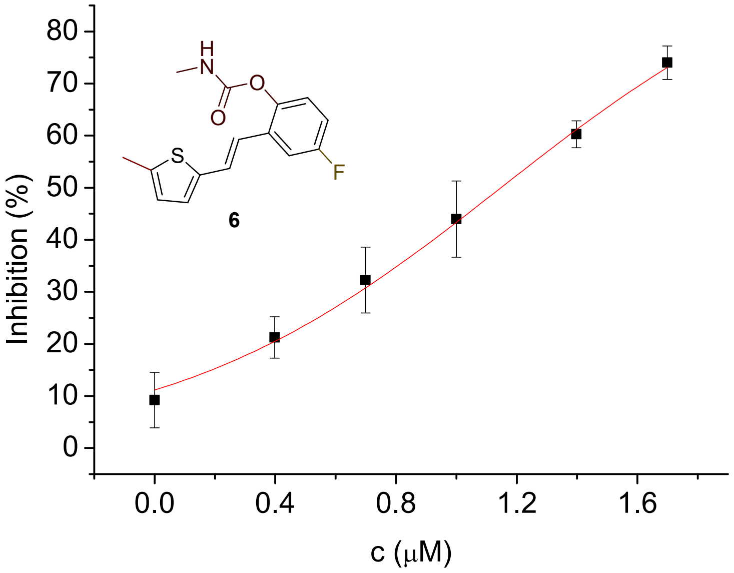

Figure S5. Dose-response curve for the inhibition of BChE by **6**.

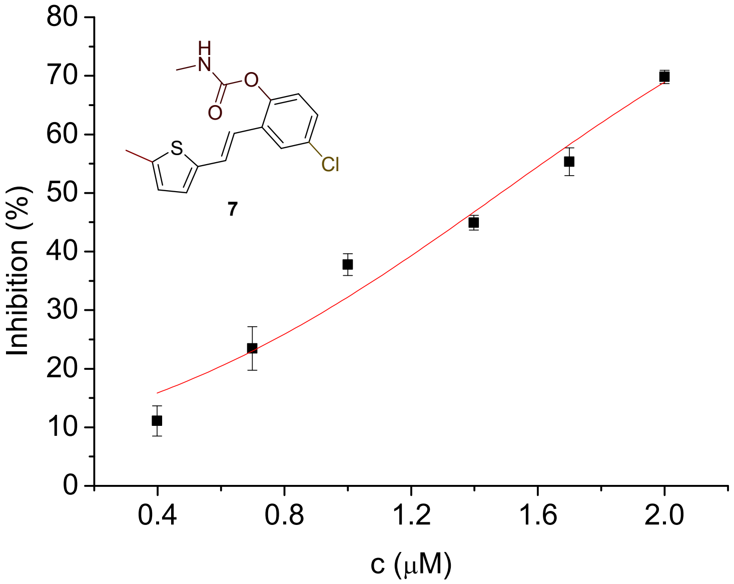

Figure S6. Dose-response curve for the inhibition of BChE by **7**.

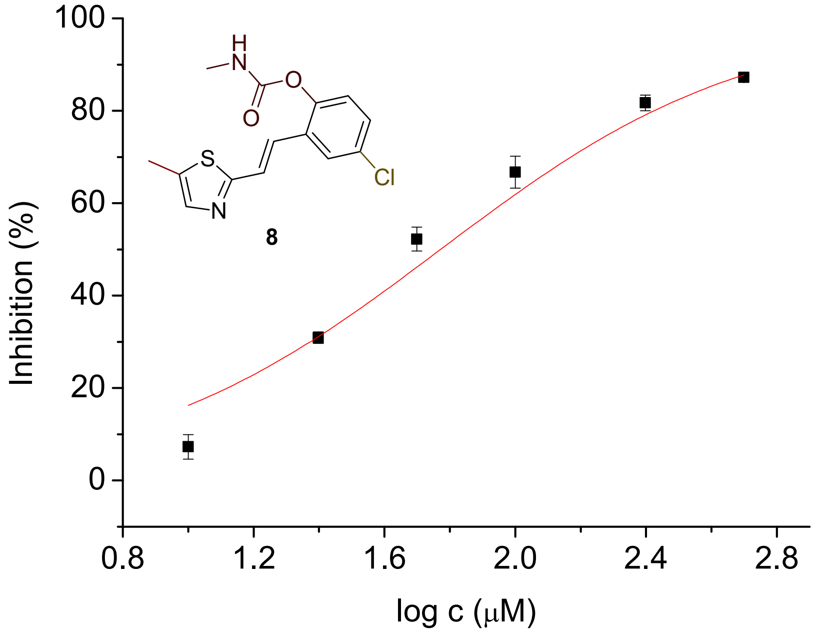

Figure S7. Dose-response curve for the inhibition of BChE by **8**.

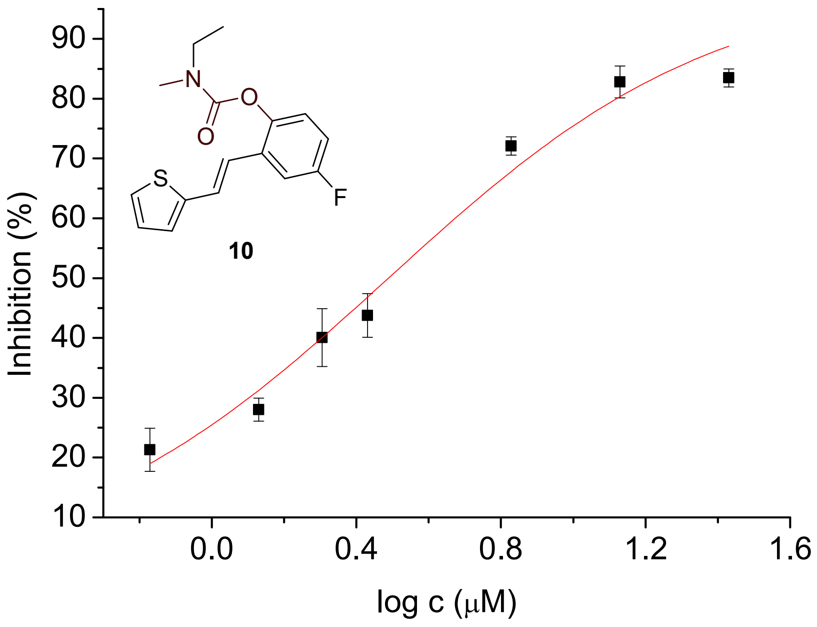

Figure S8. Dose-response curve for the inhibition of BChE by **10**.

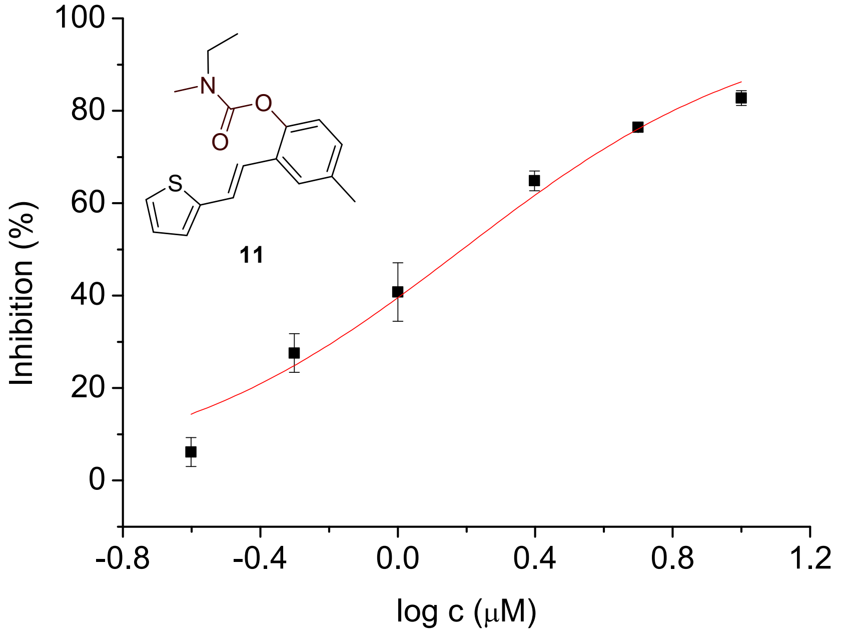

Figure S9. Dose-response curve for the inhibition of BChE by **11**.

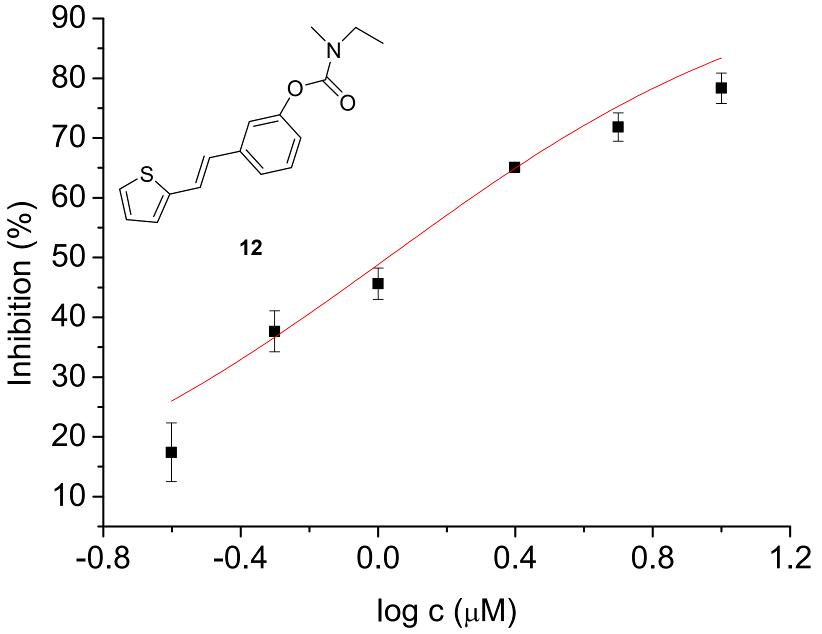

Figure S10. Dose-response curve for the inhibition of BChE by **12**.

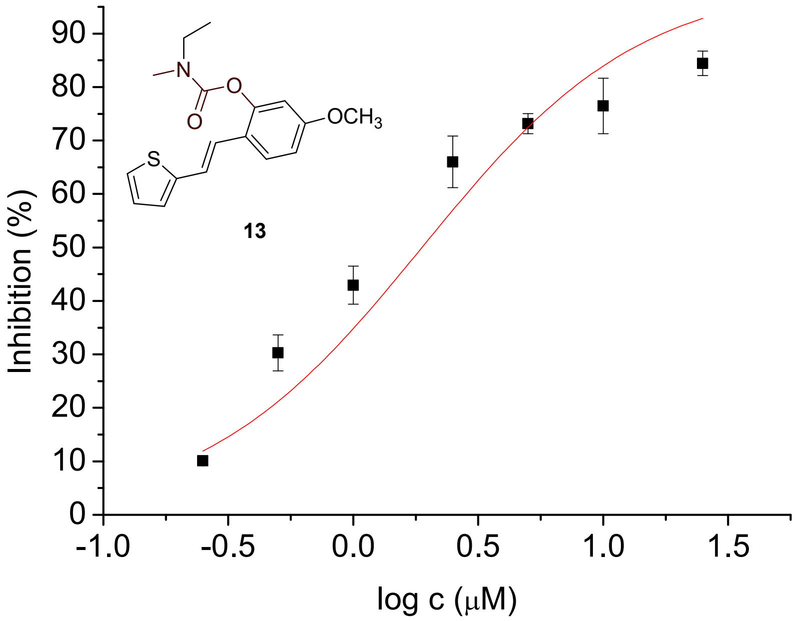

Figure S11. Dose-response curve for the inhibition of BChE by **13**.

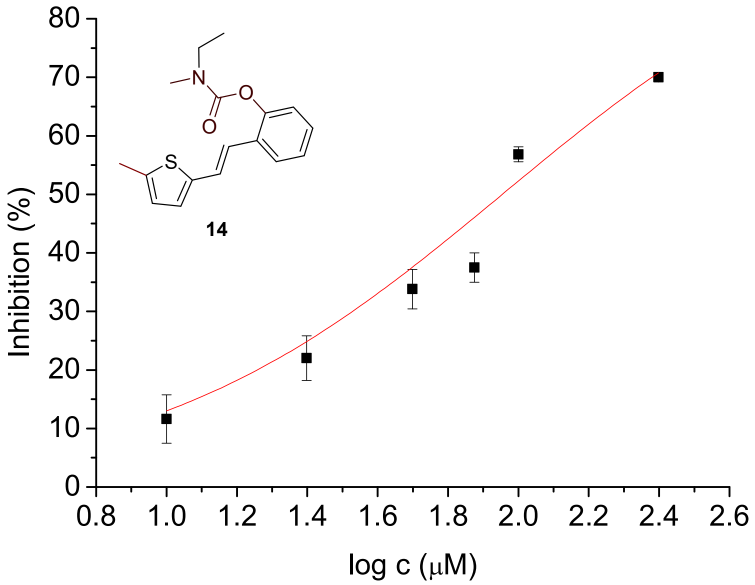

Figure S12. Dose-response curve for the inhibition of BChE by **14**.

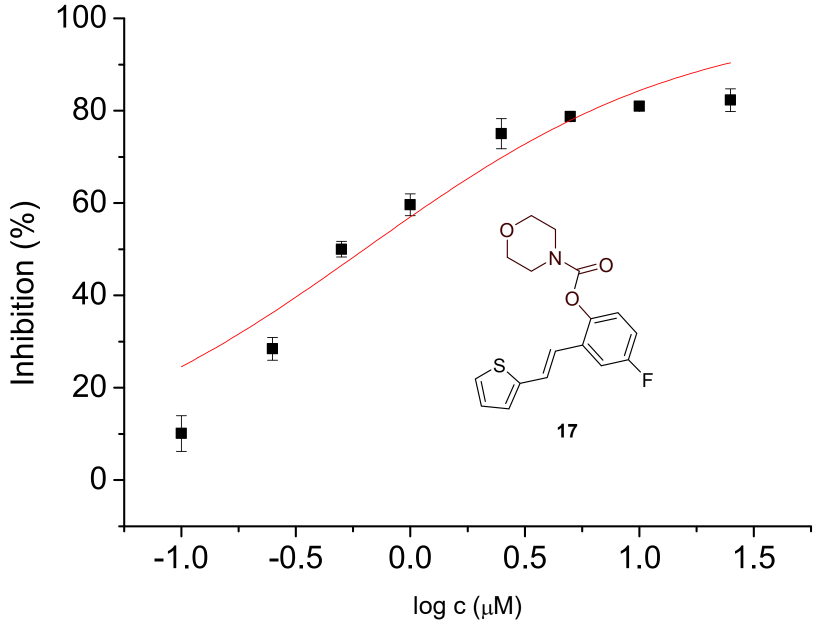

Figure S13. Dose-response curve for the inhibition of BChE by **17**.

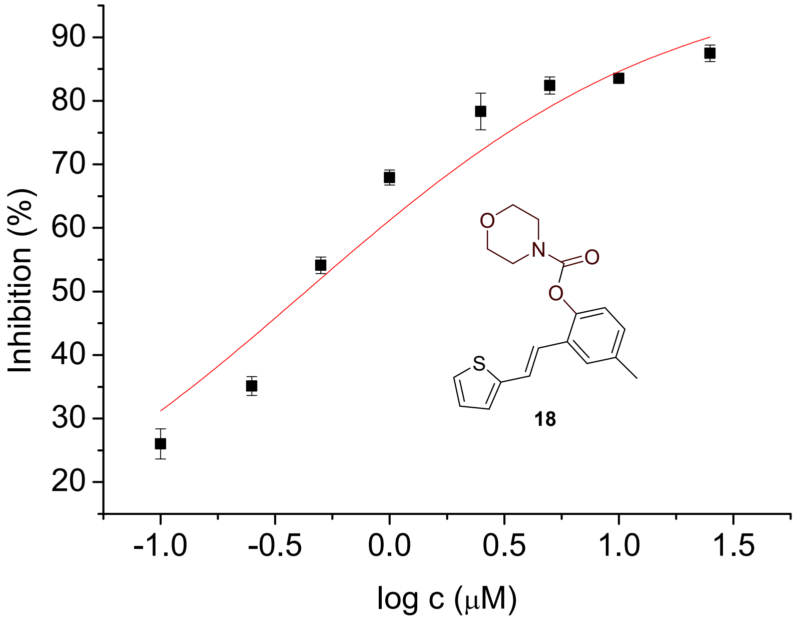

Figure S14. Dose-response curve for the inhibition of BChE by **18**.

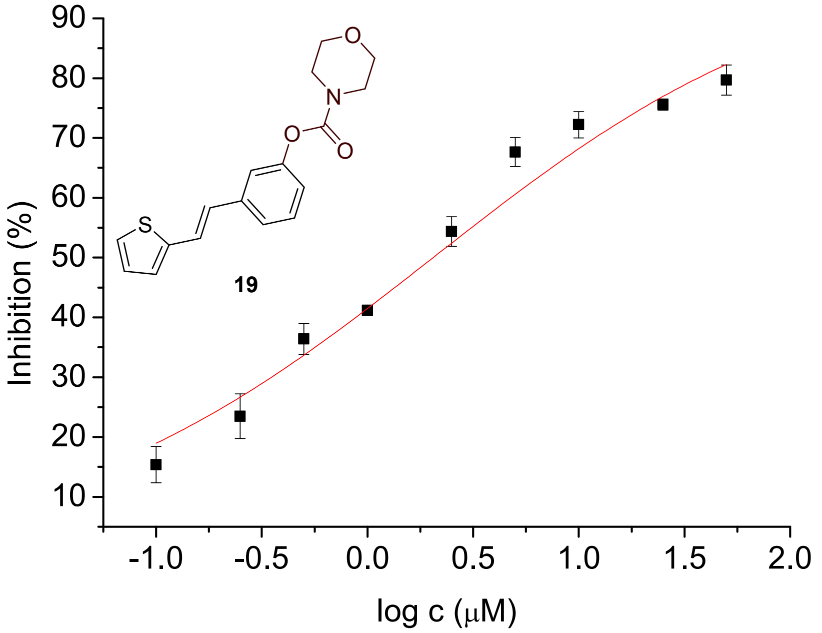

Figure S15. Dose-response curve for the inhibition of BChE by **19**.

2. Mass spectra and HRMS analyses of carbamates 1 – 19

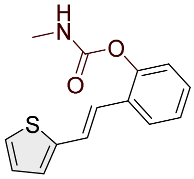

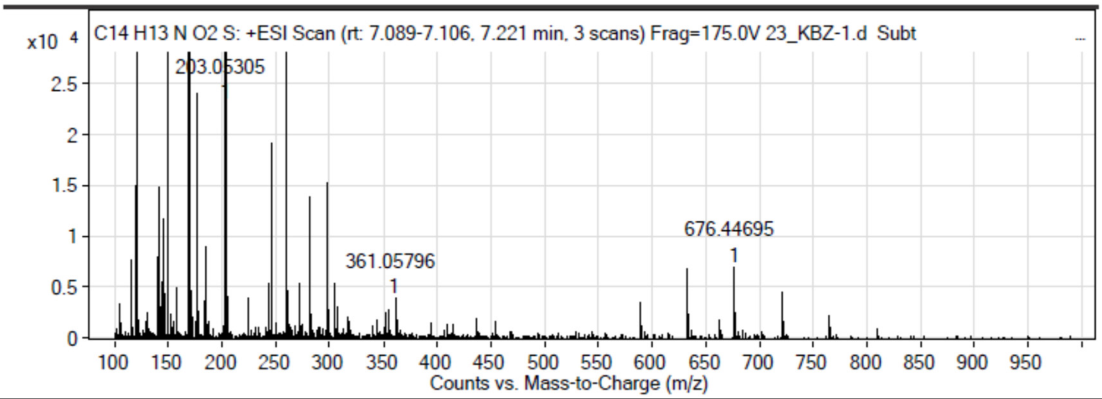

Formula Calculator Results

Formula	Best	Mass	Tgt Mass	Diff (ppm)	Ion Species	Score
C14 H13 N O2 S	True	259.06662	259.0667	0.32	C14 H14 N O2 S	99.01

Figure S16. Mass spectra and HRMS analysis of carbamate **1**.

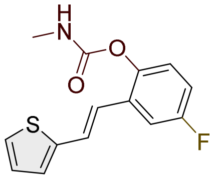

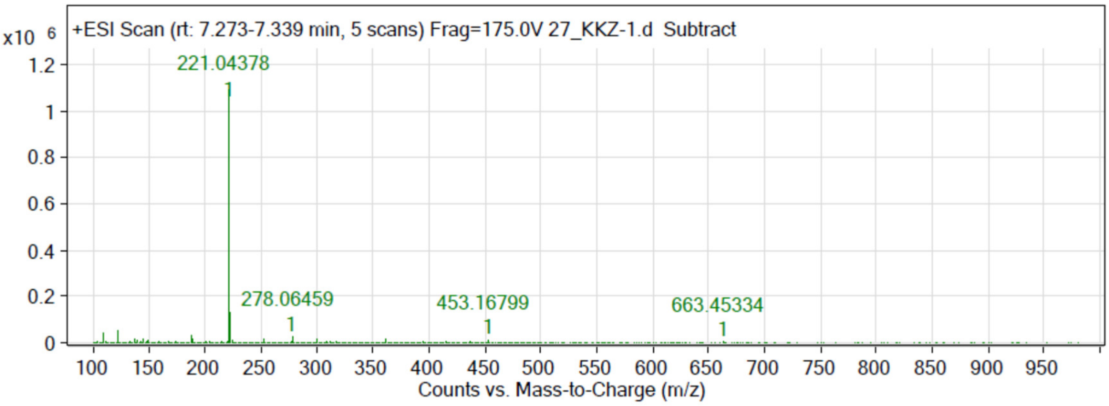

Formula Calculator Results

Formula	Best	Mass	Tgt Mass	Diff (ppm)	Ion Species	Score
C14 H12 F N O2 S	True	277.05727	277.05728	0.04	C14 H13 F N O2 S	98.79

Figure S17. Mass spectra and HRMS analysis of carbamate **2**.

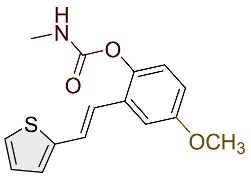

Peak List

m/z	z	Abund	Formula	Ion
149.02346		37591.32		
233.06391	1	251074.22		
234.06681	1	33451.94		
279.09362	1	31940.43		
290.08461	1	40905.91	C15 H15 N O3 S	(M+H)+
293.07568	1	32330.35		
312.06647	1	29945.77		
350.25446	1	53062.36		
720.47394	1	39330.24		
764.50024	1	41317.5		

Formula Calculator Element Limits

Element	Min	Max
C	3	60
H	0	120
O	0	30
N	0	30
S	0	5
Cl	0	3

Formula Calculator Results

Formula	Best	Mass	Tgt Mass	Diff (ppm)	Ion Species	Score
C15 H15 N O3 S	True	289.07733	289.07726	-0.21	C15 H16 N O3 S	99.27

Figure S18. Table with masses and HRMS analysis of carbamate **3**.

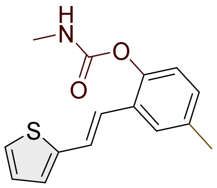

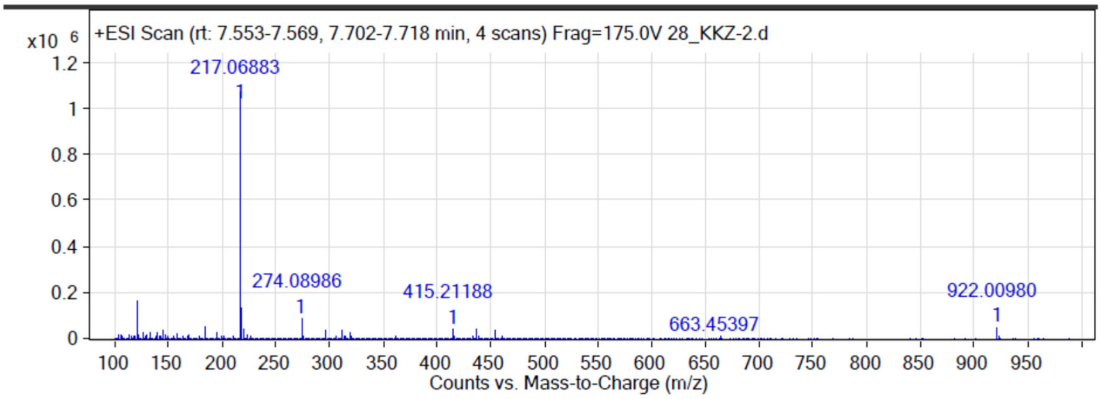

Formula Calculator Results

Formula	Best	Mass	Tgt Mass	Diff (ppm)	Ion Species	Score
C15 H15 N O2 S	True	273.08261	273.08235	-0.95	C15 H16 N O2 S	98.46

Figure S19. Mass spectra and HRMS analysis of carbamate **4**.

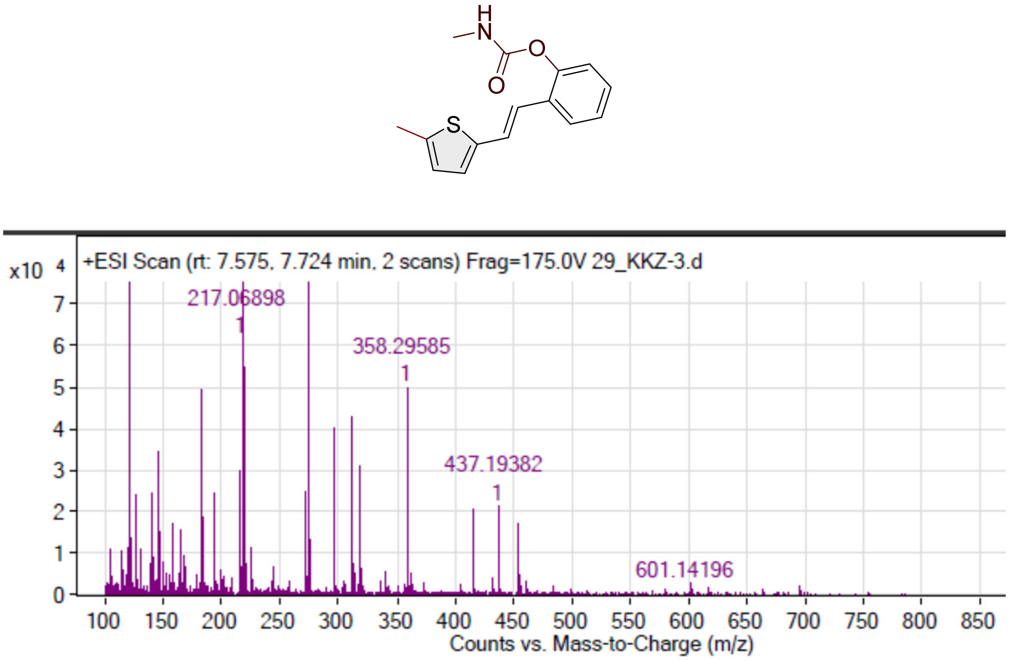

Formula Calculator Results

Formula	Best	Mass	Tgt Mass	Diff (ppm)	Ion Species	Score
C15 H15 N O2 S	False	273.0825	273.08235	-0.57	C15 H16 N O2 S	99.26

Figure S20. Mass spectra and HRMS analysis of carbamate **5**.

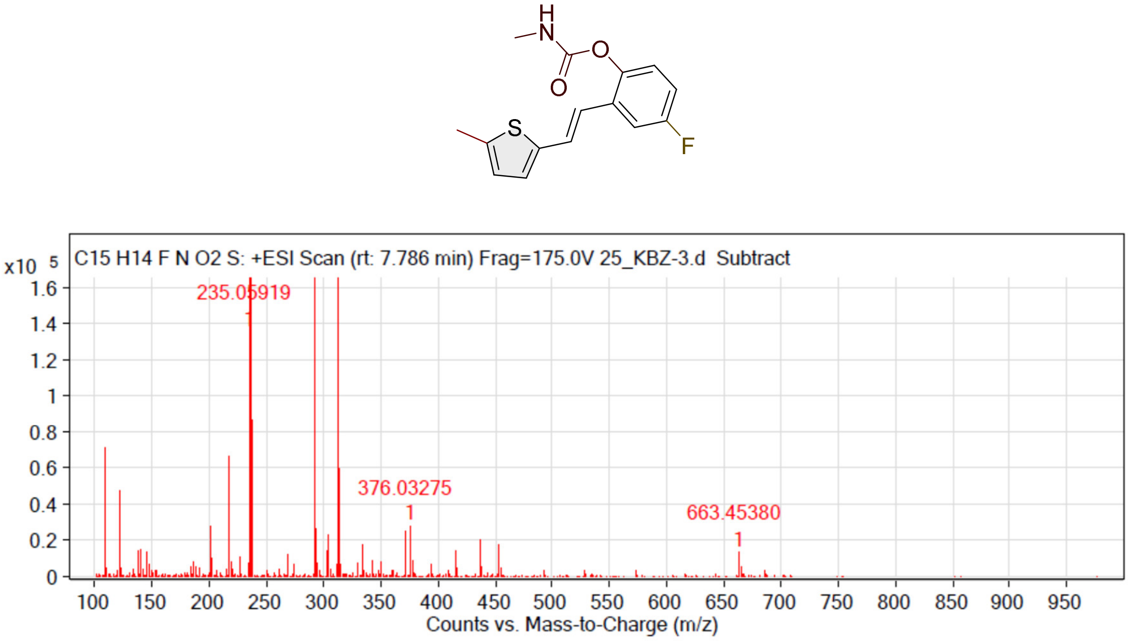

Formula Calculator Results

Formula	Best	Mass	Tgt Mass	Diff (ppm)	Ion Species	Score
C15 H14 F N O2 S	True	291.07308	291.07293	-0.53	C15 H15 F N O2 S	98.4

Figure S21. Mass spectra and HRMS analysis of carbamate **6**.

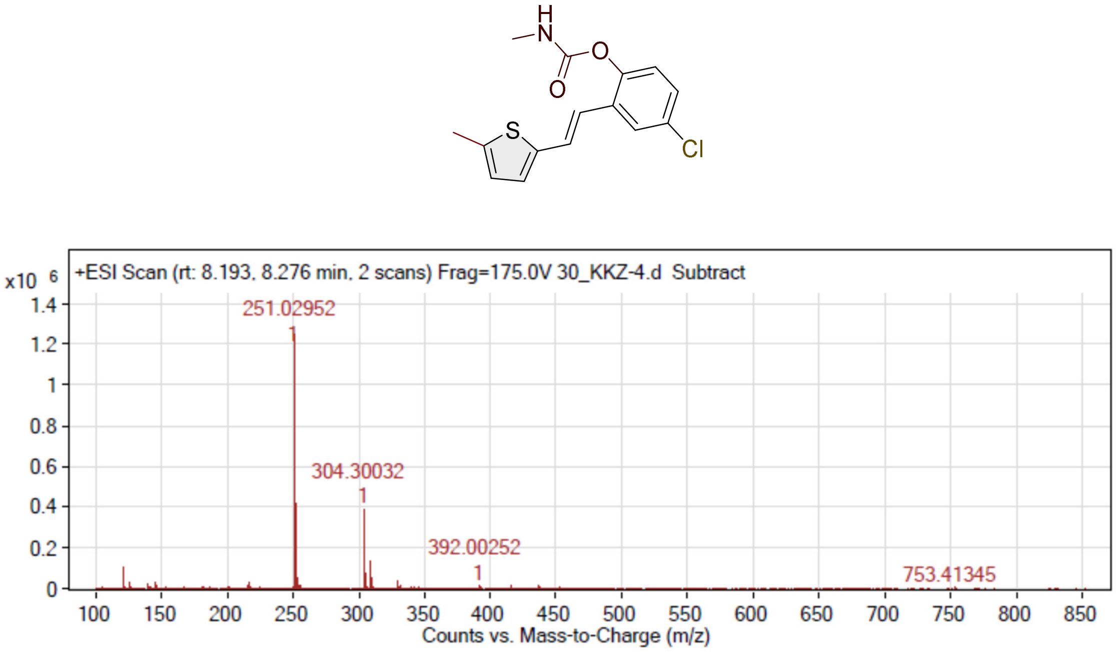

Formula Calculator Results

Formula	Best	Mass	Tgt Mass	Diff (ppm)	Ion Species	Score
C15 H14 Cl N O2 S	True	307.04329	307.04338	0.28	C15 H15 Cl N O2 S	99.2

Figure S22. Mass spectra and HRMS analysis of carbamate **7**.

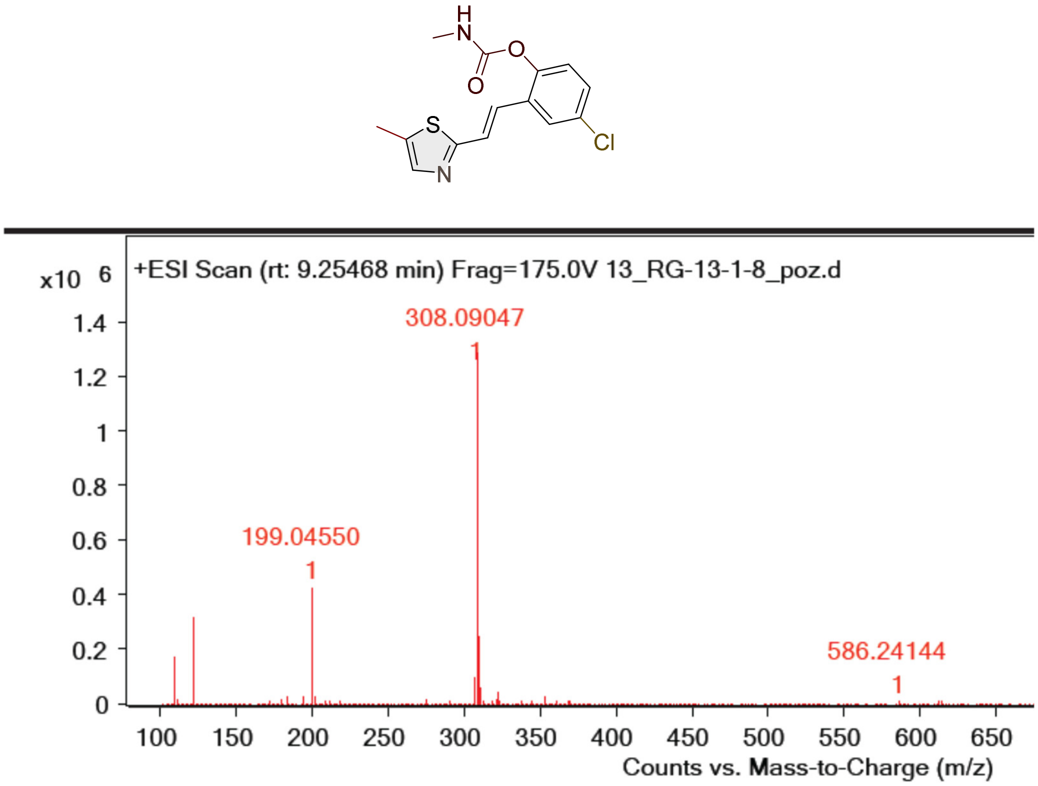

Figure S23. Mass spectra of carbamate **8**.

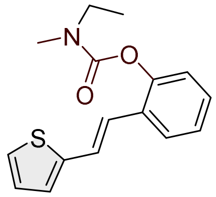

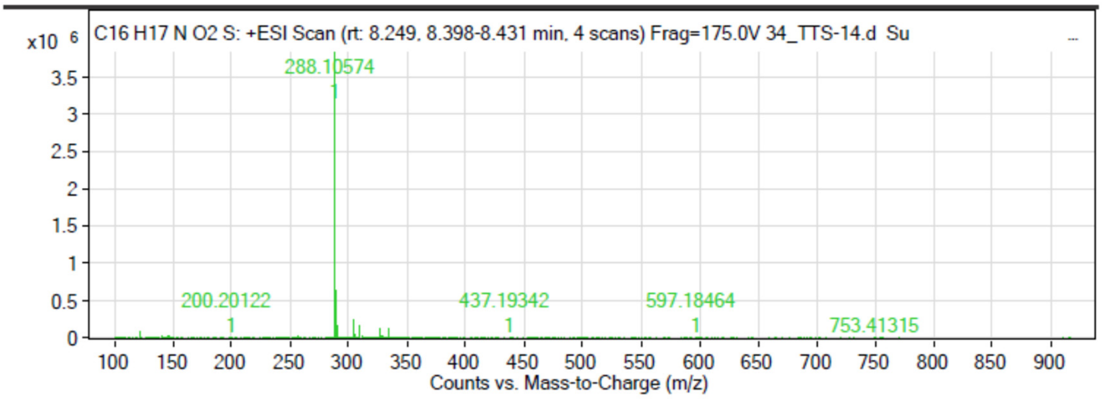

Formula Calculator Results

Formula	Best	Mass	Tgt Mass	Diff (ppm)	Ion Species	Score
C16 H17 N O2 S	True	287.09853	287.098	-1.84	C16 H18 N O2 S	96.51

Figure S24. Mass spectra and HRMS analysis of carbamate **9**.

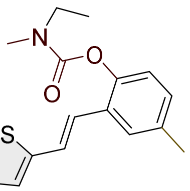

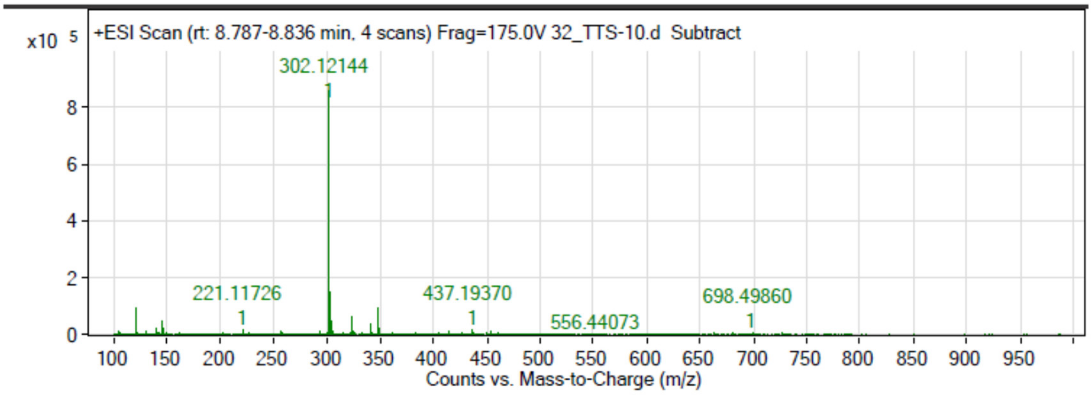

Formula Calculator Results

Formula	Best	Mass	Tgt Mass	Diff (ppm)	Ion Species	Score
C17 H19 N O2 S	False	301.11414	301.11365	-1.64	C17 H20 N O2 S	96.49

Figure S25. Mass spectra and HRMS analysis of carbamate **11**.

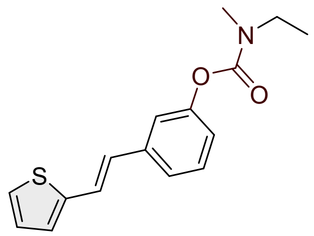

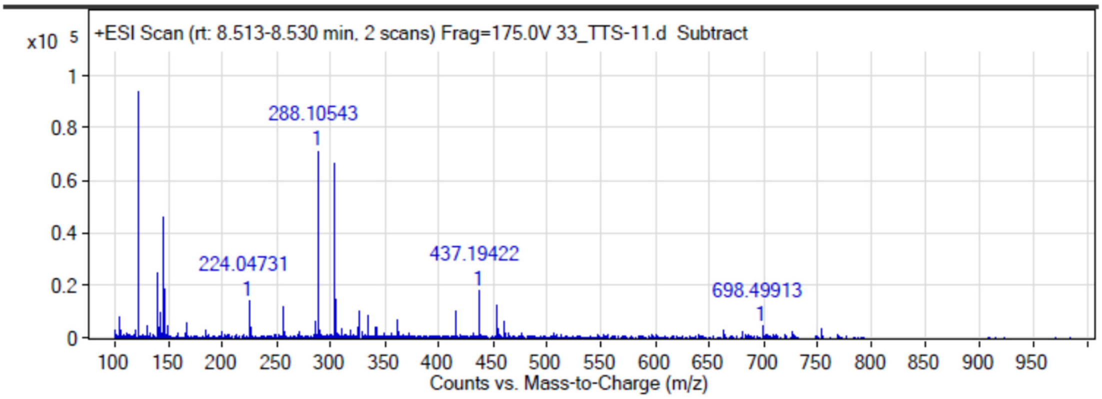

Formula Calculator Results

Formula	Best	Mass	Tgt Mass	Diff (ppm)	Ion Species	Score
C16 H17 N O2 S	True	287.09817	287.098	-0.6	C16 H18 N O2 S	98.11

Figure S26. Mass spectra and HRMS analysis of carbamate **12**.

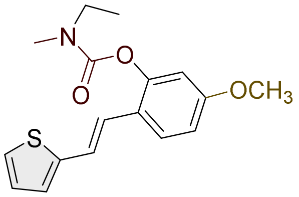

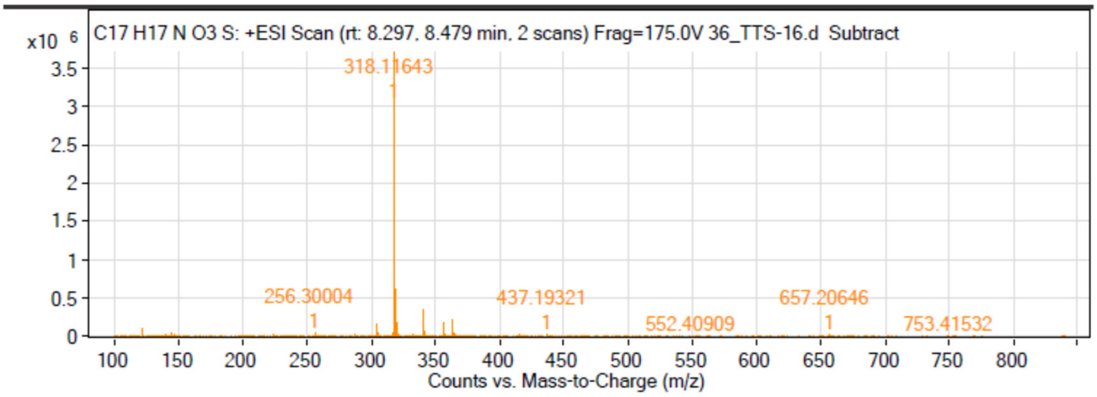

Figure S27. Mass spectra of carbamate **13**.

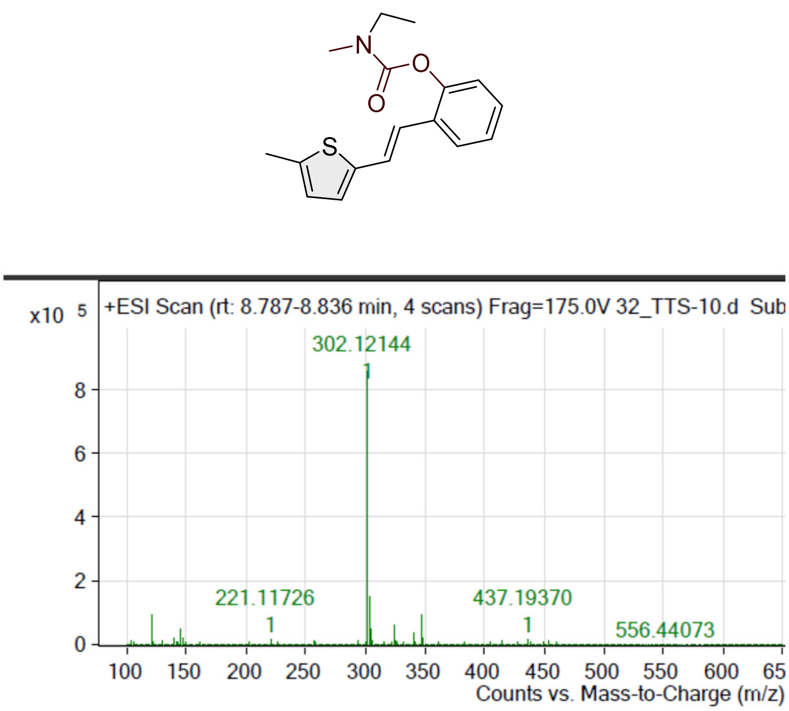

Figure S28. Mass spectra of carbamate **14**.

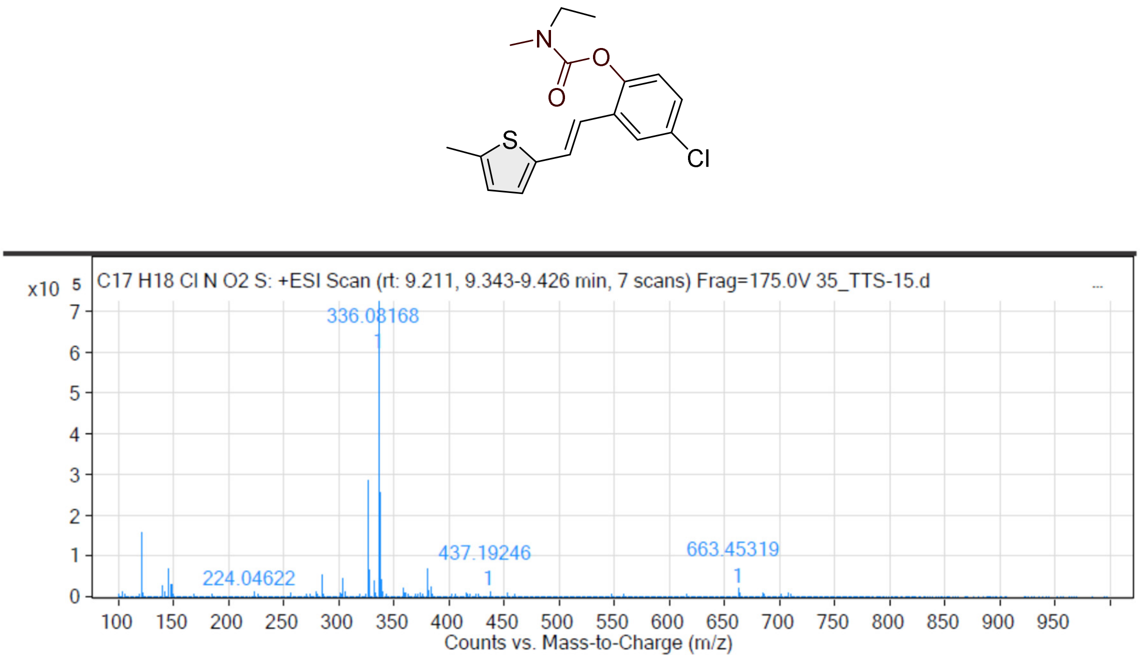

Formula Calculator Results

Formula	Best	Mass	Tgt Mass	Diff (ppm)	Ion Species	Score
C17 H18 Cl N O2 S	True	335.07437	335.07468	0.9	C17 H19 Cl N O2 S	98.32

Figure S29. Mass spectra and HRMS analysis of carbamate **15**.

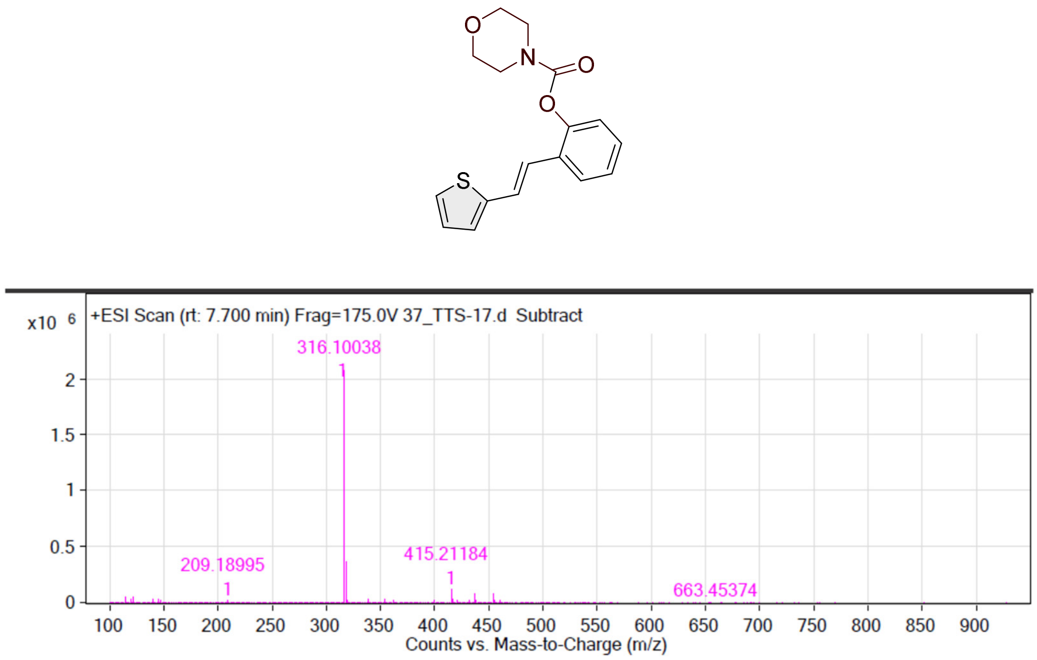

Formula Calculator Results

Formula	Best	Mass	Tgt Mass	Diff (ppm)	Ion Species	Score
C17 H17 N O3 S	True	315.0932	315.09291	-0.91	C17 H18 N O3 S	96.27

Figure S30. Mass spectra and HRMS analysis of carbamate **16**.

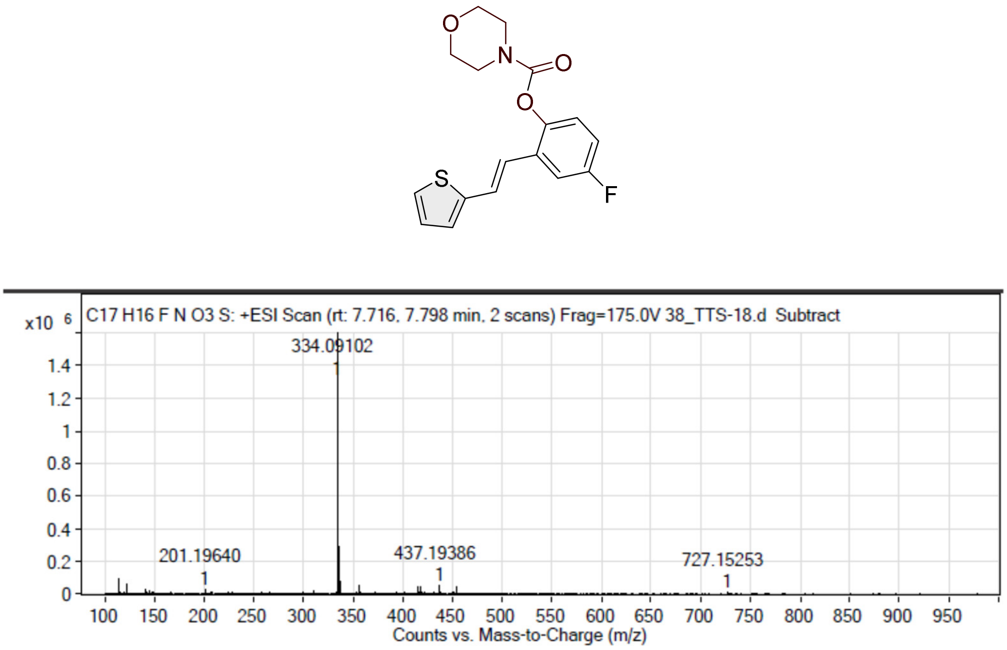

Formula Calculator Results

Formula	Best	Mass	Tgt Mass	Diff (ppm)	Ion Species	Score
C17 H16 F N O3 S	True	333.08379	333.08349	-0.89	C17 H17 F N O3 S	97.71

Figure S31. Mass spectra and HRMS analysis of carbamate **17**.

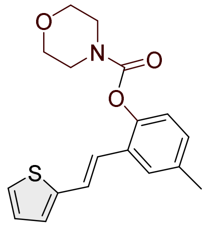

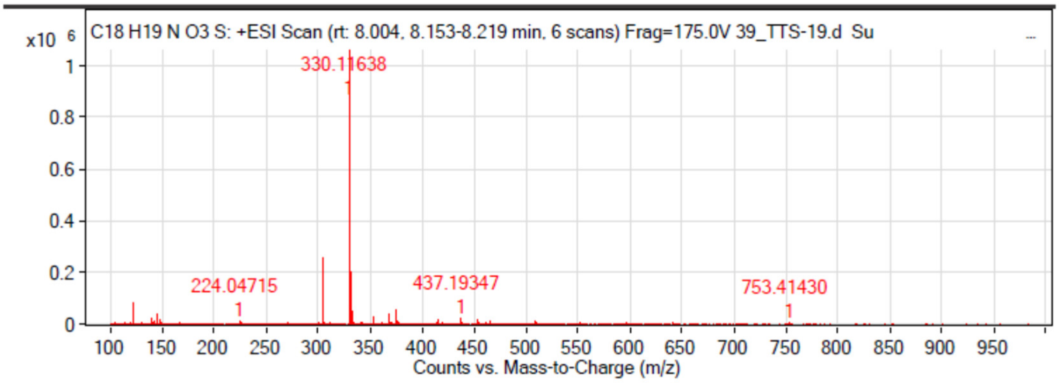

Formula Calculator Results

Formula	Best	Mass	Tgt Mass	Diff (ppm)	Ion Species	Score
C18 H19 N O3 S	True	329.10913	329.10856	-1.72	C18 H20 N O3 S	96.7

Figure S32. Mass spectra and HRMS analysis of carbamate **18**.

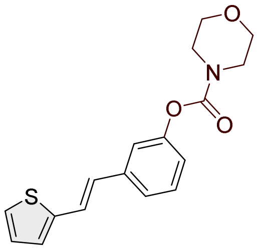

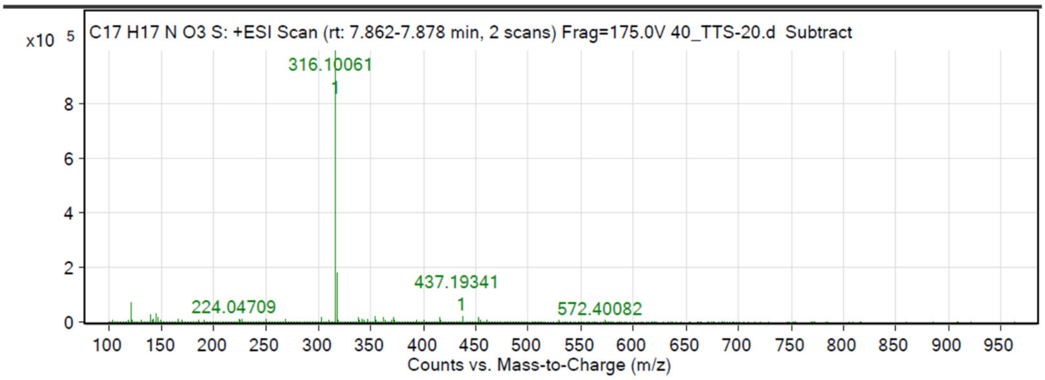

Formula Calculator Results

Formula	Best	Mass	Tgt Mass	Diff (ppm)	Ion Species	Score
C17 H17 N O3 S	True	315.09343	315.09291	-1.63	C17 H18 N O3 S	96.77

Figure S33. Mass spectra and HRMS analysis of carbamate **19**.

3. ^{1}H and ^{13}C NMR spectra of carbamates 1 – 19

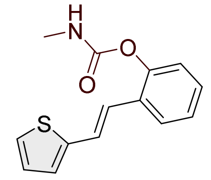

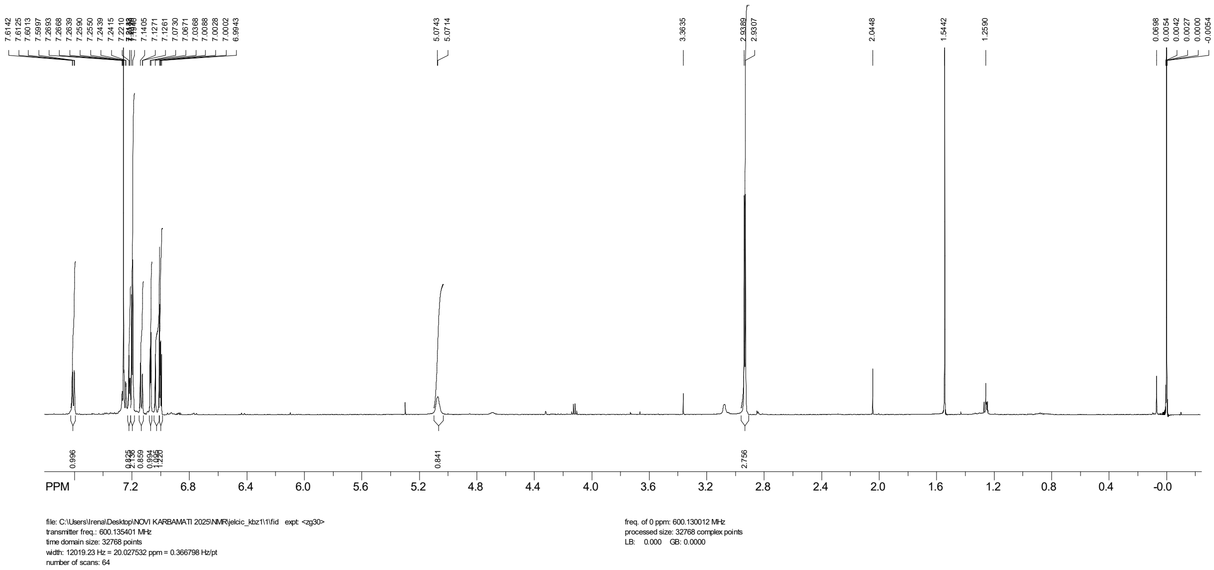

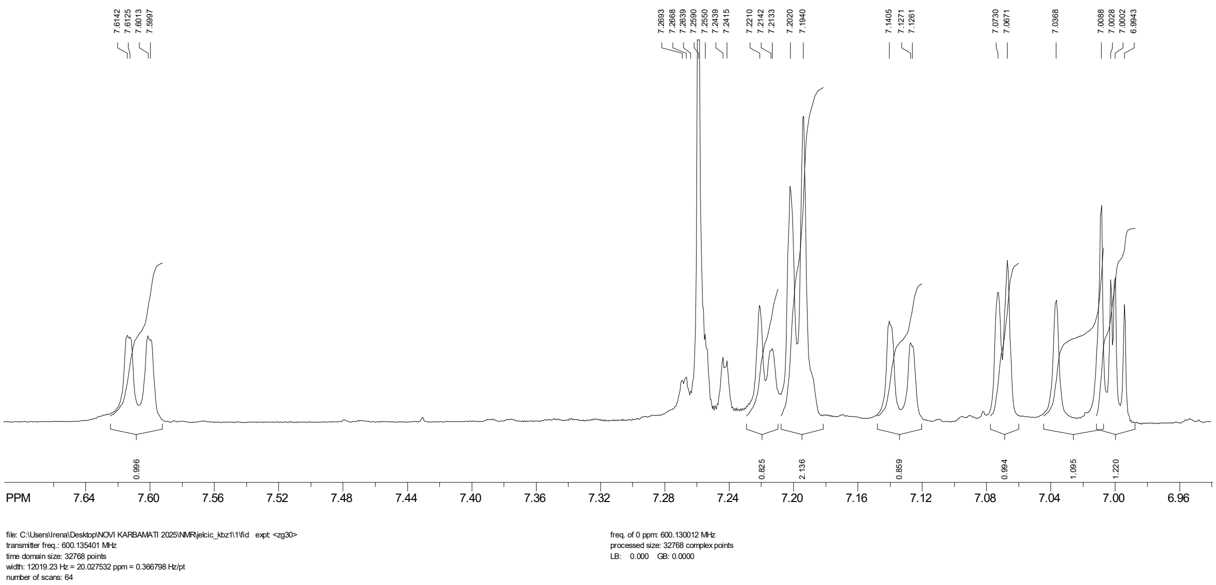

Figure S34. ^{1}H NMR spectrum ($CDCl_3$) of carbamate **1**.

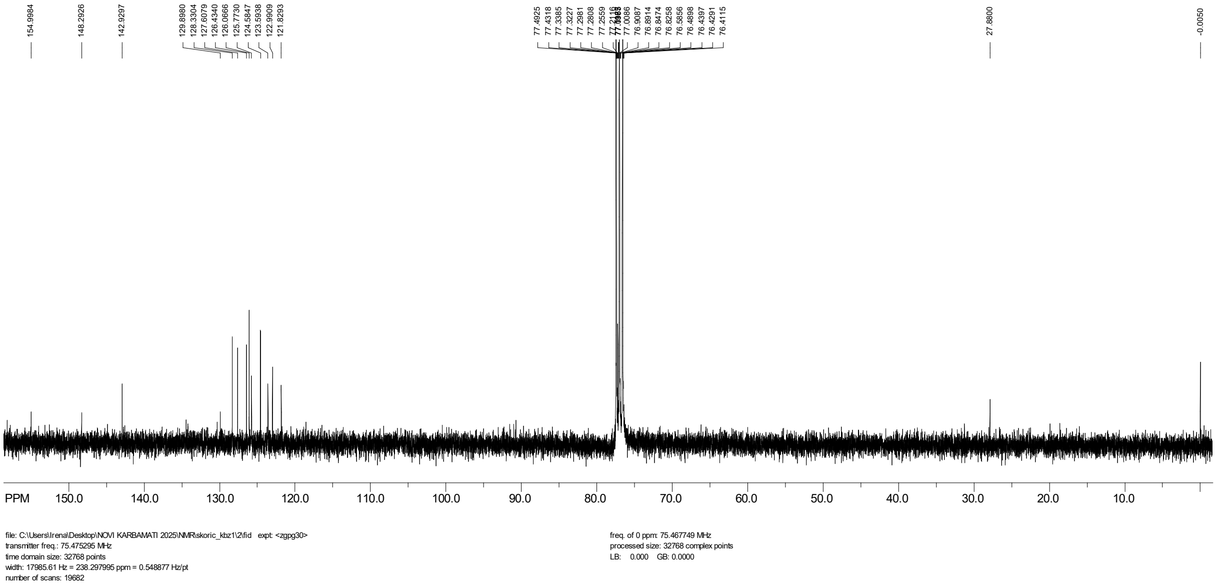

Figure S35. ^{13}C NMR spectrum ($CDCl_3$) of carbamate **1**.

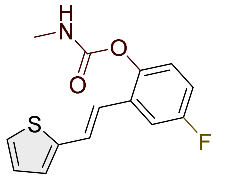

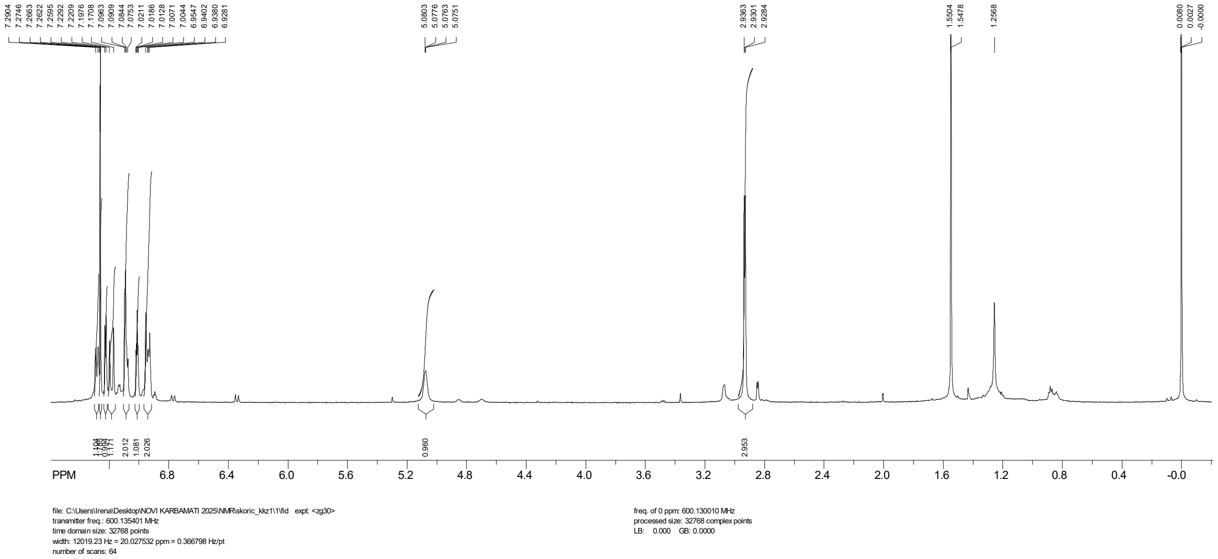

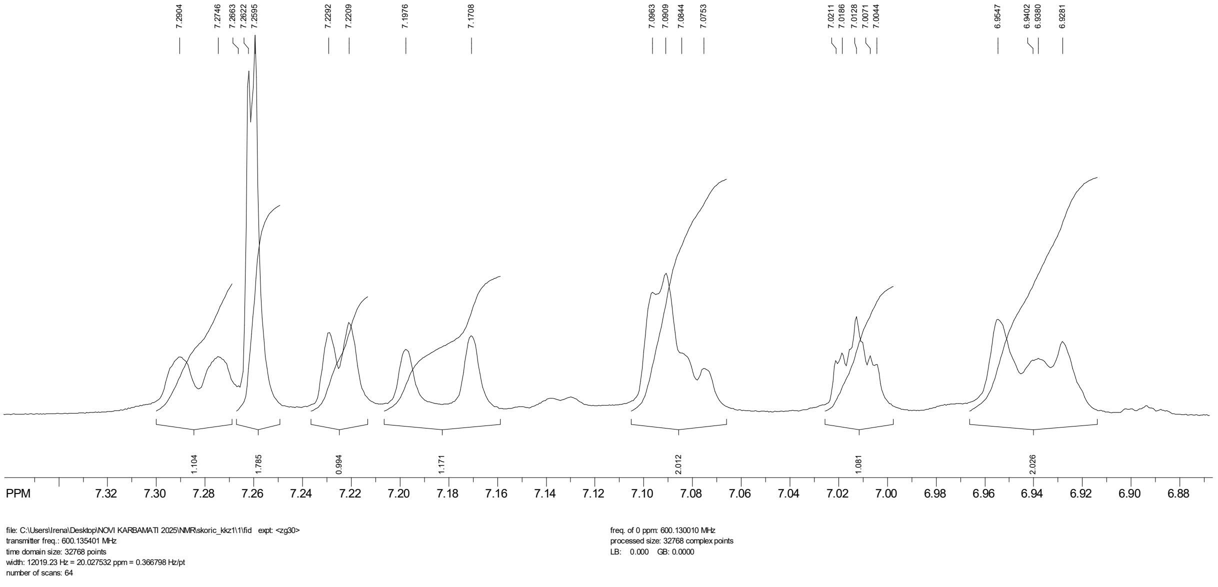

Figure S36. ^{1}H NMR spectrum ($CDCl_3$) of carbamate **2**.

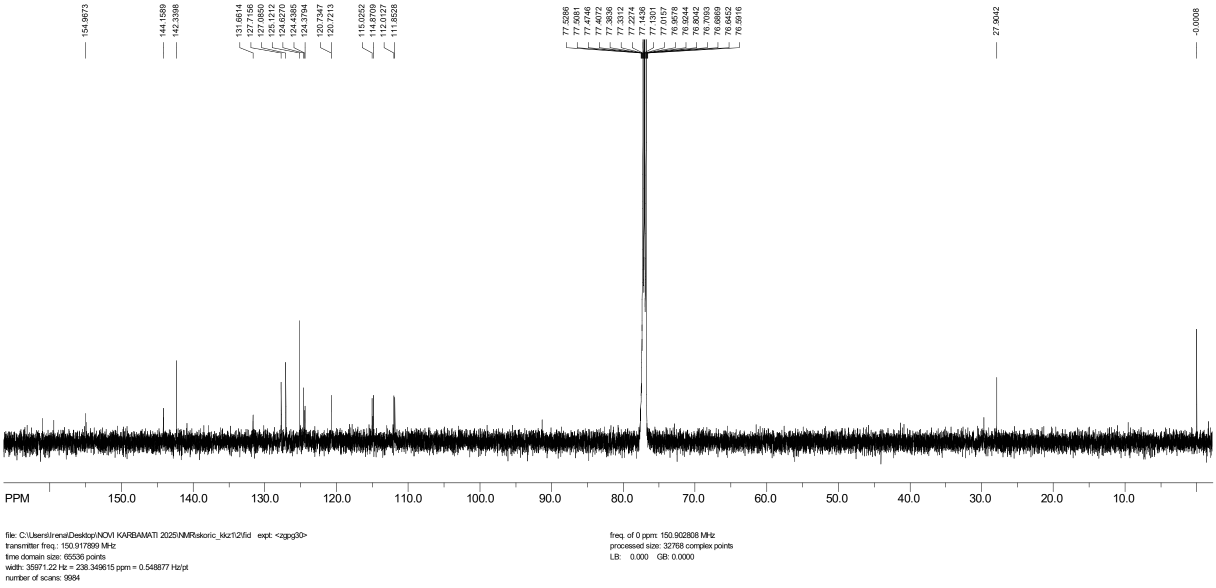

Figure S37. ^{13}C NMR spectrum ($CDCl_3$) of carbamate **2**.

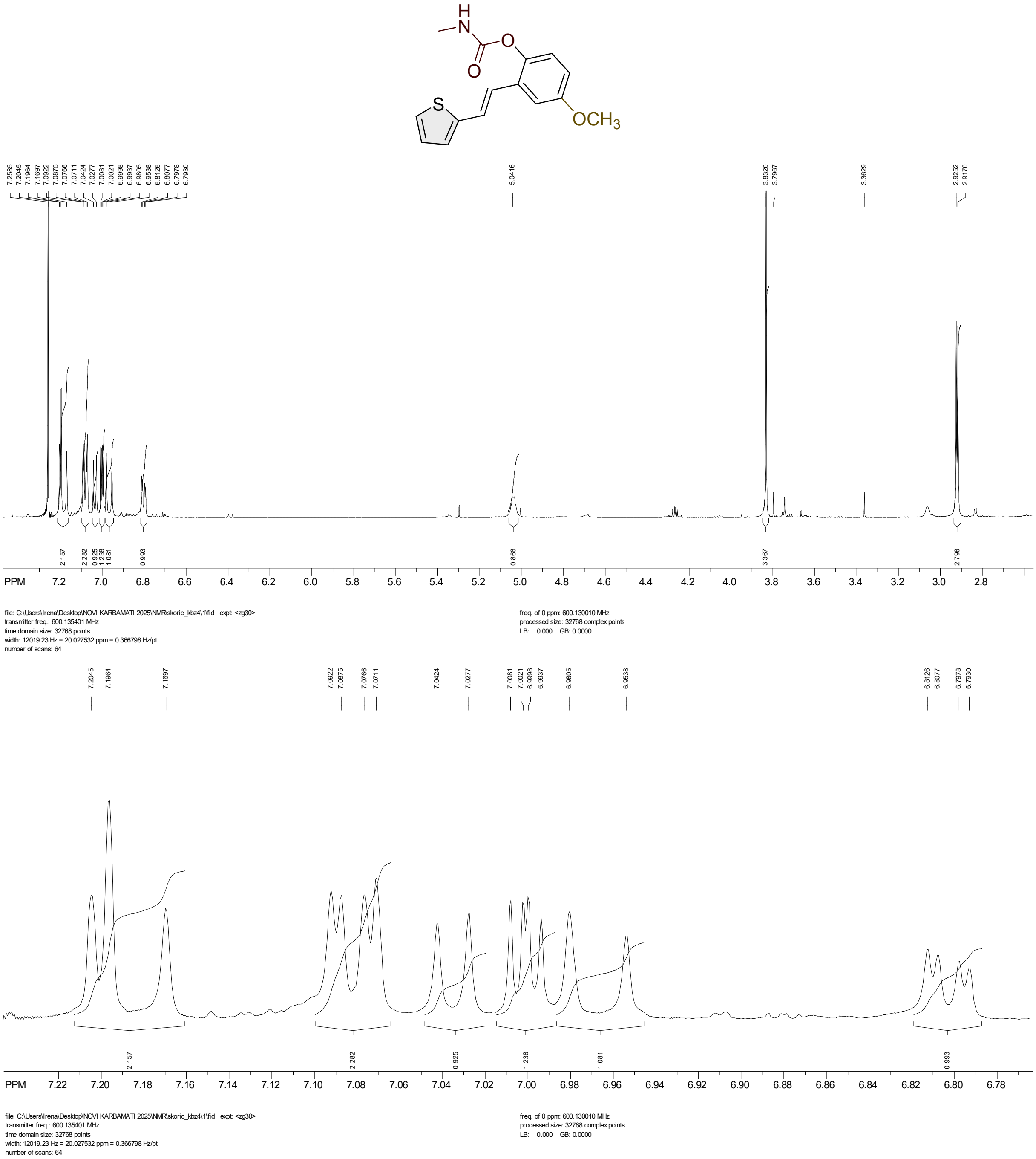

Figure S38. ^{1}H NMR spectrum ($CDCl_3$) of carbamate **3**.

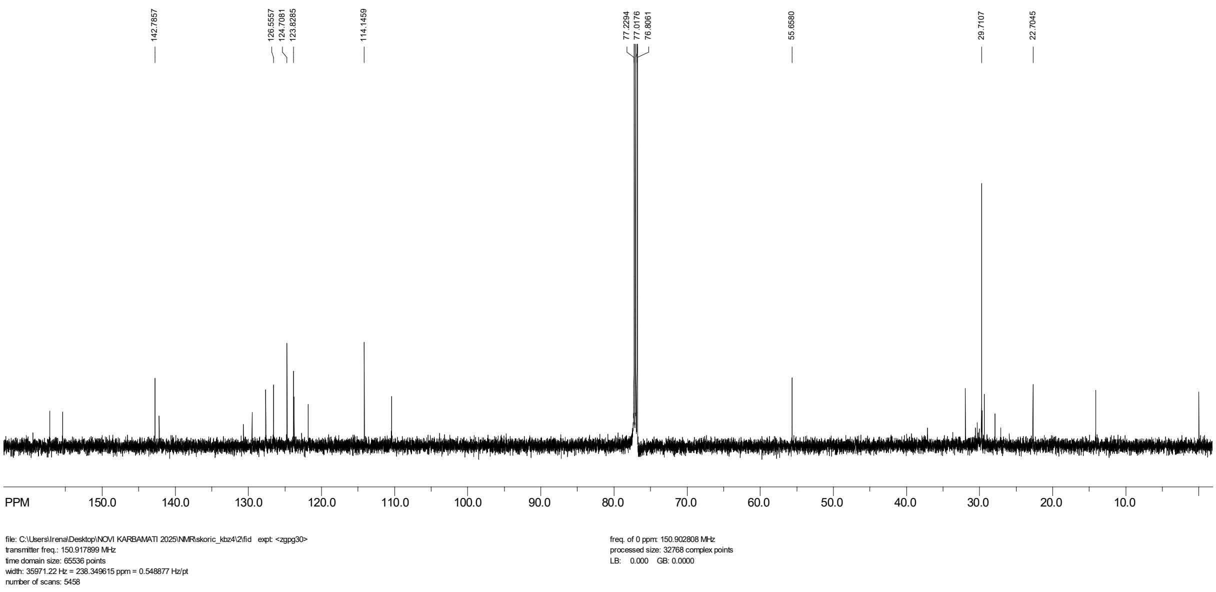

Figure S39. ^{13}C NMR spectrum ($CDCl_3$) of carbamate **3**.

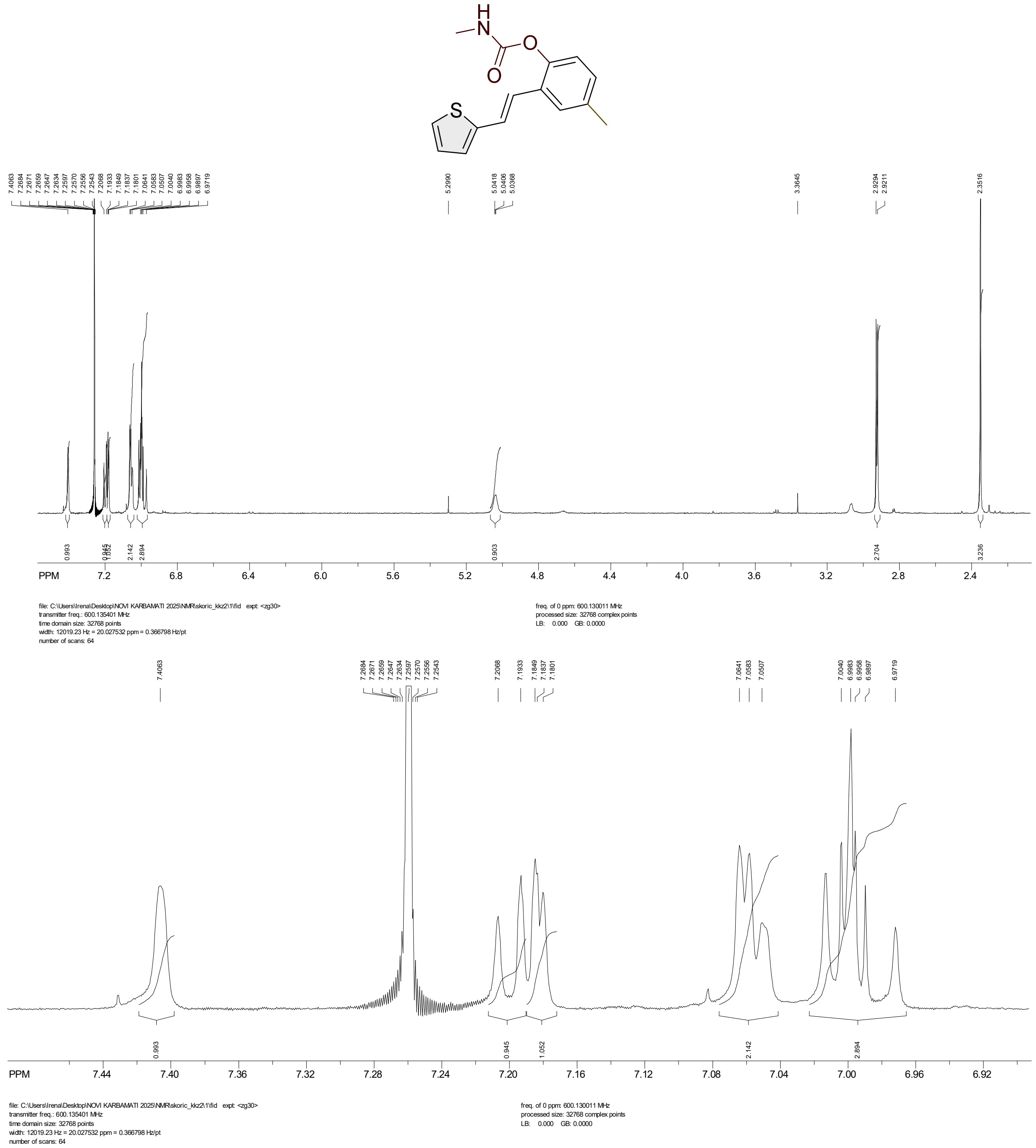

Figure S40. ^{1}H NMR spectrum ($CDCl_3$) of carbamate **4**.

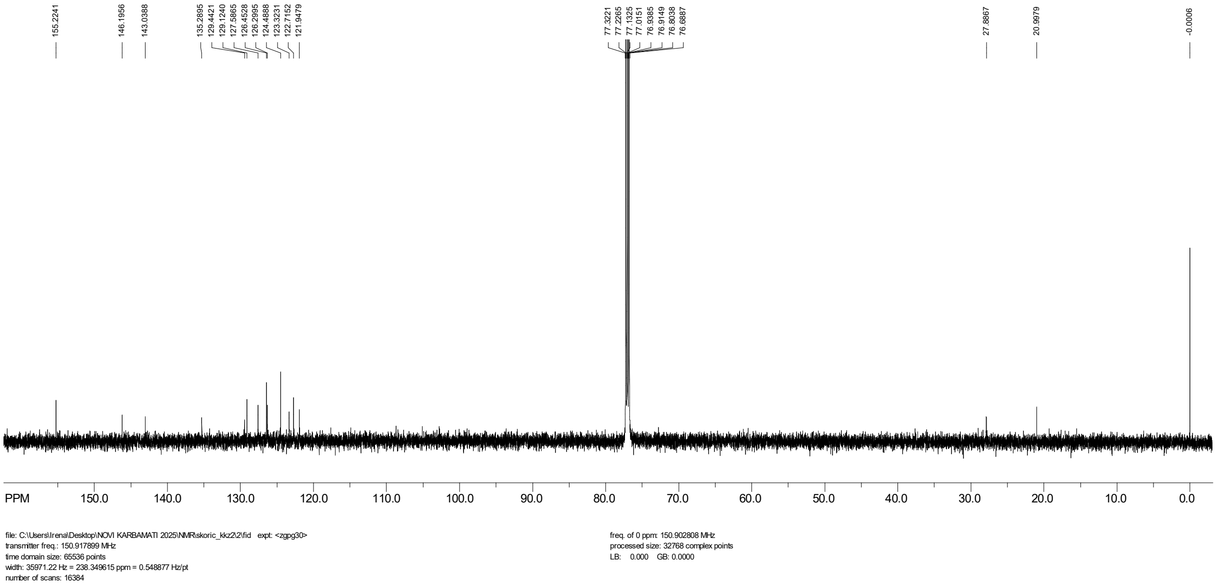

Figure S41. ^{13}C NMR spectrum ($CDCl_3$) of carbamate **4**.

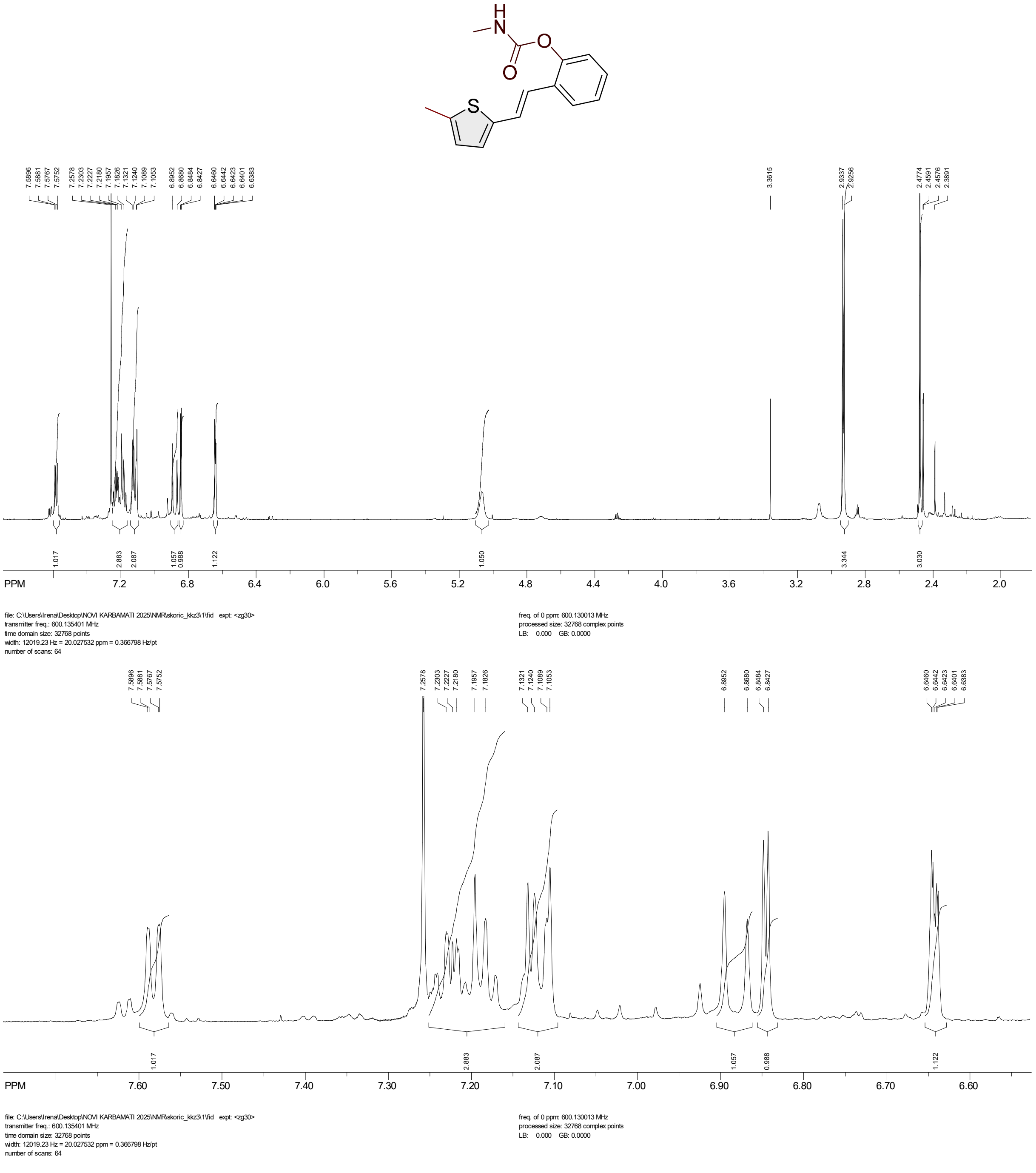

Figure S42. ^{1}H NMR spectrum ($CDCl_3$) of carbamate **5**.

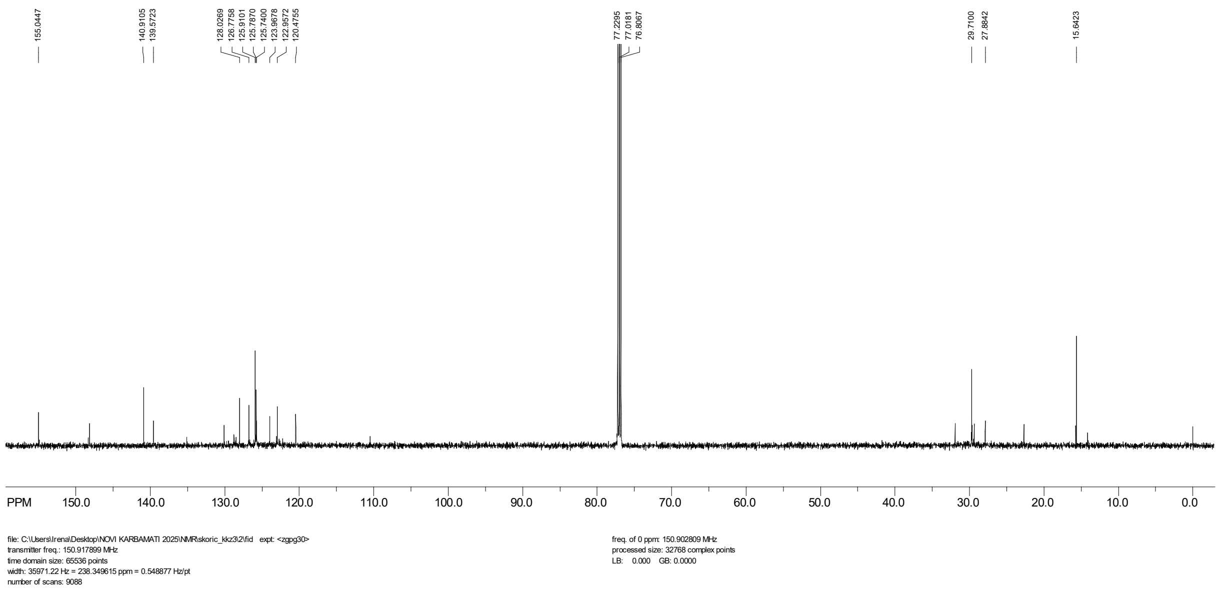

Figure S43. ^{13}C NMR spectrum ($CDCl_3$) of carbamate **5**.

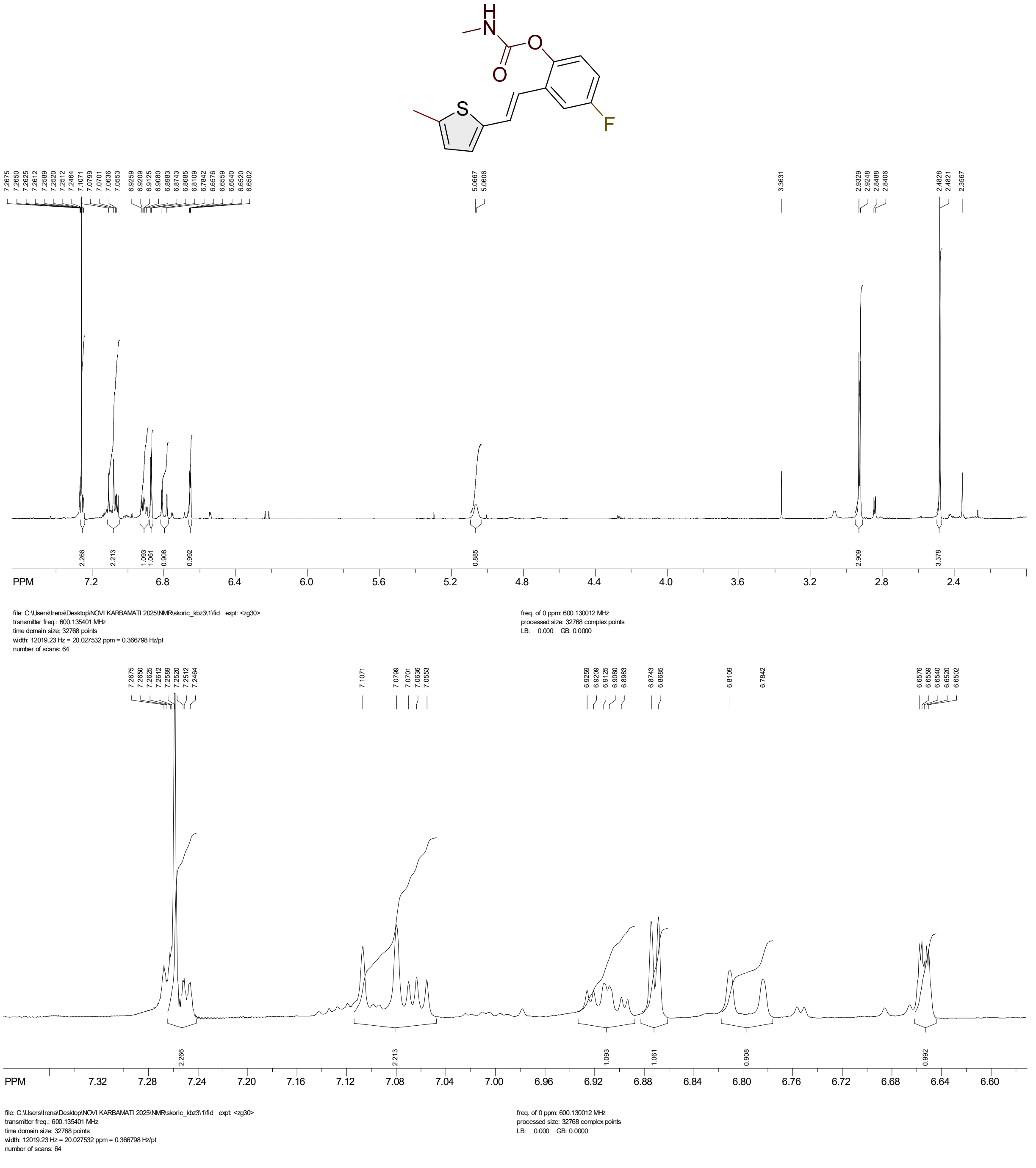

Figure S44. ¹H NMR spectrum ($CDCl_3$) of carbamate **6**.

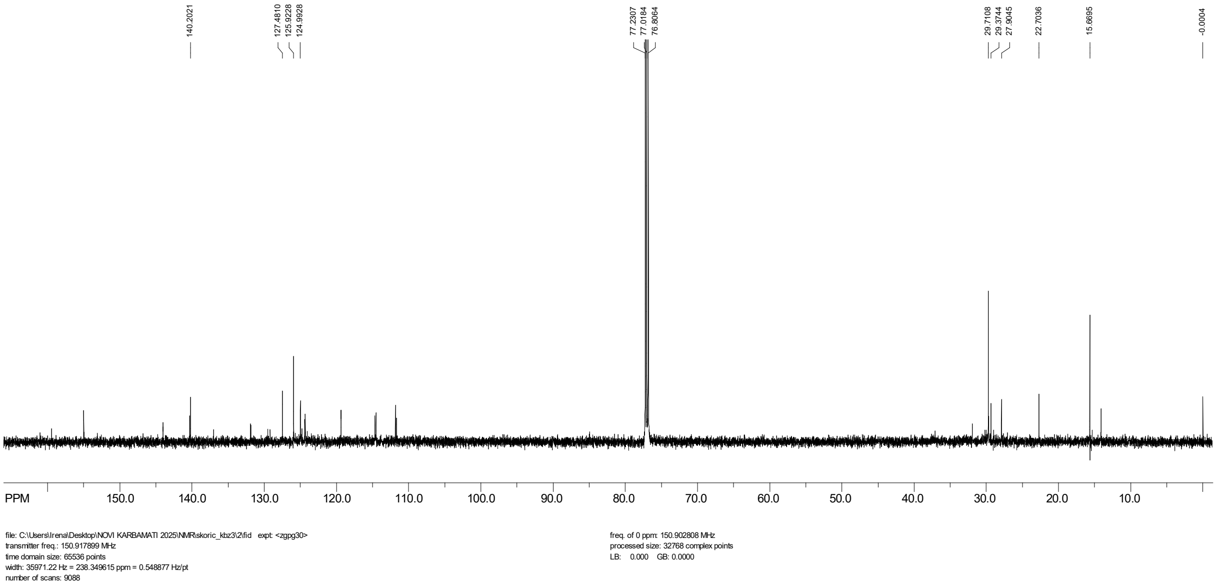

Figure S45. ^{13}C NMR spectrum ($CDCl_3$) of carbamate **6**.

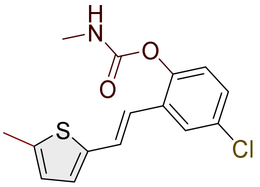

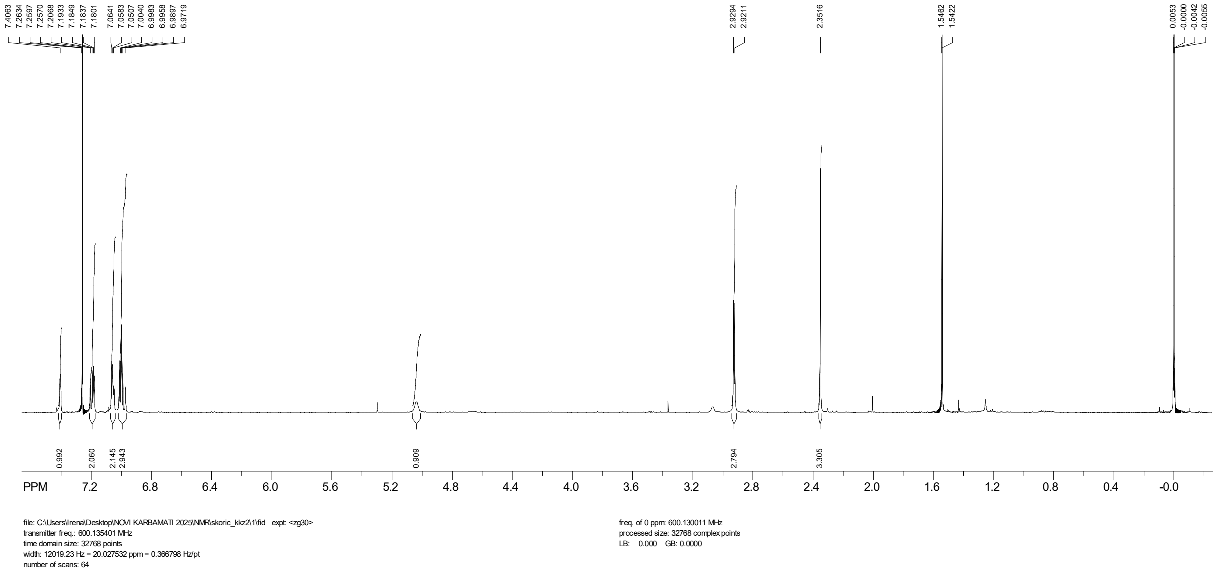

Figure S46. ^{1}H NMR spectrum ($CDCl_3$) of carbamate **7**.

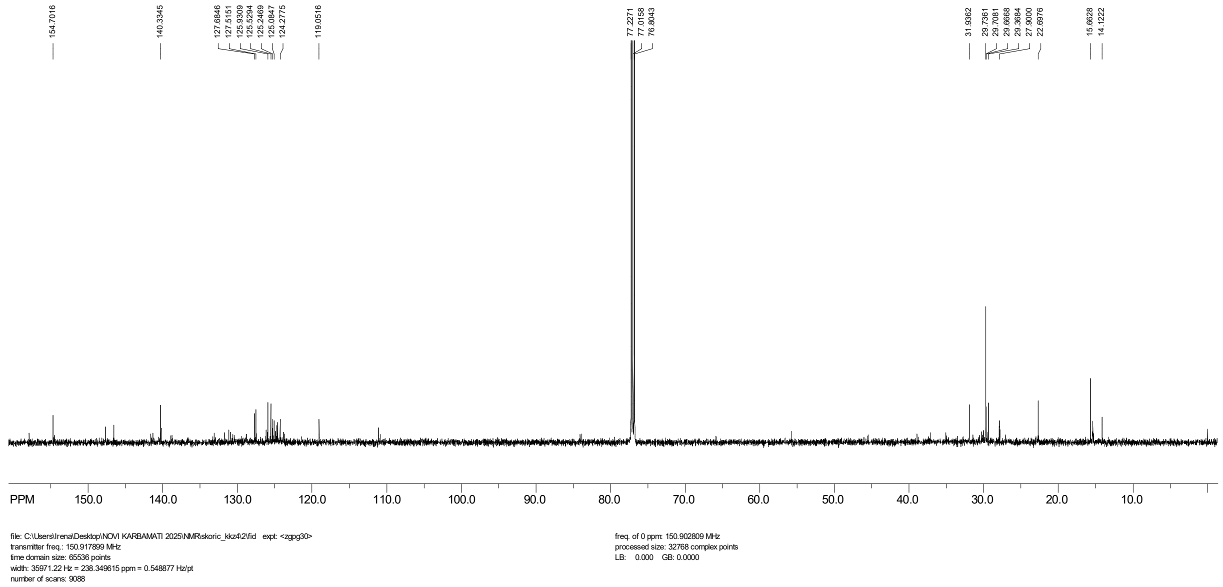

Figure S47. ^{13}C NMR spectrum ($CDCl_3$) of carbamate **7**.

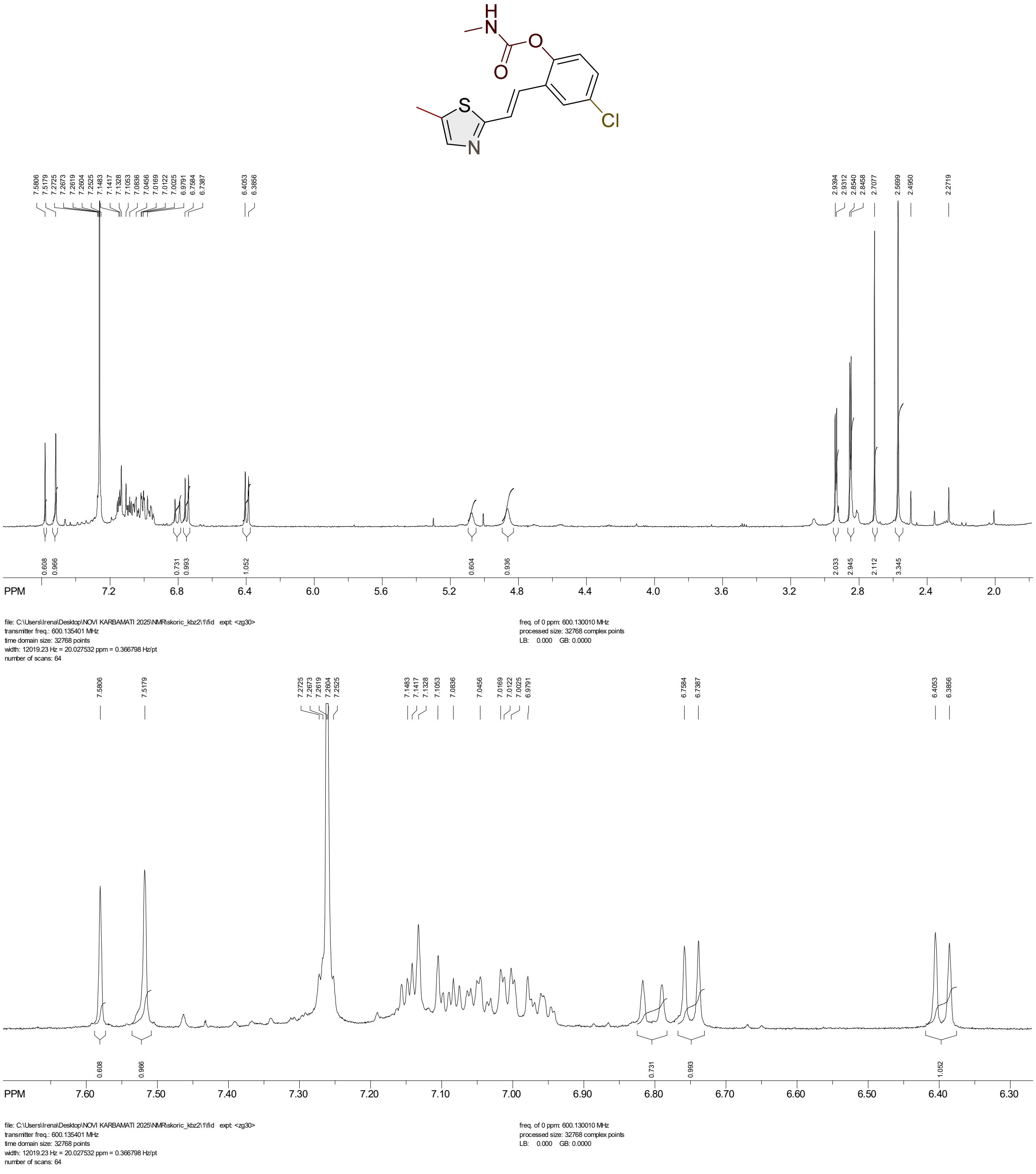

Figure S48. ^{1}H NMR spectrum ($CDCl_3$) of the mixture of (*E*)- and (*Z*)-isomer of carbamate **8**.

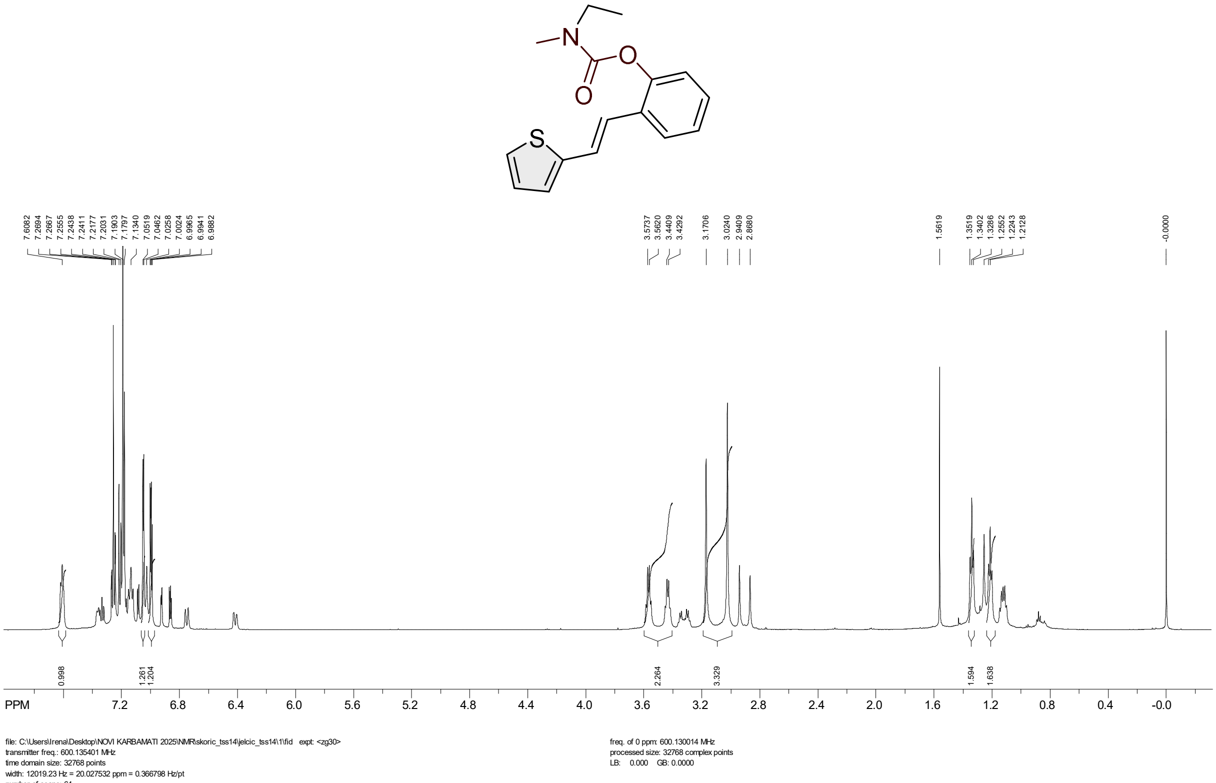

Figure S49. ^{1}H NMR spectrum ($CDCl_3$) of carbamate **9** (with small amount of (*Z*)-isomer).

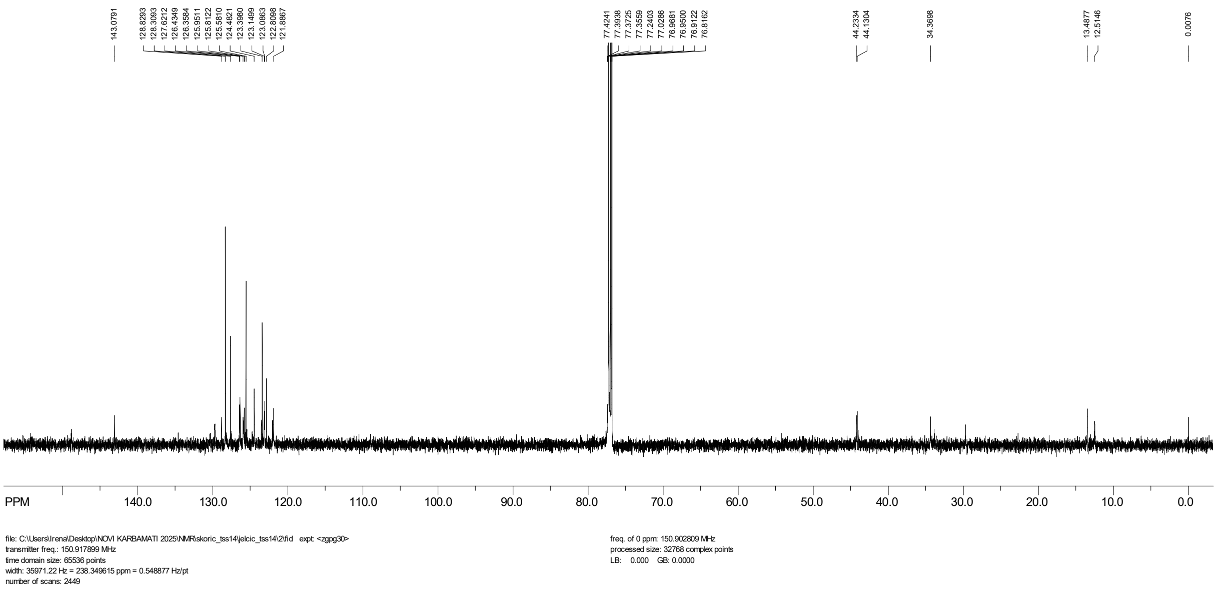

Figure S50. ^{13}C NMR spectrum ($CDCl_3$) of carbamate **9**.

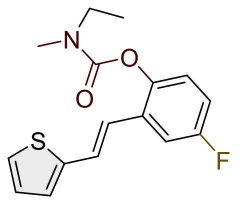

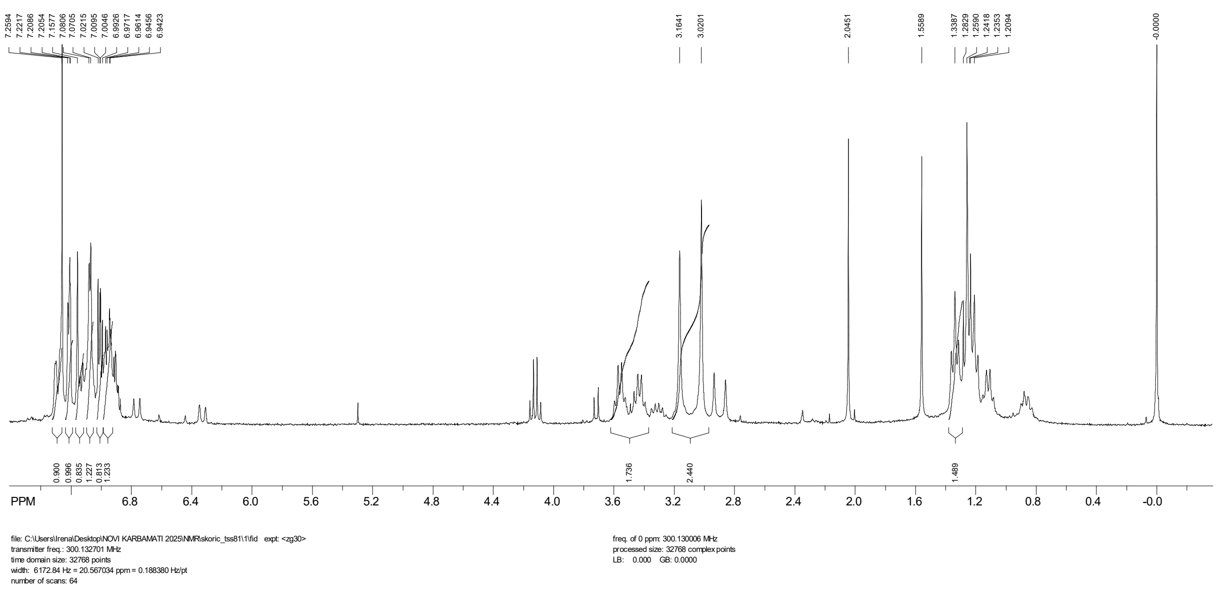

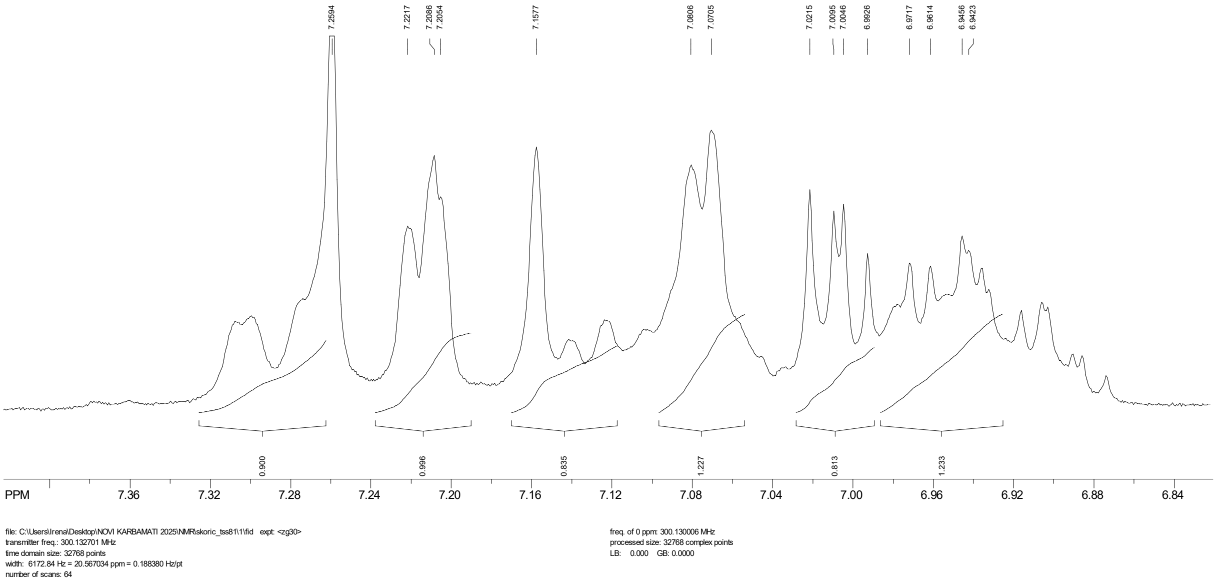

Figure S51. ^{1}H NMR spectrum ($CDCl_3$) of carbamate **10**.

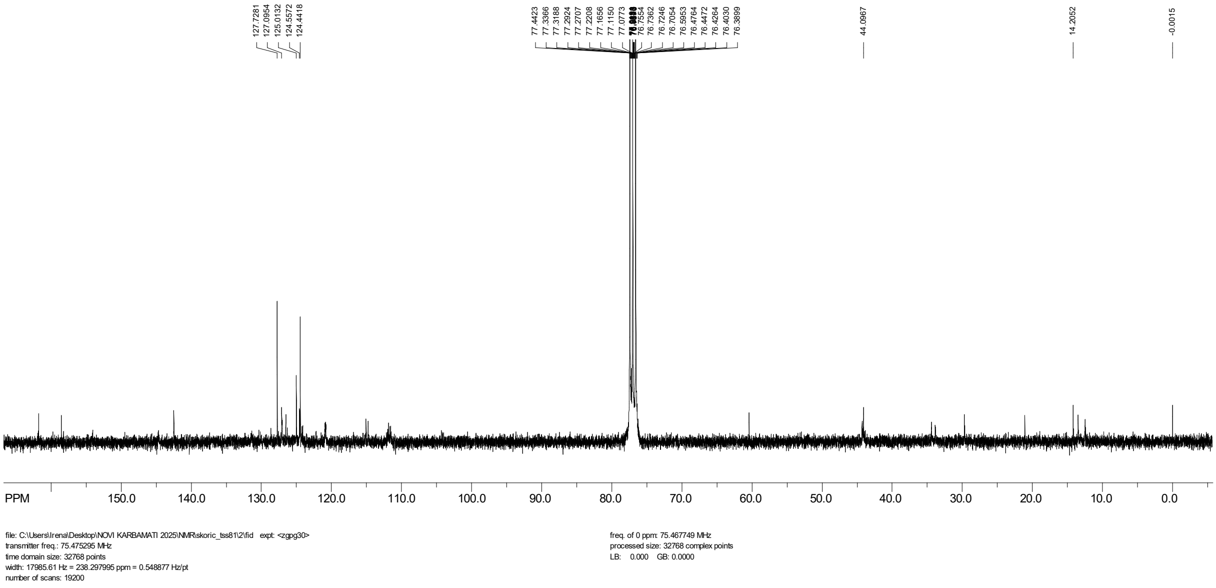

Figure S52. ^{13}C NMR spectrum ($CDCl_3$) of carbamate **10**.

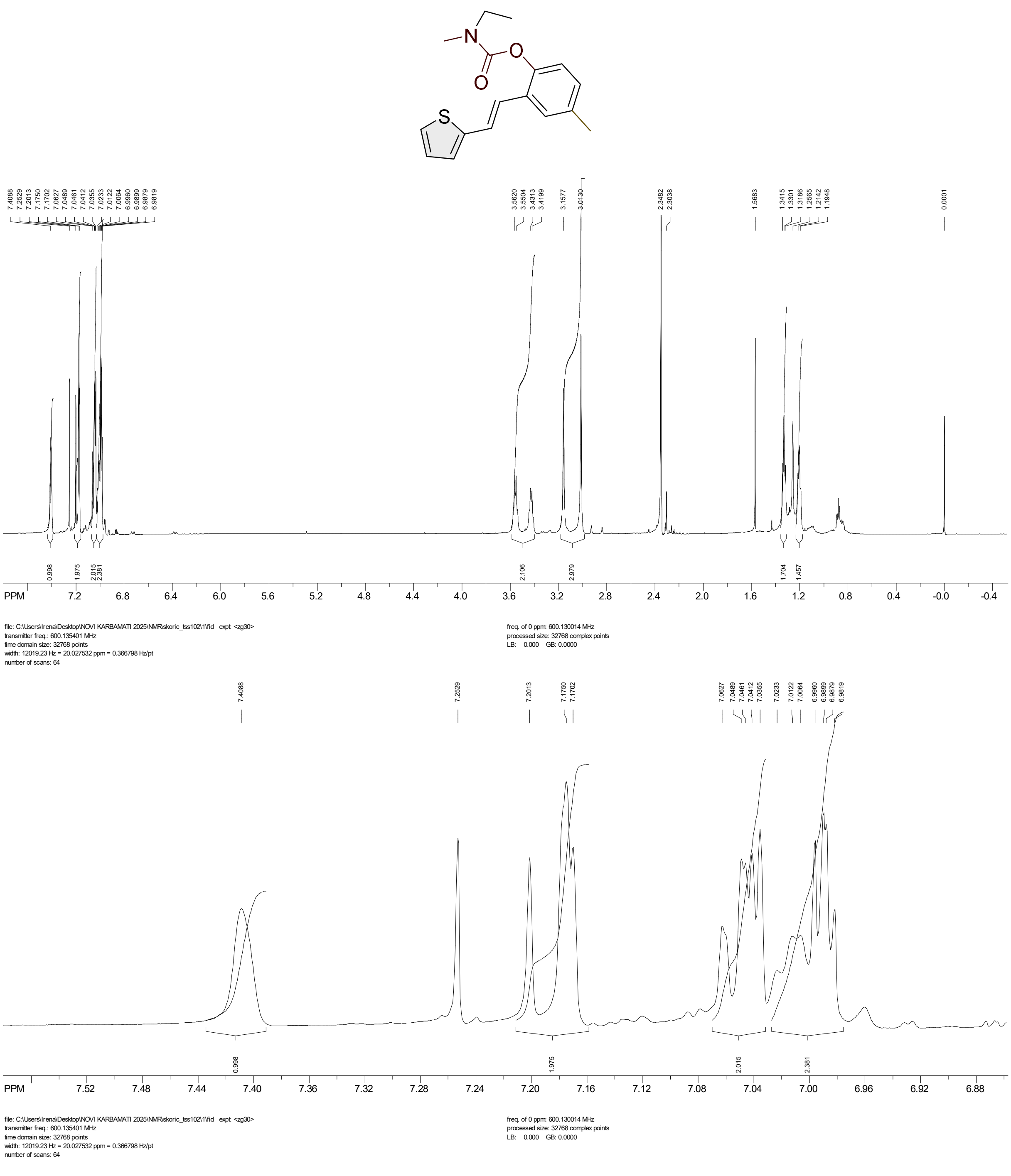

Figure S53. ^{1}H NMR spectrum ($CDCl_3$) of carbamate **11**.

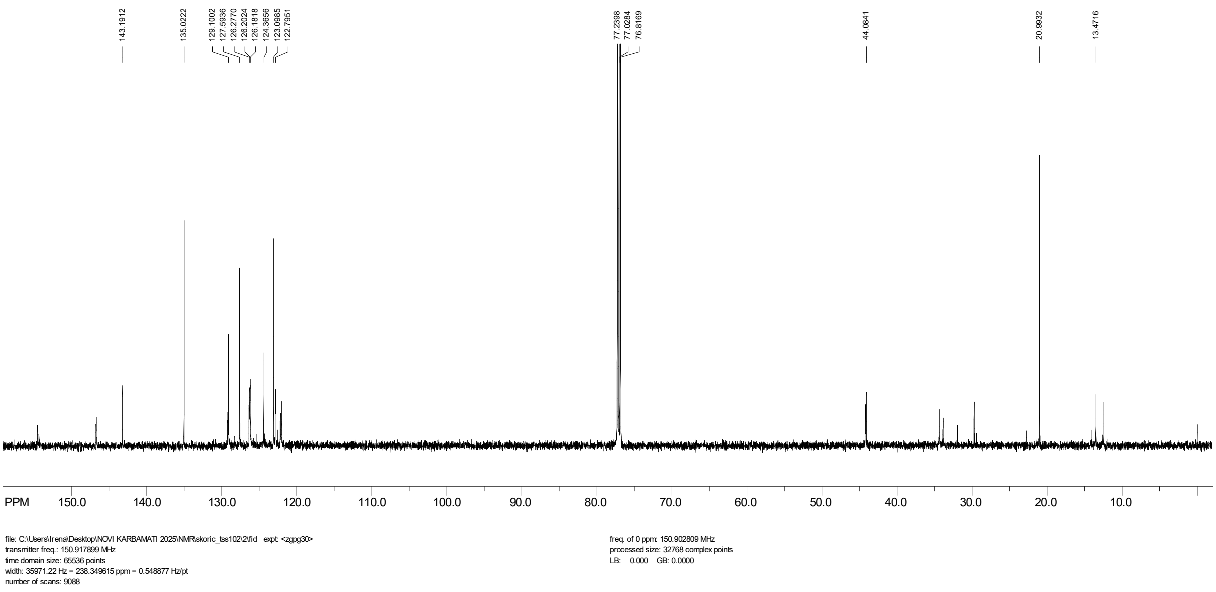

Figure S54. ^{13}C NMR spectrum ($CDCl_3$) of carbamate **11**.

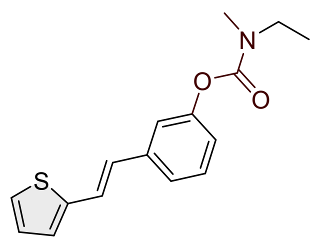

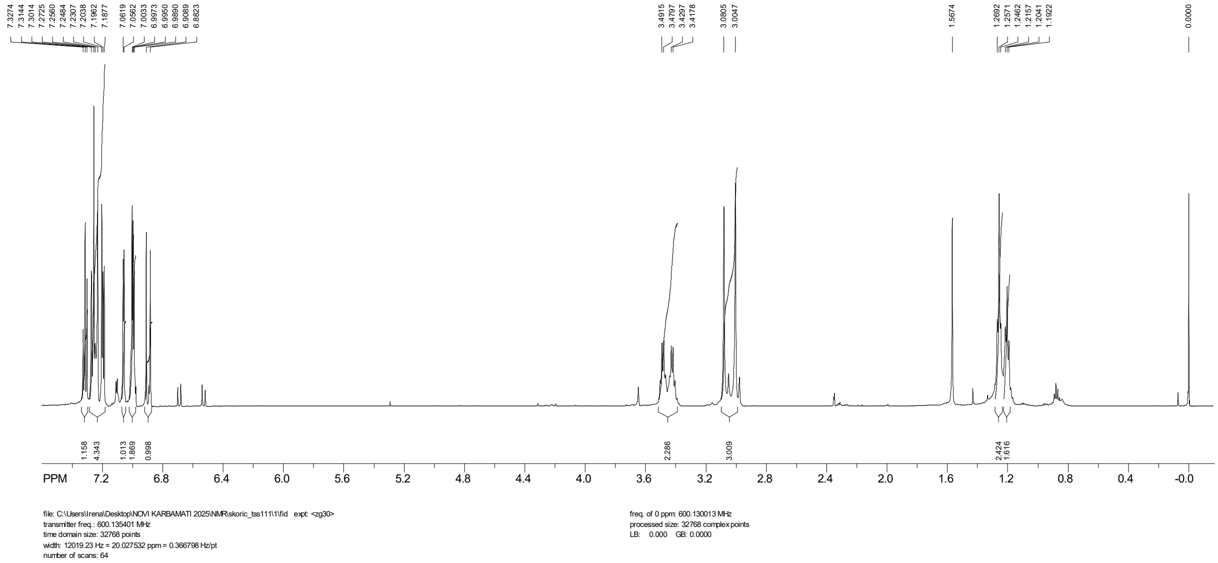

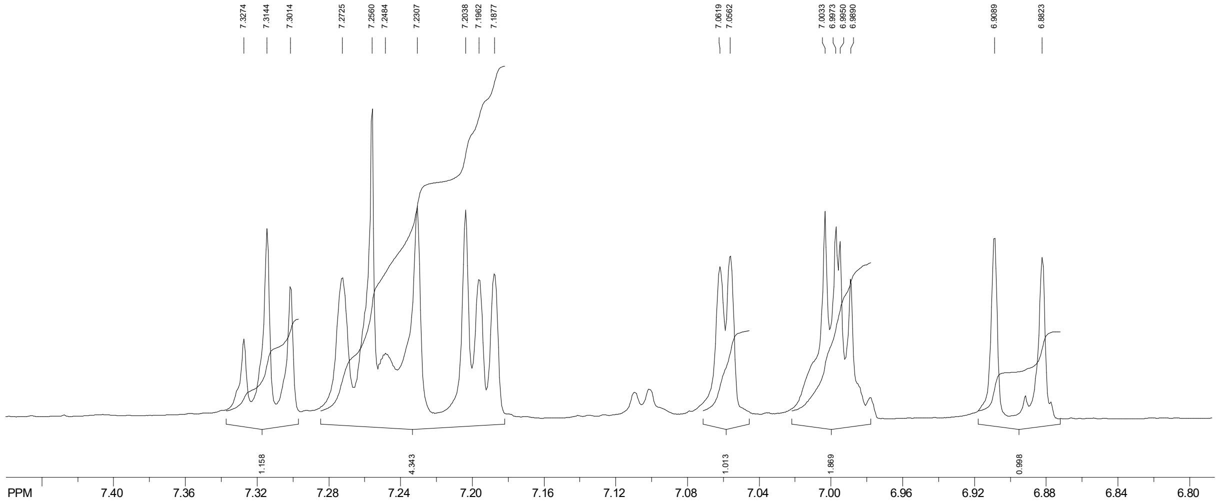

Figure S55. ^{1}H NMR spectrum ($CDCl_3$) of carbamate **12**.

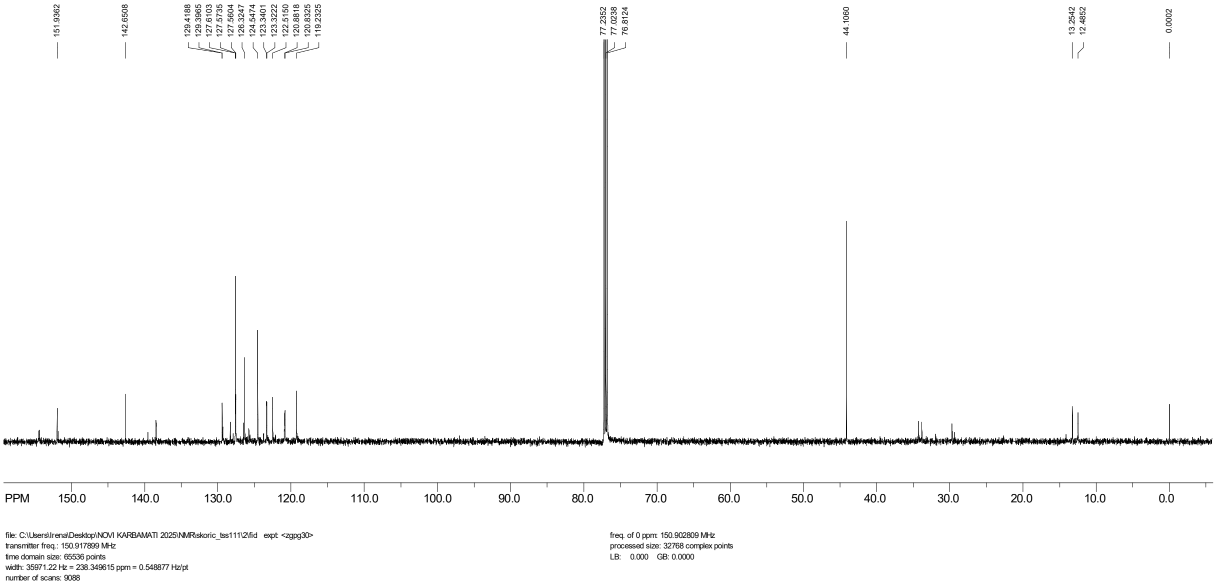

Figure S56. ^{13}C NMR spectrum ($CDCl_3$) of carbamate **12**.

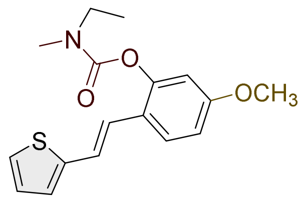

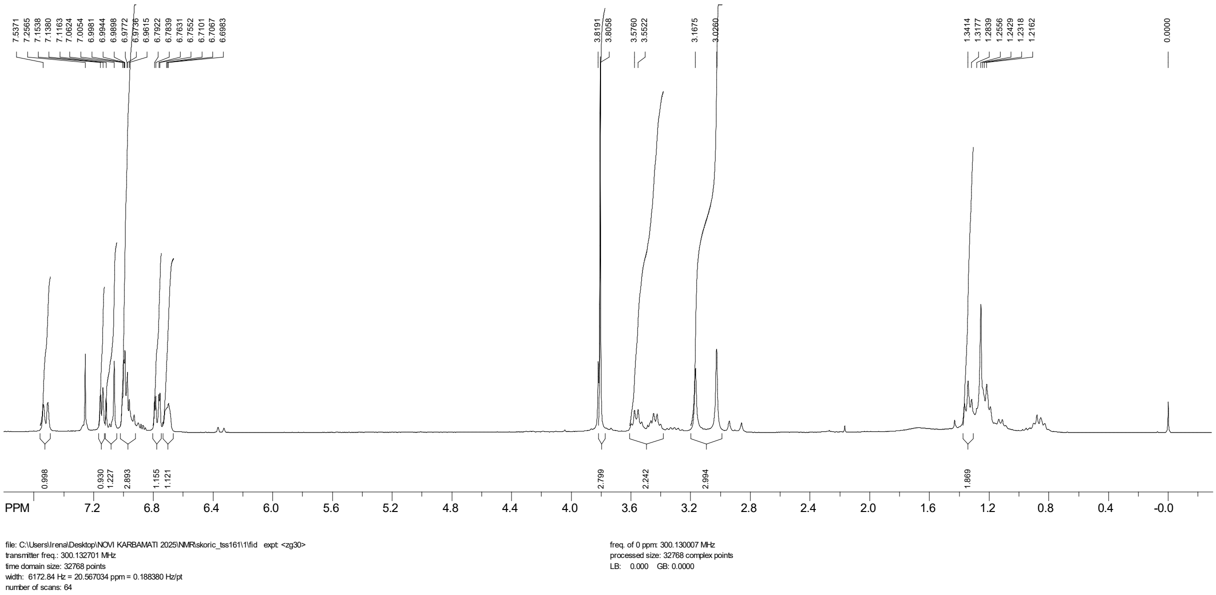

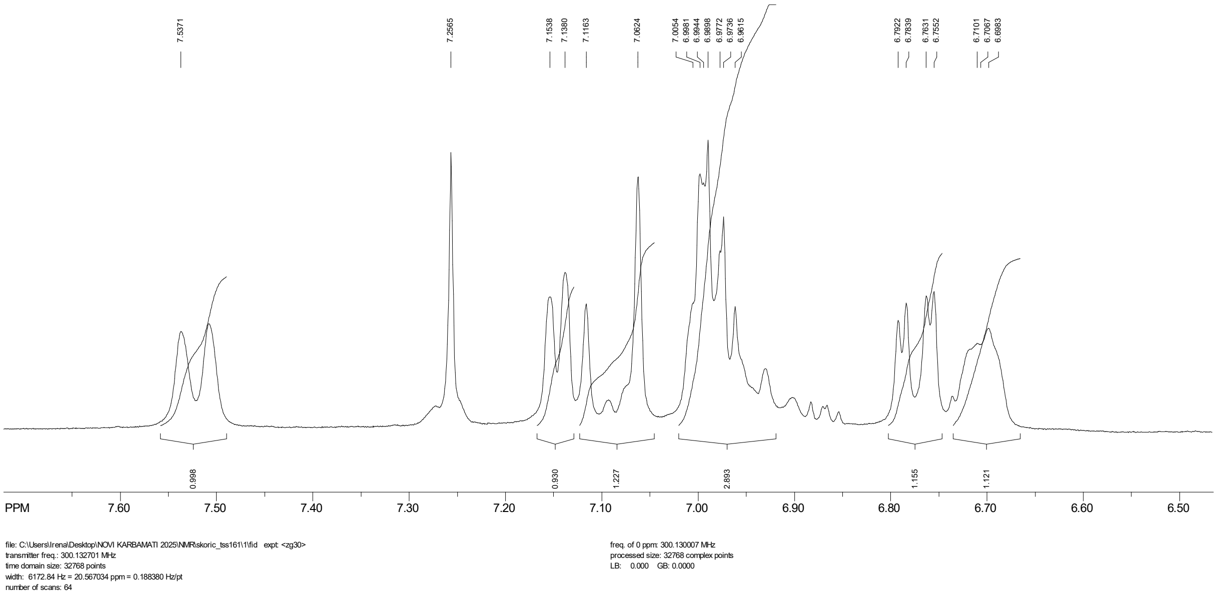

Figure S57. ^{1}H NMR spectrum ($CDCl_3$) of carbamate **13**.

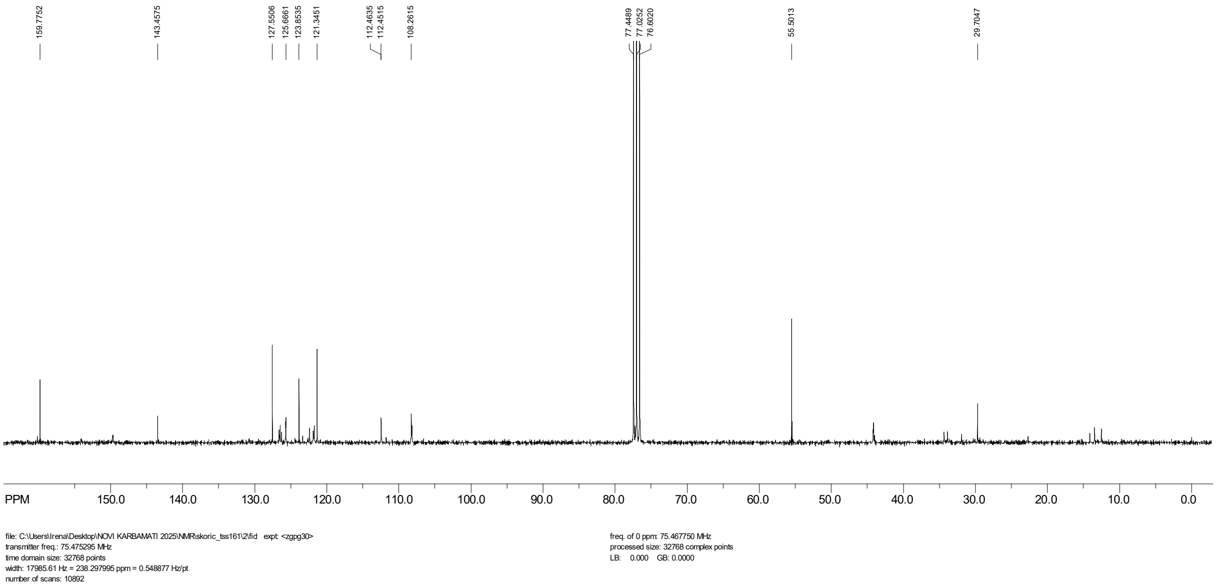

Figure S58. ^{13}C NMR spectrum ($CDCl_3$) of carbamate **13**.

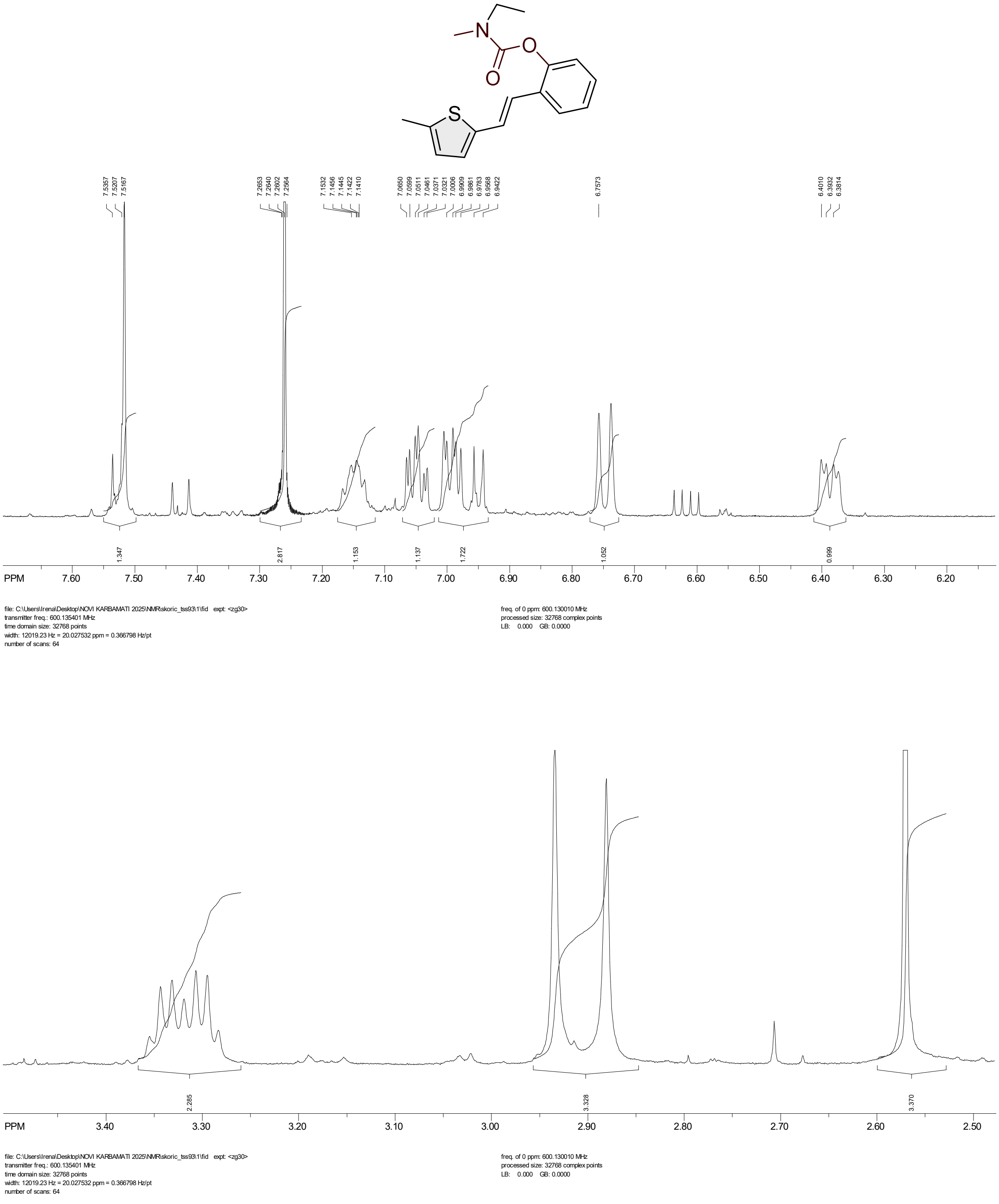

Figure S59. ^{1}H NMR spectrum ($CDCl_3$) of carbamate **14**.

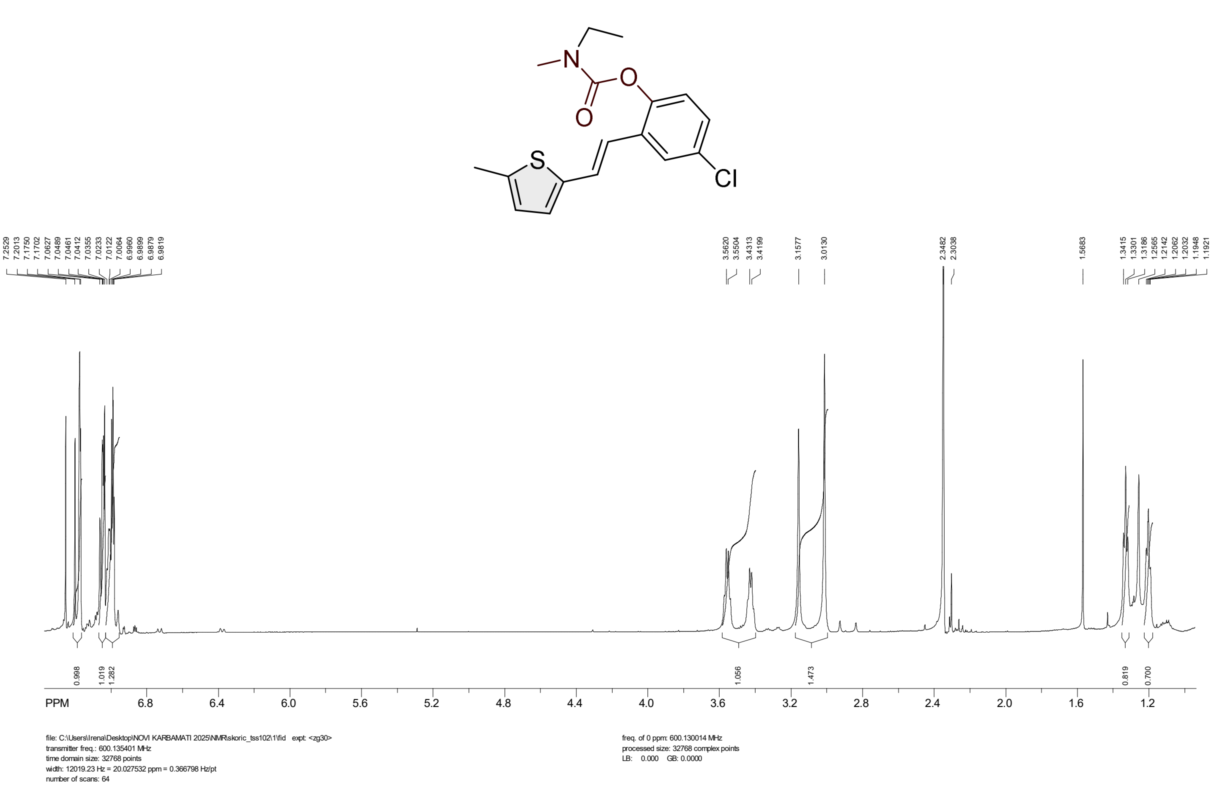

Figure S60. ^{1}H NMR spectrum ($CDCl_3$) of carbamate **15**.

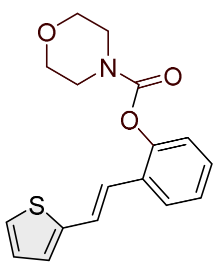

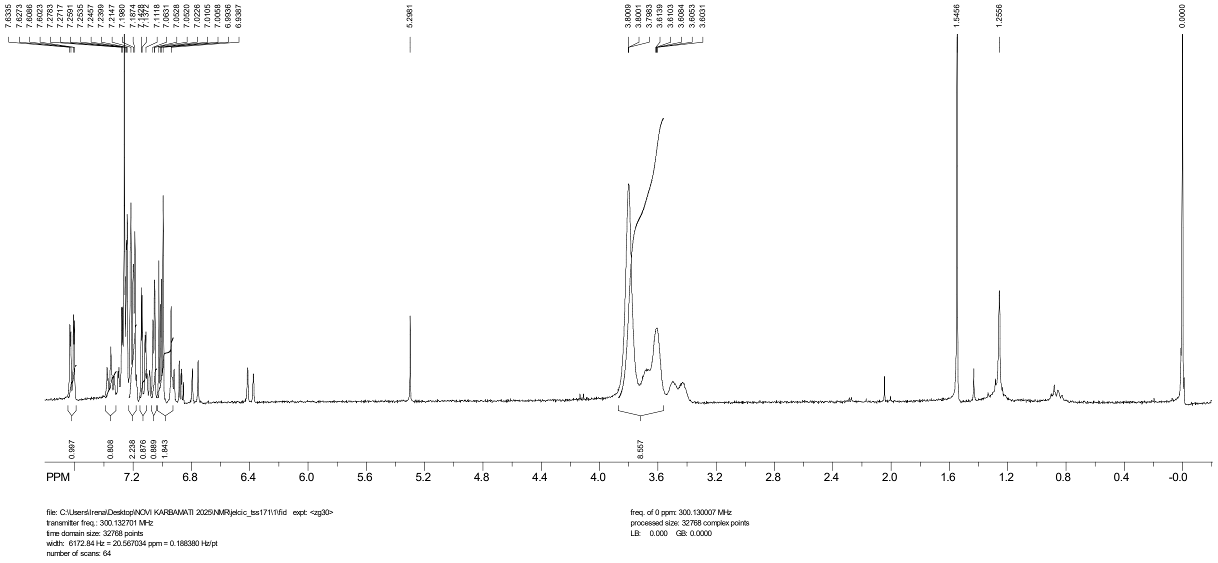

Figure S61. ^{1}H NMR spectrum ($CDCl_3$) of carbamate **16** (with a small proportion of (*Z*)-isomer).

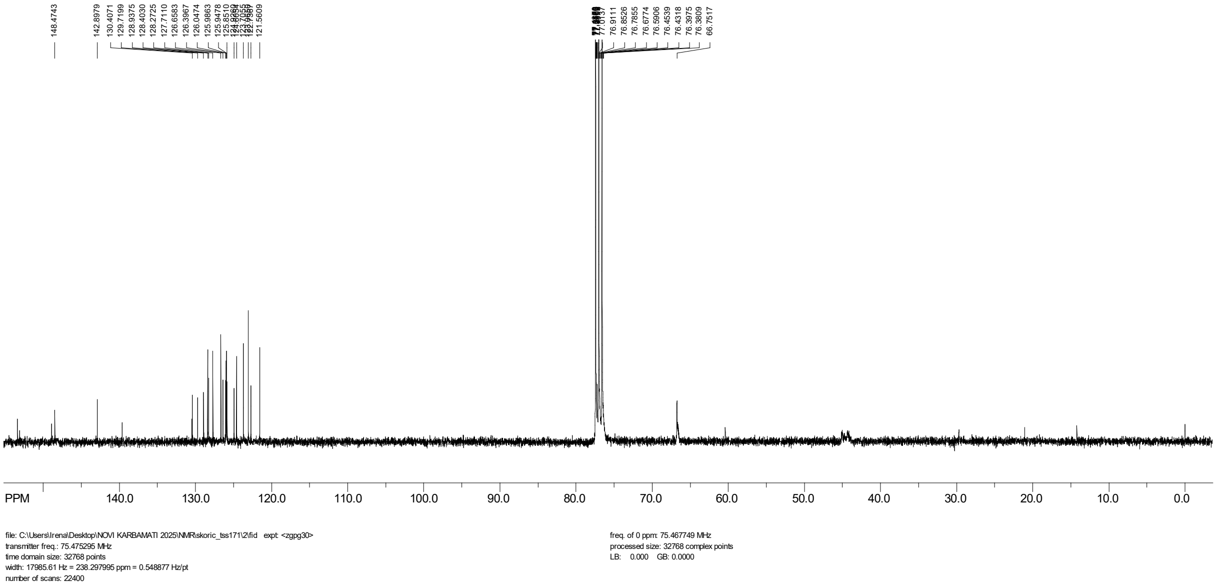

Figure S62. ^{13}C NMR spectrum ($CDCl_3$) of carbamate **16**.

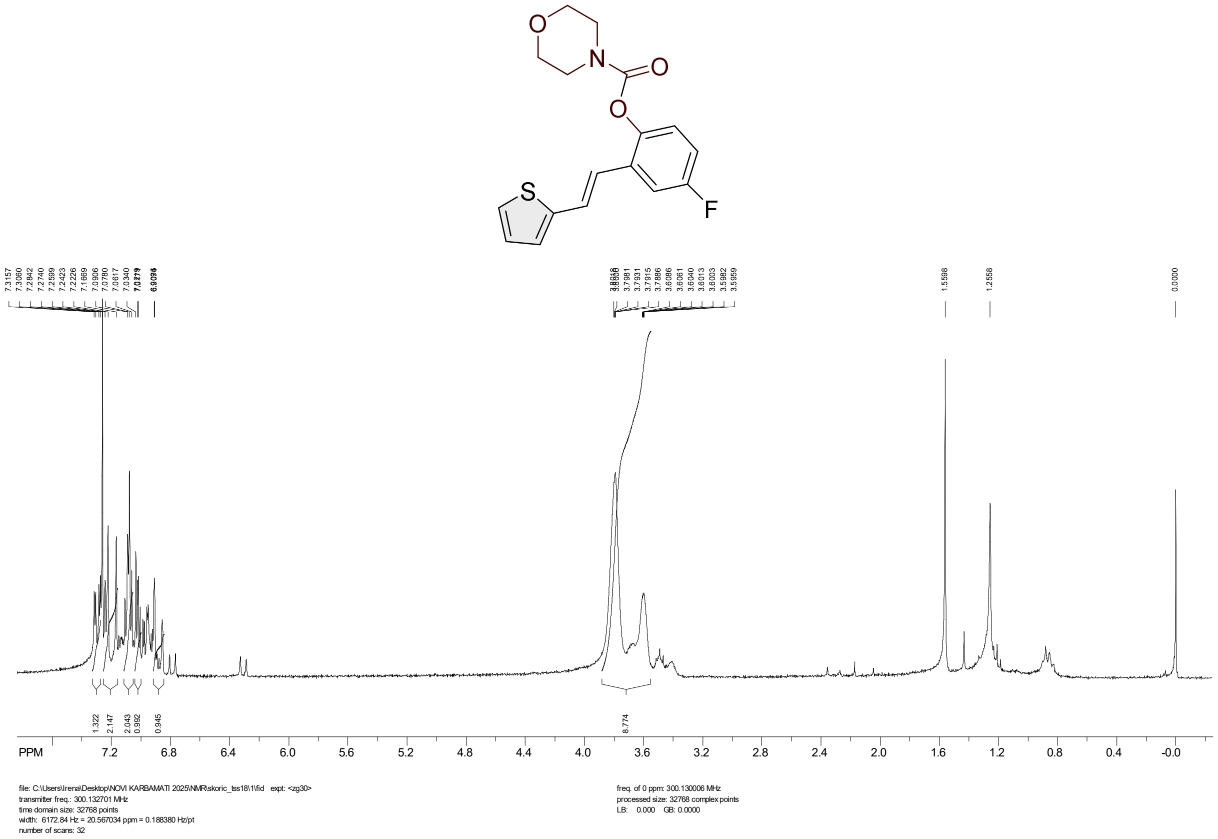

Figure S63. ^{1}H NMR spectrum ($CDCl_3$) of carbamate **17** (with a small proportion of (*Z*)-isomer).

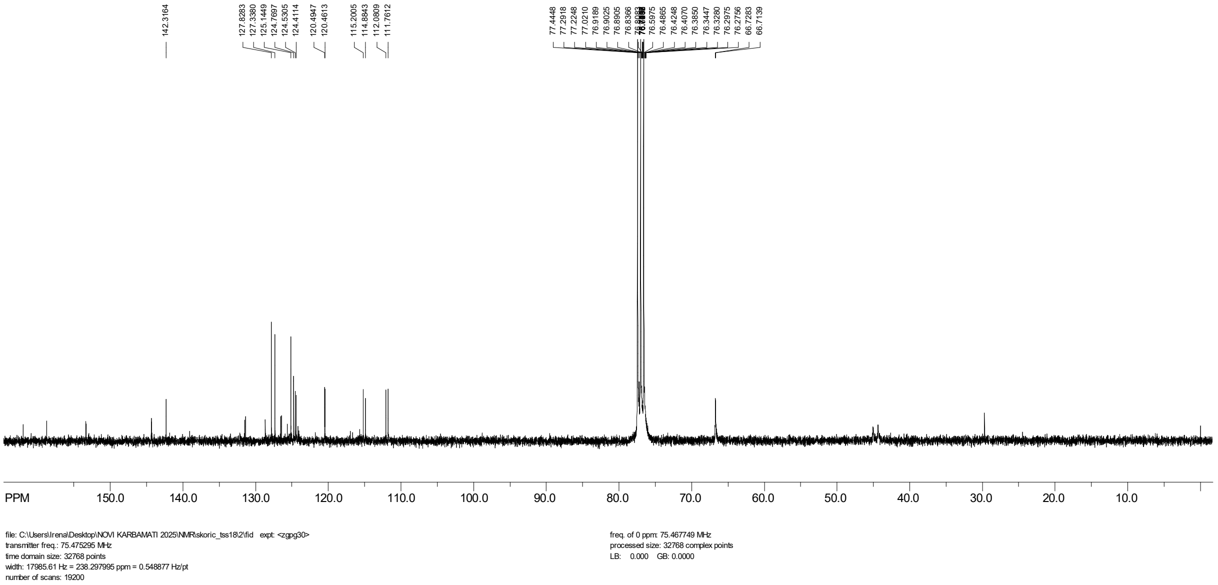

Figure S64. ^{13}C NMR spectrum ($CDCl_3$) of carbamate **17**.

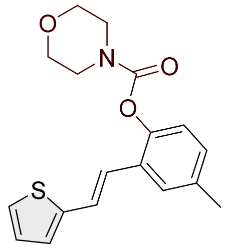

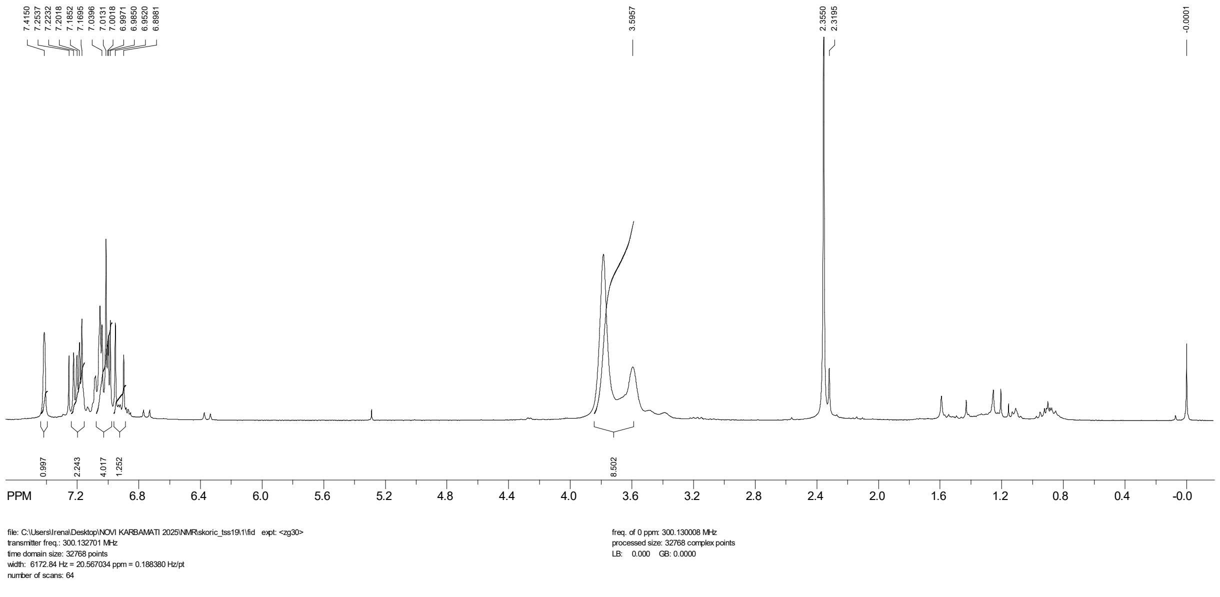

Figure S65. ^{1}H NMR spectrum ($CDCl_3$) of carbamate **18**.

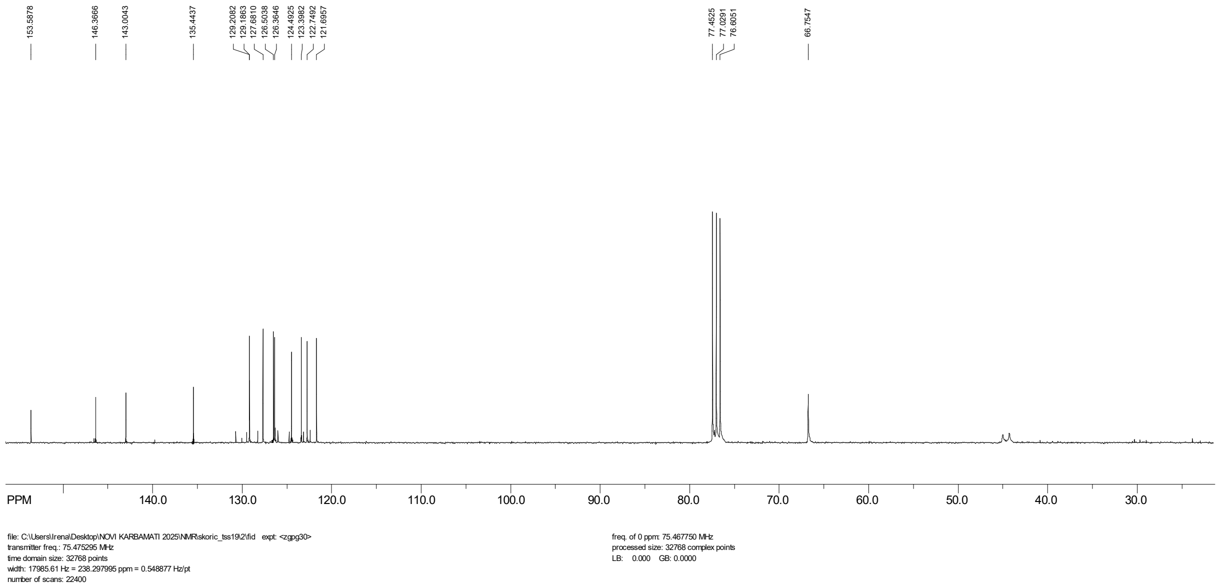

Figure S66. ^{13}C NMR spectrum ($CDCl_3$) of carbamate **18**.

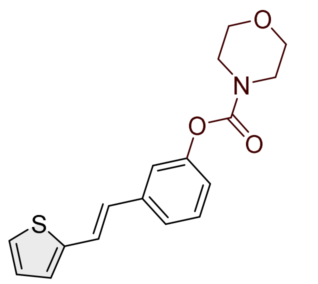

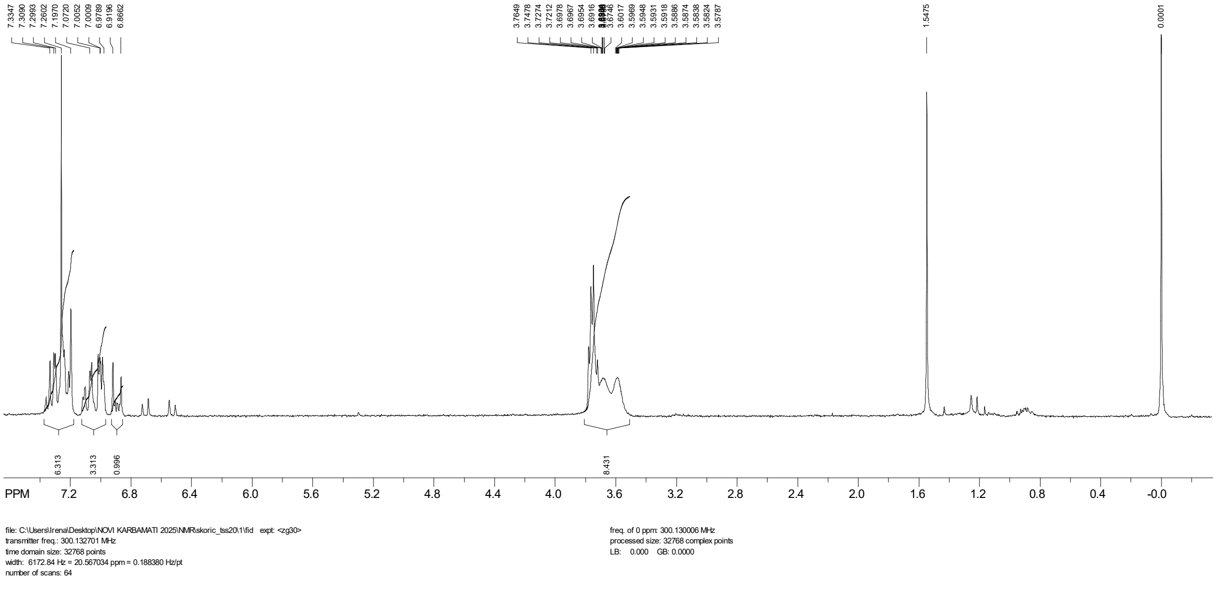

Figure S67. ^{1}H NMR spectrum ($CDCl_3$) of carbamate **19** (with a small proportion of (*Z*)-isomer).

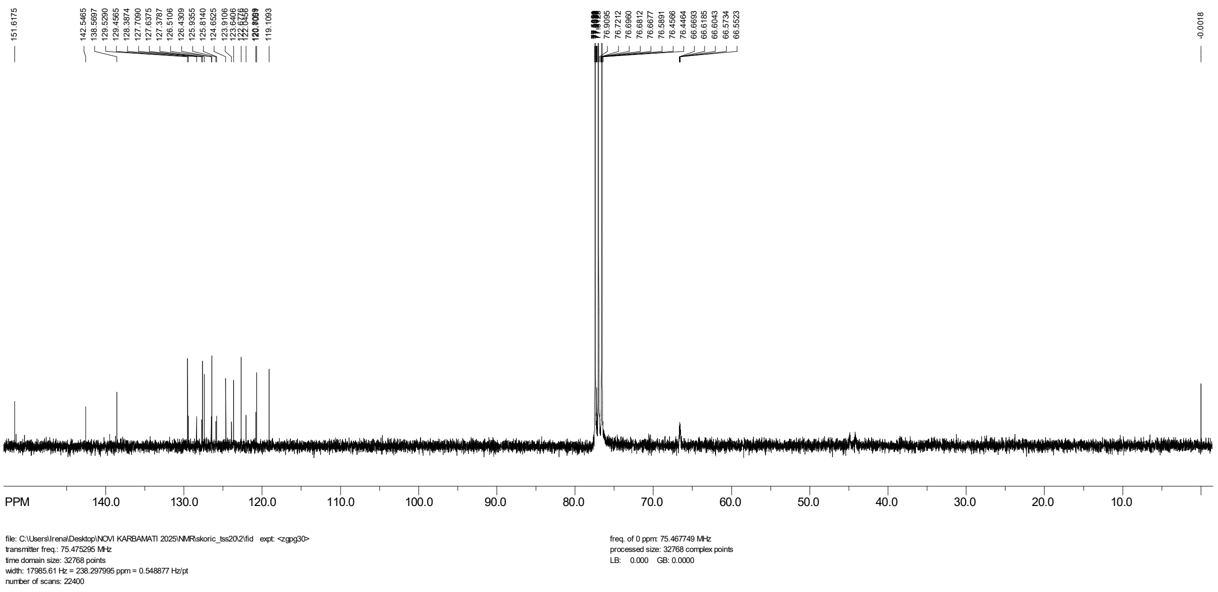

Figure S68. ^{13}C NMR spectrum ($CDCl_3$) of carbamate **19**.

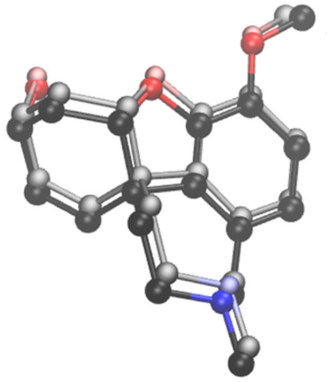

Figure S69. The docking protocol was validated as a cross-docking of galantamine into the active site of AChE, using the crystal structure of the AChE-galantamine complex (PDB ID: 1QTI) as a reference. Heavy atom RMSD between predicted pose and experimental reference is 0.304 A. Heavy atoms of the crystal structure are presented in black (C), red (O), and blue (N), while pale colors depict heavy atoms of the docked ligand.

Cartesian coordinates of ligands docked into the active site of BChE

Compound **1**, ligand pose in the most stable complex

```
C    -22.017  -7.574  41.381
H    -21.936  -6.529  41.726
C    -20.957  -8.285  40.872
S    -21.460  -9.889  40.428
C    -23.078  -9.585  40.937
H    -23.893 -10.326  40.879
C    -23.232  -8.318  41.420
H    -24.187  -7.916  41.797
C    -19.595  -7.807  40.709
H    -19.443  -6.714  40.705
C    -18.503  -8.577  40.563
H    -18.643  -9.671  40.588
C    -17.139  -8.071  40.373
C    -16.812  -6.711  40.484
H    -17.606  -5.982  40.717
C    -15.509  -6.264  40.307
H    -15.275  -5.191  40.406
C    -14.495  -7.175  40.004
H    -13.460  -6.821  39.863
C    -14.789  -8.528  39.880
H    -13.992  -9.253  39.642
C    -16.099  -8.957  40.059
O    -16.421 -10.306  40.026
C    -17.001 -10.829  38.898
O    -18.088 -10.517  38.471
N    -16.159 -11.734  38.348
H    -15.316 -11.956  38.855
C    -16.563 -12.527  37.203
H    -15.869 -13.273  36.750
H    -16.897 -11.830  36.399
H    -17.514 -13.050  37.461
```

Compound **1**, the pose of the ligand in the most populated conformational cluster

C	-21.815	-7.771	41.436
H	-21.339	-6.873	41.864
C	-21.132	-8.708	40.699
S	-22.205	-9.977	40.187
C	-23.549	-9.237	40.972
H	-24.570	-9.654	40.972
C	-23.199	-8.073	41.594
H	-23.906	-7.439	42.155
C	-19.717	-8.685	40.371
H	-19.362	-9.417	39.626
C	-18.802	-7.852	40.894
H	-19.152	-7.102	41.623
C	-17.372	-7.865	40.568
C	-16.635	-6.692	40.342
H	-17.143	-5.715	40.403
C	-15.280	-6.737	40.043
H	-14.721	-5.801	39.875
C	-14.625	-7.967	39.955
H	-13.548	-8.003	39.719
C	-15.330	-9.147	40.165
H	-14.819	-10.122	40.097
C	-16.686	-9.083	40.462
O	-17.408	-10.230	40.761
C	-17.420	-10.698	42.050
O	-16.437	-10.870	42.732
N	-18.700	-10.923	42.426
H	-19.418	-10.813	41.727
C	-19.003	-11.529	43.708
H	-20.058	-11.714	44.018
H	-18.509	-10.924	44.503
H	-18.447	-12.493	43.777

Compound **16**, ligand pose in the most stable complex

C	-18.786	-8.376	41.153
H	-19.607	-7.734	40.792
C	-19.048	-9.442	42.005
C	-18.034	-10.291	42.475
C	-16.721	-10.011	42.068
H	-15.896	-10.648	42.428
C	-16.440	-8.948	41.221
H	-15.400	-8.749	40.911
C	-17.475	-8.130	40.762
H	-17.251	-7.286	40.087
O	-20.342	-9.791	42.365
C	-21.096	-10.573	41.535
N	-21.958	-9.806	40.813
C	-22.732	-10.451	39.757
H	-22.816	-11.553	39.905
H	-22.191	-10.465	38.782
C	-21.866	-8.353	40.701
H	-21.310	-7.895	41.552

H	-21.184	-8.032	39.880
C	-24.092	-9.774	39.656
H	-24.724	-10.238	38.863
H	-24.732	-10.004	40.540
O	-23.953	-8.379	39.462
C	-23.271	-7.782	40.548
H	-23.853	-7.869	41.495
H	-23.254	-6.671	40.459
O	-21.011	-11.780	41.476
C	-18.371	-11.413	43.359
H	-19.360	-11.884	43.231
C	-17.565	-11.910	44.312
H	-16.571	-11.445	44.424
C	-17.891	-13.008	45.208
C	-18.681	-14.093	44.916
H	-19.160	-14.247	43.935
C	-18.820	-15.000	46.007
H	-19.415	-15.927	45.962
C	-18.133	-14.591	47.113
H	-18.110	-15.146	48.066
S	-17.305	-13.103	46.842

Compound **16**, the pose of the ligand in the most populated conformational cluster

C	-18.304	-13.244	44.538
H	-17.391	-12.844	45.010
C	-19.108	-12.426	43.752
C	-20.269	-12.900	43.123
C	-20.612	-14.243	43.333
H	-21.526	-14.647	42.866
C	-19.824	-15.074	44.118
H	-20.111	-16.129	44.266
C	-18.668	-14.574	44.720
H	-18.042	-15.236	45.342
O	-18.757	-11.115	43.464
C	-17.564	-10.845	42.852
N	-17.660	-9.712	42.105
C	-16.441	-9.177	41.507
H	-15.903	-8.480	42.191
H	-15.642	-9.948	41.410
C	-18.760	-8.756	42.202
H	-18.593	-7.995	42.999
H	-19.687	-9.215	42.617
C	-16.786	-8.529	40.173
H	-17.066	-9.289	39.407
H	-15.882	-8.110	39.673
O	-17.796	-7.550	40.330
C	-18.986	-8.125	40.833
H	-19.820	-7.385	40.856
H	-19.424	-8.855	40.113
O	-16.575	-11.538	42.954
C	-21.070	-11.995	42.290
H	-21.072	-12.173	41.201
C	-21.800	-10.967	42.752
H	-21.805	-10.803	43.843
C	-22.584	-10.051	41.939
C	-22.200	-8.802	41.515

```
H   -21.220  -8.355  41.754
C   -23.194  -8.140  40.735
H   -23.068  -7.131  40.308
C   -24.318  -8.897  40.578
H   -25.207  -8.571  40.012
S   -24.184 -10.423  41.369
```

Galantamine

```
C  -22.383 -10.415  42.154 H  -21.530 -10.579  42.833
C  -23.576 -10.869  42.537
H  -23.638 -11.416  43.493
C  -24.849 -10.682  41.744
H  -25.645 -10.363  42.457
C  -24.690  -9.573  40.705
H  -25.558  -9.544  40.006
H  -24.804  -8.564  41.166
C  -23.380  -9.692  39.954
H  -23.306  -8.856  39.220
C  -22.117  -9.689  40.849
O  -23.347 -10.952  39.238
C  -21.518 -12.259  38.280
C  -20.140 -12.473  38.318
H  -19.688 -13.227  37.651
C  -19.324 -11.745  39.190
H  -18.237 -11.933  39.185
C  -18.946 -10.067  41.044
H  -17.881 -10.351  40.873
H  -19.095 -10.475  42.071
N  -19.082  -8.608  41.057
C  -20.251  -8.147  41.804
H  -20.314  -8.670  42.787
H  -20.099  -7.099  42.153
C  -21.576  -8.270  41.050
H  -22.350  -7.640  41.546
H  -21.496  -7.755  40.064
C  -22.024 -11.286  39.144
C  -21.215 -10.575  40.011
C  -19.839 -10.789  40.065
C  -17.877  -8.022  41.634
H  -17.980  -6.912  41.644
H  -16.951  -8.357  41.112
H  -17.646  -8.435  42.643
O  -25.267 -11.903  41.157
H  -25.086 -11.820  40.206
O  -22.409 -12.901  37.485
C  -21.920 -13.406  36.262
H  -22.653 -13.934  35.608
H  -21.045 -14.070  36.455
H  -21.431 -12.585  35.688
```

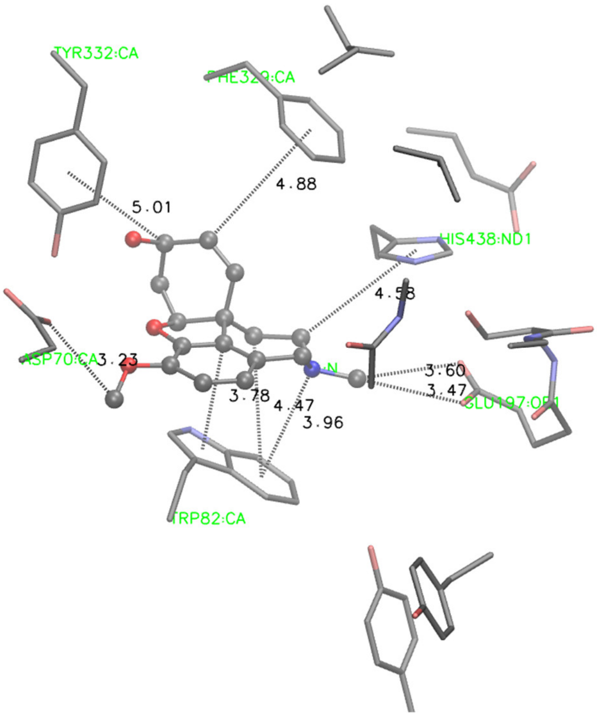

Figure S70. Galantamine docked in the active site of BChE. The ligand is involved in hydrophobic interactions with the choline-binding site (residue Trp82), and an electrostatic interaction between the tertiary amine of galantamine and Glu197. The bicyclic ring system reaches toward the peripheral site (Asp70 and Tyr332), stabilizing the pose via long-range electrostatic and hydrophobic contacts.

Table S1. Free energies of binding, ΔG_{bind}, obtained by molecular docking of ligands **1**, **16,** and reference ligand galantamine into the active site of BChE (7AIY.pdb), along with the number of conformational clusters and distribution of conformations.

Ligand	ΔG_{bind}/kcal mol^{-1}		Number of distinctive conformational clusters	Distribution of conformations within clusters with $n > 1$ (n = cluster population)
	lowest	highest		
**1**	−4.96	−4.55	5	3, 11, 9
**16**	−5.44	−5.38	6	6, 7, 5, 5
Galantamine	−5.20	−5.17	3	23

Table S2. Root-mean-square displacement (RMSD), RMS fluctuations of alpha carbons of the protein backbone, and radius of gyration for complexes of BChE and **1** and **16**, derived by molecular dynamics simulation of 40 ns.

Enzyme-ligand complex	RMSD/Å		RMSF/Å		Rg/Å	
	average	min, max	average	min, max	average	min, max
BChE-**1**(I)	2.02	0.78, 2.37	0.76	0.33, 2.62	23.14	22.86, 23.33
BChE-**1**(II)	2.05	0.80, 2.45	0.84	0.37, 4.65	23.04	22.86, 23.27
BChE-**16**(I)	1.93	0.87, 2.23	0.78	0.35, 3.89	22.99	22.78, 23.23
BChE-**16**(II)	2.17	0.89, 2.59	0.87	0.39, 2.65	23.11	22.81, 23.34
